# Comparative evaluation of 5 combination adjuvants on immunogenicity and efficacy of approved seasonal influenza vaccines

**DOI:** 10.1038/s41541-025-01339-y

**Published:** 2026-01-27

**Authors:** Jenny Hernandez-Davies, Jiin Felgner, Erwin Strahsburger, Jacob Laster, Aarti Jain, Timothy Yates, Emily Silzel, Rafael Assis, Rie Nakajima, Algimantas Jasinskas, Andriy Yeromin, Egest J. Pone, Sharon Jan, Luis M. de la Maza, Li Liang, Philip Felgner, Lisa E. Wagar, Anthony E. Gregory, D. Huw Davies

**Affiliations:** 1 Adeline Mah Vaccine Center, Department of Physiology & Biophysics, University of California Irvine, Irvine, CA 92693 USA; 2 Present Address: Sandia National Laboratories, Livermore, CA 94550 USA

**Keywords:** Infectious diseases, Influenza virus, Vaccines, Adjuvants

## Abstract

The benefits of adjuvants for enhancing vaccine immunogenicity and efficacy are well known. Numerous novel adjuvants are at advanced levels of characterization, including some in clinical trials. However, understanding the relative benefits of each is hindered by a lack of comparative studies between adjuvants within the same study. To address this, we have performed a side-by-side comparison of 5 novel combination adjuvants (Alhydroxyquim-II, T-VANT, TRAC-478, IVAX-1 and IVAX-3) by formulating each with two approved seasonal influenza vaccines, Flublok® and Fluzone HD®, and assessing immunogenicity and efficacy in female and male C57Bl/6 mice. Although all tested adjuvants were immunogenic and protective against H1N1 challenge, T-VANT, TRAC-478 and IVAX-1 and -3 were associated with systemic inflammatory cytokines and robust Th1 responses, while Alhydroxyquim-II elicited lower levels of inflammatory cytokines and a Th2 response. Greater morbidity after challenge was also detected in males compared to females. Side-by-side comparisons of existing and novel adjuvants with the same antigen and model system will help inform rational adjuvant selection and guide vaccine development for influenza and other infectious diseases.

## Introduction

Despite the availability of seasonal vaccination, influenza A and B viruses (IAV and IBV) continue to represent a major source of annual morbidity and mortality in humans. Globally, there are more than a billion cases of seasonal influenza each year, including 3-5 million cases of severe illness and 290,000-650,000 deaths^1^. IAVs evolve during replication owing to a low-fidelity RNA polymerase, which provides a pool of mutants from which variants that escape antibody neutralization can emerge (a process termed ‘antigenic drift’)^2^. The majority of seasonal vaccines are prepared from viruses grown in fertilized chicken eggs, which are then inactivated and enriched for membrane proteins (or “split”) by detergent extraction. Owing to these manufacturing processes, first developed in the 1930s, there is a lag-time of ~10 months required to manufacture sufficient doses of vaccine each season. The efficacy of seasonal vaccines typically ranges from 10 - 60% from year to year, according to how well the circulating influenza strains that emerge each season match those predicted for vaccine manufacturing^3^. An additional hurdle for influenza vaccination lies in the immunodominant, membrane-distal head domain of hemagglutinin (HA), which is where most neutralizing epitopes are located and therefore where the majority of amino acid substitutions accumulate. Antibodies are, therefore, typically variant-specific and readily evaded by antibody-driven antigenic drift. As a consequence, efficacy elicited by the vaccines gradually wanes as IAV evolves, requiring that the variants used to prepare the vaccine are modified and re-administered each season. Most seasonal influenza vaccines are administered without adjuvant and the vaccine response is generally weak and short-lived. For example, antibody-secreting plasma cells quickly increase in number in the bone marrow after vaccination, but these numbers wane to background levels within a year^4^. Finally, seasonal vaccines are given against a background of pre-existing immunity, which is thought to influence vaccine effectiveness. In particular, serological studies suggest that the antibodies elicited by seasonal vaccines are dominated by responses to epitopes in the original priming variant (so-called ‘original antigenic sin’)^5^.

Some of the shortcomings of existing seasonal vaccines may be overcome by formulating with adjuvants, which are known to enhance breadth and durability of the response^6,7^ and may help overcome original antigenic sin^8^. Until the late 1990s, aluminum hydroxide salt (‘alum’) was the only adjuvant approved for human use^9^. Alum generally fails to improve antibody responses to influenza vaccines^10–12^, which may be related to its known effect on protein conformation^13^. Progress with adjuvanted influenza vaccines began to gain traction after experiments with the squalene oil-in-water emulsion, MF-59® developed by Novartis^14^. MF-59®, used with the seasonal vaccine FluAd®, was first licensed in Italy in 1997, the first new adjuvant since alum in 70 years, and is now used in >30 countries worldwide. Compared to non-adjuvanted influenza vaccines, MF59® significantly enhances the magnitude, breadth, and durability of antibody responses, as well as the magnitude of T cell responses, in animal models^15–17^ and in humans^18,19^. MF59® has particular utility for enhancing the immunogenicity of influenza vaccines in the elderly where immune responsiveness is typically lower than in younger adults^20^. MF59 has an excellent safety profile in all age groups and appears to operate by inducing secretion of pro-inflammatory cytokines and chemokines at the injection site, leading to recruitment of innate immune cells^21^.

Systems immunology approaches reveal correlates of vaccine-induced protection, reviewed recently^22^. For example, the yellow fever virus vaccine (YF-17D) induces a strong type I interferon gene signature within a few days of vaccination that correlates with the development of antigen-specific CD8 + T cells and neutralizing antibody (nAb) several weeks later^23,24^. Innate immune gene signatures on d1 post vaccination also correlate with hemagglutination inhibition at day 28 in a seasonal influenza vaccine model^18^. More recently, frequencies of Th1 cells were found to correlate with nAb responses to inactivated influenza vaccines in tonsil organoids ex vivo^25^. Studies such as these will lead to rationales for combining particular adjuvants and vaccine antigens. However, the rational selection of new adjuvants is hampered by a lack of side-by-side comparisons in the same disease models. In addition, the choice of which adjuvant to select going forward is also influenced by strategic decisions, such as freedom to operate, ease and scalability of manufacture, and cost/benefit ratios^26,27^.

To begin to address this, we have screened five different novel combination adjuvants as part of the NIAID Adjuvant Comparison and Characterization (ACC) program^28^. Each adjuvant was administered with a low dose of seasonal influenza vaccines, Flublok® and Fluzone HD® in mice, and immunogenicity and efficacy evaluated. While all of the adjuvants were immunogenic and protective, we noted differences in patterns of inflammatory profiles after prime and boost, Th1/Th2 skewing of the adaptive response, and sex bias of some of the adaptive and reactogenic responses. Side-by-side comparisons such as this will help inform rational adjuvant selection and guide vaccine development for influenza and other infectious diseases.

## Results

### Dose ranging studies establish a low dose for immunization

The vaccines and adjuvants compared in this study are listed in Table 1, with additional details provided in the Methods section.

**Table 1:** Vaccines and adjuvants used in this study

Adjuvant	Composition [PRR]/storage	Dose per mouse (50 μL i.m.)	Source/lot # [Status]
**Alhydroxyquim-II** **(AHQ-II**)	Alhydrogel, imidazoquinoline **[TLR7/8]**/4 °C	90 μg Alhydrogel, 10 μg imidazoquinoline	ViroVax Inc./VV-1-230309 [Approved^a^]
**T-VANT**	Outer membrane vesicles (OMVs) from *Burkholderia pseudomallei* **[TLR4/NLRP3 agonists]**/−80 °C aliquots	1 μg (includes 0.028 EU LPS^b^)	Tulane University/10-1021-618 [Preclinical (NHP)^c^]
**TRAC-478**	INI-2002, INI-4001 (synthetic TLR4, TLR7/8 agonists), nanoparticle emulsion **[TLR4/7/8]**/4 °C	1μg INI-2002 10 μg INI-4001 25μL emulsion	Inimmune/1027-97 [Preclinical (NHP)^d^]
**IVAX-3**^e^	CpG-55.2, MPLA liposome, AddaVax, PBS **[TLR4/9]**/4 °C	7.15 μg CpG-55.2 5.3 μg MPLA 23 μL AddaVax	CpG-55.2: Vaxine^f^/VAX-SPL-2305-05 MPLA: Avanti^g^/699800P-5mg-033 AddaVax®/Invivogen 5805-43-04 [Preclinical (rodents)]
**IVAX-1**^e^	CpG-1018, MPLA liposome, AddaVax, PBS **[TLR4/9]**/4 °C	7.15 μg CpG-1018 5.3 μg MPLA 23 μL AddaVax	CpG-1018: IDT^h^/202138585 MPLA: Avanti^g^ /699800P-5mg-033 AddaVax®/Invivogen 5805-43-04 [Preclinical (rodents)^i^]

2022/2023 Vaccine	Composition	Source	HA conc/dose
**Flublok®**	Quadrivalent, recombinant HA	Sanofi	45 μg each HA/500μL
**Fluzone HD®**	Quadrivalent, split virus	Sanofi	60 μg each HA/700μL
**FluAd®**	Quadrivalent, split virus/MF-59	Seqirus	15 μg each HA/500μL

**“**PRR”, pattern recognition receptor; **“**Status”, the highest level of development of each adjuvant on the path toward licensure.

^a^AHQ-II is approved for use in the Covid19 vaccine, Covaxin® (Bharat Biotech) in India.

^b^T-VANT contains 14 EU/mL LPS in a 0.5 mg/mL protein preparation^29^.

^c^T-VANT has been tested in rodents^29–31^ and NHPs (macaques and baboons; Lisa Morici, pers. comm.).

^d^TRAC-478 emulsion has been tested in rodents^32^, ferrets, pigs, and NHPs, and is scheduled for Phase I clinical trial in 2026 (David Burkhart and Jay Evans, pers. comms.).

^e^Combination adjuvants IVAX-1 and -3 were prepared at UC Irvine by combining AddaVax, CpG and MPLA/liposomes on the day of immunization.

^f^Vaxine Pty Ltd.

^g^Avanti Polar Lipids Inc.

^h^Integrated DNA Technologies Inc.

^i^IVAX-1 has been tested in rodents^33–36^.

**Fig. 1:**
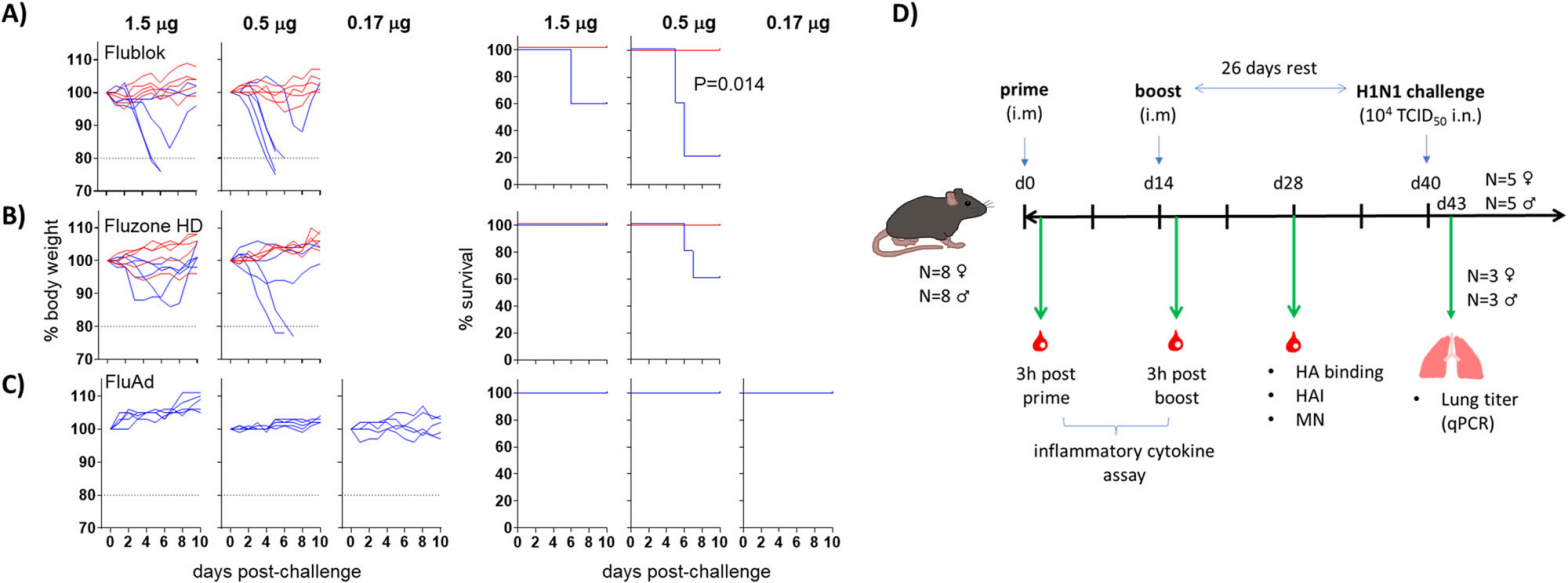
Dose ranging study and subsequent experimental design for side-by-side comparison of novel adjuvants administered with approved seasonal influenza vaccines. **A**–**C** Dose-ranging study. Female C57Bl/6 mice (*N* = 5 per group) were administered Flublok®, Fluzone HD® or FluAd® at 1.5, 0.5 (and 0.17 in the case of FluAd) μg per HA per dose, without or with IVAX-1 adjuvant (blue and red lines, respectively) by the intramuscular (i.m.) route (caudal thigh) on d0 and d14, and challenged on d40 with 50 μL 10^4^ TCID_50_/ml Cal09/PR8 virus (A/California/07/2009 [H1N1] x A/Puerto Rico/8/1934) via the intranasal (i.n.) route. Shown are weights of individual mice expressed as a % of the original body weight (left) and corresponding % survival plots (right). Hashed line, 80% cutoff below which animals were euthanized. **D** Timeline for adjuvant comparison studies. Male and female C57Bl/6 mice (*N* = 8 per group) were administered 0.5 μg (low-dose) Flublok or Fluzone HD with or without the adjuvants listed in Table 1. Control mice received FluAd (also 0.5ug per HA per dose) or PBS. All formulations were administered via the i.m. route and boosted on d14. Bleeds for inflammatory cytokine assays were taken 3 h post-prime and post-boost, and on d28 for serology. Mice were challenged on d40 using Cal09/PR8 via the i.n. route. Three mice from each group were euthanized on d3 post-challenge for lung titer determination by qPCR, and the remaining 5 mice monitored until the endpoint (10–12 days post-challenge or >20% weight loss). Note: T cell recall assays were performed on separate cohorts of mice.

To reveal the effects of each adjuvant, vaccine dose ranging studies were first conducted with a single adjuvant. The aim was to establish a dose of vaccine antigen that failed to protect, but which became efficacious when adjuvanted. For this, two doses of Flublok® and Fluzone HD® were tested in vivo (1.5 μg and 0.5 μg each HA per 50 μL dose) and administered to separate cohorts of female C57Bl/6 mice via the intramuscular (i.m.) route, with or without an adjuvant (IVAX-1). IVAX-1 was selected as a representative strong combination adjuvant for dose-ranging. We reasoned that the low dose defined by a strong adjuvant would best reveal any differences between the panel of adjuvants tested. Animals were boosted with the same dose and formulation on d14 and challenged on d28 with 50 μL H1N1 Cal09/PR8 at 10^4^ TCID_50_/mL via the intranasal (i.n.) route. Sixty percent of mice receiving a 1.5 μg dose of Flublok® without adjuvant survived, while the 0.5 μg dose elicited 20% survival, both with some morbidity i.e., initial weight loss with recovery (Fig. 1A). When administered in IVAX-1, 100% of mice survived without morbidity at both doses, with the lower dose reaching significance when compared to the non-adjuvanted vaccine. Similarly, mice receiving the 1.5 μg dose of Fluzone HD® without adjuvant elicited 100% survival, while 0.5 μg elicited 60% survival, both with some morbidity (Fig. 1B). When administered in IVAX-1, survival was enhanced to 100% at both doses, with no morbidity noted. FluAd® was used as a positive control and tested for efficacy at the same doses as the seasonal vaccines (1.5 and 0.5 μg/dose, with an additional dose of 0.17 μg/dose) for comparison. To preserve the stability of the emulsion, dilutions of FluAd® were performed in a 1:1 mixture of AddaVax® in water (equivalent to 1:1 MF59® and antigen solution as used in FluAd®). All three doses of FluAd® elicited 100% survival without morbidity (Fig. 1C). On the basis of these data, 0.5 μg per HA for Flublok®, Fluzone HD® and FluAd®, was the low-dose chosen for subsequent adjuvant screens (Fig. 1D). The same low-dose regimen was used in males and females to allow any sex bias in the response to be revealed.

### Innate immune responses varied between different adjuvants

**Fig. 2:**
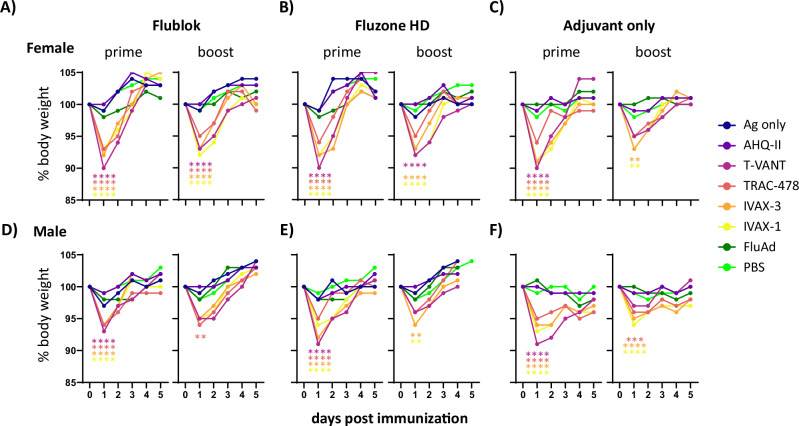
Transient weight loss following prime and boost with adjuvanted seasonal vaccines. **A−C** Female and **D−F** male C57Bl/6 mice were administered low-dose Flublok or Fluzone HD in different adjuvants (Table 1) as listed in the legend, with control mice receiving vaccine only (“Ag only”), FluAd (also 0.5ug per HA per dose) or PBS. Additional cohorts received Adjuvant only. Formulations were administered via the intramuscular route (caudal thigh) and animals monitored daily. Shown are body weights (expressed as a % of the original weight) with each spot representing the median group weight (*N* = 8 per group). Asterisks correspond to significant differences at day 1 (peak weight loss) between vaccine groups with the corresponding Ag only control group, or with PBS in the case of the Adjuvant only cohort (ordinary one-way ANOVA; ****P < 0.0001; ***P < 0.001; **P < 0.01; others are non-significant).

To assess whether any of the formulations caused adverse reactions, mice were monitored daily for changes in appearance, behavior, and body weight for ~1 week after prime and boost. No local reactions were observed at the site of injection. We observed transient weight loss of up to ~10% following administration of T-VANT, TRAC-478, IVAX-1, and IVAX-3 compared to the antigen-only control group (Fig. 2A, B), which we attribute to a temporary reduction in food intake. Weight loss was maximal one day after injection and was steadily regained over the following 2–4 days. In contrast, AHQ-II, FluAd or PBS did not cause weight loss relative to antigen (Ag)-only controls.

The same weight loss patterns were also seen in animals receiving adjuvant alone (Fig. 2C), but not in animals receiving vaccine Ag alone, indicating the effect is mediated by the adjuvants. Similar levels of weight loss were seen after the boost on d14, indicating that none of the adjuvants caused enhanced reactogenicity upon secondary immunization. Weight loss was slightly less in males than females, although this failed to reach significance (not shown). In this context, males are significantly heavier than age-matched females (i.e., 18.3 g ± 1.1 g for females vs. 24.5 g ± 1.5 g for males at prime, and 19.7 g ± 1.3 g for females vs. at 26.0 g ± 1.8 g at boost) which may, along with differences in immune responses by males and females, contribute to sex-specific differences in transient weight loss. We also observed piloerection for the first 1 or 2 days in most males receiving T-VANT after prime and boost; otherwise, we saw no other changes in behavior or appearance after prime or boost immunizations in either sex.

We also quantified inflammatory cytokines in the blood 3 h after prime and boost using a 13-plex bead-based cytokine array (LEGENDplex). The breadth and quantity of different cytokines induced varied widely between adjuvants, with T-VANT inducing the broadest profile (*N* = 11 or 12 according to group) relative to the Ag only control groups, and AHQ-II inducing the narrowest profile (*N* = 0) (Table 2).

**Table 2:** Inflammatory cytokine profiles in the blood 3 h after prime and boost

FEMALES	Vaccine	Adjuvant	IL-23	IL-1α	IFN-γ	TNF-α	MCP-1/CCL2	IL-12 p70	IL-1β	IL-10	IL-6	IL-27	IL-17A	IFN-β	GM-CSF	Total
**Post-prime**	**Flublok**	AHQ-II	ns	ns	ns	ns	ns	ns	ns	ns	ns	ns	ns	ns	ns	0
		T-VANT	*	ns	*	**	**	*	ns	*	**	*	*	*	*	11
		TRAC-478	ns	ns	ns	ns	***	ns	ns	ns	**	ns	ns	ns	ns	2
		IVAX-3	ns	ns	ns	***	***	ns	ns	ns	**	ns	ns	ns	ns	3
		IVAX-1	ns	ns	ns	***	***	ns	ns	ns	**	ns	ns	ns	ns	3
	**Fluzone HD**	AHQ-II	ns	ns	ns	ns	ns	ns	ns	ns	ns	ns	ns	ns	ns	0
		T-VANT	*	ns	*	**	***	*	ns	*	**	*	*	*	*	11
		TRAC-478	ns	ns	ns	ns	ns	ns	ns	ns	**	ns	ns	ns	ns	1
		IVAX-3	ns	ns	ns	***	***	ns	ns	ns	**	ns	ns	ns	*	4
		IVAX-1	ns	ns	ns	***	***	ns	ns	ns	**	ns	ns	*	ns	4
	**Adj only**	AHQ-II	ns	ns	ns	ns	ns	ns	ns	ns	ns	ns	ns	ns	ns	0
		T-VANT	*	ns	**	**	**	**	ns	*	**	**	*	*	**	11
		TRAC-478	ns	ns	ns	ns	**	ns	ns	ns	**	ns	ns	ns	ns	2
		IVAX-3	ns	ns	ns	*	**	ns	ns	ns	**	ns	ns	ns	ns	3
		IVAX-1	ns	ns	ns	*	**	ns	ns	ns	**	ns	ns	ns	ns	3
	**FluAd**		ns	ns	ns	ns	ns	ns	ns	ns	**	ns	ns	ns	ns	1
**Post-boost**	**Flublok**	AHQ-II	ns	ns	ns	ns	ns	ns	ns	ns	ns	ns	ns	ns	ns	0
		T-VANT	ns	ns	*	ns	**	ns	ns	ns	**	ns	ns	**	ns	4
		TRAC-478	ns	ns	***	***	**	**	**	ns	**	**	**	**	**	10
		IVAX-3	ns	*	***	***	**	ns	ns	ns	**	ns	ns	ns	ns	5
		IVAX-1	ns	**	***	***	**	ns	ns	ns	**	ns	ns	ns	ns	5
	**Fluzone HD**	AHQ-II	ns	ns	ns	ns	ns	ns	ns	ns	ns	ns	ns	ns	ns	0
		T-VANT	ns	ns	**	ns	**	ns	ns	ns	**	ns	ns	**	ns	4
		TRAC-478	**	ns	***	**	**	ns	**	ns	**	*	**	**	**	10
		IVAX-3	ns	ns	***	**	**	ns	ns	ns	**	ns	ns	ns	ns	4
		IVAX-1	ns	ns	**	**	**	ns	ns	ns	**	ns	ns	ns	ns	4
	**Adj only**	AHQ-II	ns	ns	ns	ns	ns	ns	ns	ns	ns	ns	ns	ns	ns	0
		T-VANT	**	ns	ns	ns	**	ns	ns	ns	*	ns	ns	*	ns	4
		TRAC-478	**	ns	***	*	**	**	**	ns	*	**	**	**	**	11
		IVAX-3	ns	ns	ns	ns	**	ns	ns	ns	**	ns	ns	ns	ns	2
		IVAX-1	ns	ns	ns	ns	**	ns	ns	ns	**	ns	ns	ns	ns	2
	**FluAd**		ns	ns	ns	ns	ns	ns	ns	ns	*	ns	ns	ns	ns	0

MALES	Vaccine	Adjuvant	IL-23	IL-1α	IFN-γ	TNF-α	MCP-1/CCL2	IL-12 p70	IL-1β	IL-10	IL-6	IL-27	IL-17A	IFN-β	GM-CSF	Total
**Post-prime**	**Flublok**	AHQ-II	ns	ns	ns	ns	ns	ns	ns	ns	ns	ns	ns	ns	ns	0
		T-VANT	*	ns	**	**	**	ns	*	**	**	**	*	*	ns	10
		TRAC-478	ns	ns	ns	ns	ns	ns	ns	ns	*	ns	ns	ns	ns	1
		IVAX-3	ns	ns	ns	ns	ns	ns	ns	ns	**	ns	ns	ns	ns	1
		IVAX-1	ns	ns	ns	ns	ns	ns	ns	ns	**	ns	ns	ns	ns	1
	**Fluzone HD**	AHQ-II	ns	ns	ns	ns	ns	ns	ns	ns	ns	ns	ns	ns	ns	0
		T-VANT	ns	**	**	**	**	*	ns	**	***	**	ns	*	ns	9
		TRAC-478	ns	ns	ns	ns	ns	ns	ns	ns	**	ns	ns	ns	ns	1
		IVAX-3	ns	ns	ns	ns	*	ns	ns	ns	*	ns	ns	ns	ns	2
		IVAX-1	ns	ns	ns	ns	**	ns	ns	ns	***	ns	ns	ns	ns	2
	**Adj only**	AHQ-II	ns	ns	ns	ns	ns	ns	ns	ns	ns	ns	ns	ns	ns	0
		T-VANT	*	*	**	**	**	**	ns	**	***	**	**	**	**	12
		TRAC-478	ns	ns	ns	ns	ns	ns	ns	ns	***	ns	ns	ns	ns	1
		IVAX-3	ns	ns	ns	ns	**	ns	ns	ns	***	ns	ns	ns	ns	2
		IVAX-1	ns	ns	ns	ns	**	ns	ns	ns	***	ns	ns	ns	ns	2
	**FluAd**		ns	ns	ns	ns	ns	ns	ns	ns	ns	ns	ns	ns	ns	0
**Post-boost**	**Flublok**	AHQ-II	ns	ns	ns	ns	ns	ns	ns	ns	ns	ns	ns	ns	ns	0
		T-VANT	**	ns	**	ns	**	ns	*	**	***	**	ns	**	ns	8
		TRAC-478	**	ns	***	ns	*	ns	*	**	***	**	*	**	ns	9
		IVAX-3	ns	ns	***	ns	*	ns	ns	ns	***	ns	ns	ns	ns	3
		IVAX-1	ns	ns	ns	ns	*	ns	ns	*	***	ns	ns	ns	ns	3
	**Fluzone HD**	AHQ-II	ns	ns	ns	ns	ns	ns	ns	ns	ns	ns	ns	ns	ns	0
		T-VANT	ns	ns	***	*	*	ns	ns	*	*	ns	ns	**	ns	6
		TRAC-478	**	ns	***	**	*	ns	ns	**	*	**	ns	**	**	9
		IVAX-3	ns	ns	**	ns	**	ns	ns	ns	**	ns	ns	ns	ns	3
		IVAX-1	ns	ns	**	ns	*	ns	ns	ns	**	ns	ns	ns	ns	2
	**Adj. only**	AHQ-II	ns	ns	ns	ns	ns	ns	ns	ns	ns	ns	ns	ns	ns	0
		T-VANT	ns	ns	ns	ns	*	ns	ns	ns	**	ns	ns	ns	ns	2
		TRAC-478	ns	ns	ns	ns	*	ns	ns	ns	**	ns	ns	ns	ns	2
		IVAX-3	ns	ns	ns	ns	*	ns	ns	ns	ns	ns	ns	ns	ns	1
		IVAX-1	ns	ns	ns	ns	*	ns	ns	ns	**	ns	ns	ns	ns	2
	**FluAd**		ns	ns	ns	ns	ns	ns	ns	ns	ns	ns	ns	ns	ns	0

Female and male C57Bl/6 mice were administered a low-dose Flublok or Fluzone HD with or without different adjuvants, or adjuvant only as described in Fig. 1. Cytokines were quantified using a multiplex bead array (LEGENDplex) and expressed as pg/mL. Statistical significance was determined by comparing adjuvanted vaccine groups against the corresponding antigen only control groups, or PBS in the case of adjuvant only and FluAd groups, using a Mann–Whitney *U* test with Benjamini–Hochberg correction for false discovery. ***P < 0.001; **P < 0.01; *P < 0.05.

*ns* non-significant, *Adj* adjuvant.

**Fig. 3:**
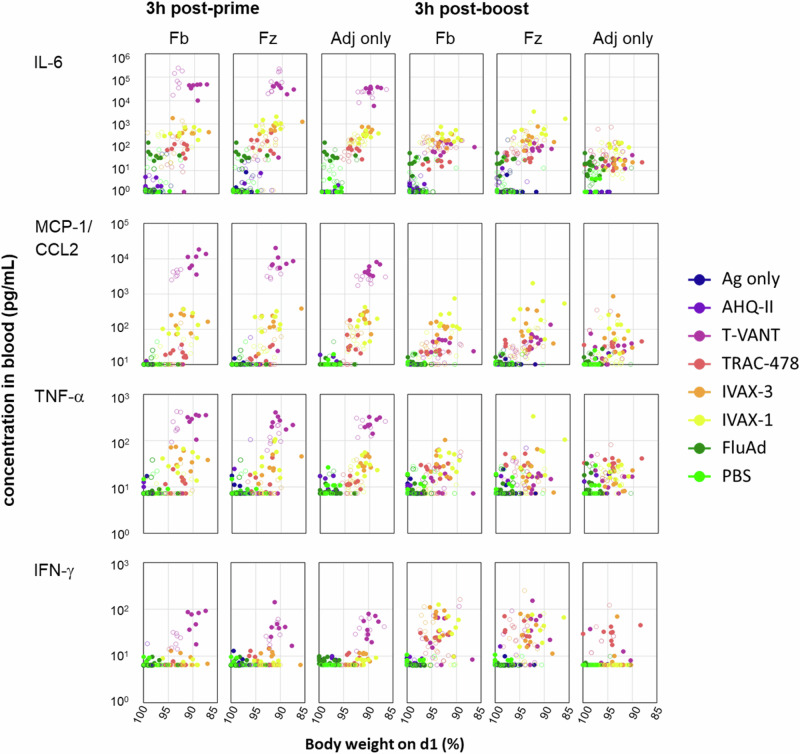
Relationship between inflammatory cytokines and transient weight loss. Female and male mice were administered low-dose seasonal vaccines in different adjuvants (Table 1). Concentration of cytokines in blood (pg/mL) at 3 h post-prime and post-boost are shown plotted against the corresponding weight on d1 post-prime or boost (data from Fig. 2). Each dot represents a single mouse: females, solid symbols; males, open symbols. Data for all inflammatory cytokines are provided in Supplementary Fig. S1. Fb Flublok, Fz Fluzone HD, Adj adjuvant, Ag antigen.

Cytokines IL-6, MCP-1/CCL2 and TNF-α were consistently elevated in mice receiving T-VANT, TRAC-478, IVAX-3 and IVAX-1, which we noted are the same adjuvants associated with transient weight loss after prime and boost, as shown in Fig. 2. After priming, T-VANT induced the highest concentrations of these cytokines relative to the Ag only control group. TRAC-478, IVAX-3 and IVAX-1 induced intermediate increases in these cytokines, while AHQ-II induced the lowest concentrations. Scatter plots between cytokine release against transient weight loss data revealed a trend in which higher inflammatory cytokine levels were associated with increased weight loss, although correlation coefficients failed to reach significance (Fig. 3 and Supplementary Fig. S1).

In this context, IL-6 was the only cytokine in the 13-plex panel that was induced by FluAd®. FluAd® induced <5% transient weight loss after prime and boost, suggesting this reactogenicity is unlikely to be associated with IL-6 release. Also shown in Fig. 3 is IFN-γ, which is representative of the several cytokines that are produced by T-VANT after a prime only; the others comprise IL-12p70, IL-27, IL-17A, IFN-β and GM-CSF (Supplementary Fig. S1). Other adjuvants induced broadly similar profiles after prime and boost, with the exception of IFN-γ which was typically elevated after the boost, consistent with an antigen-specific recall response in vivo. Similar patterns of cytokine release and transient weight loss were caused by the adjuvants alone, although in some cases the quantity of cytokine released was lower than when combined with seasonal vaccines. These data indicate the release of inflammatory cytokines is predominantly adjuvant-mediated, but may be further elevated by addition of particular antigens.

### Magnitude and subtype of IgG response is influenced by adjuvant

**Fig. 4:**
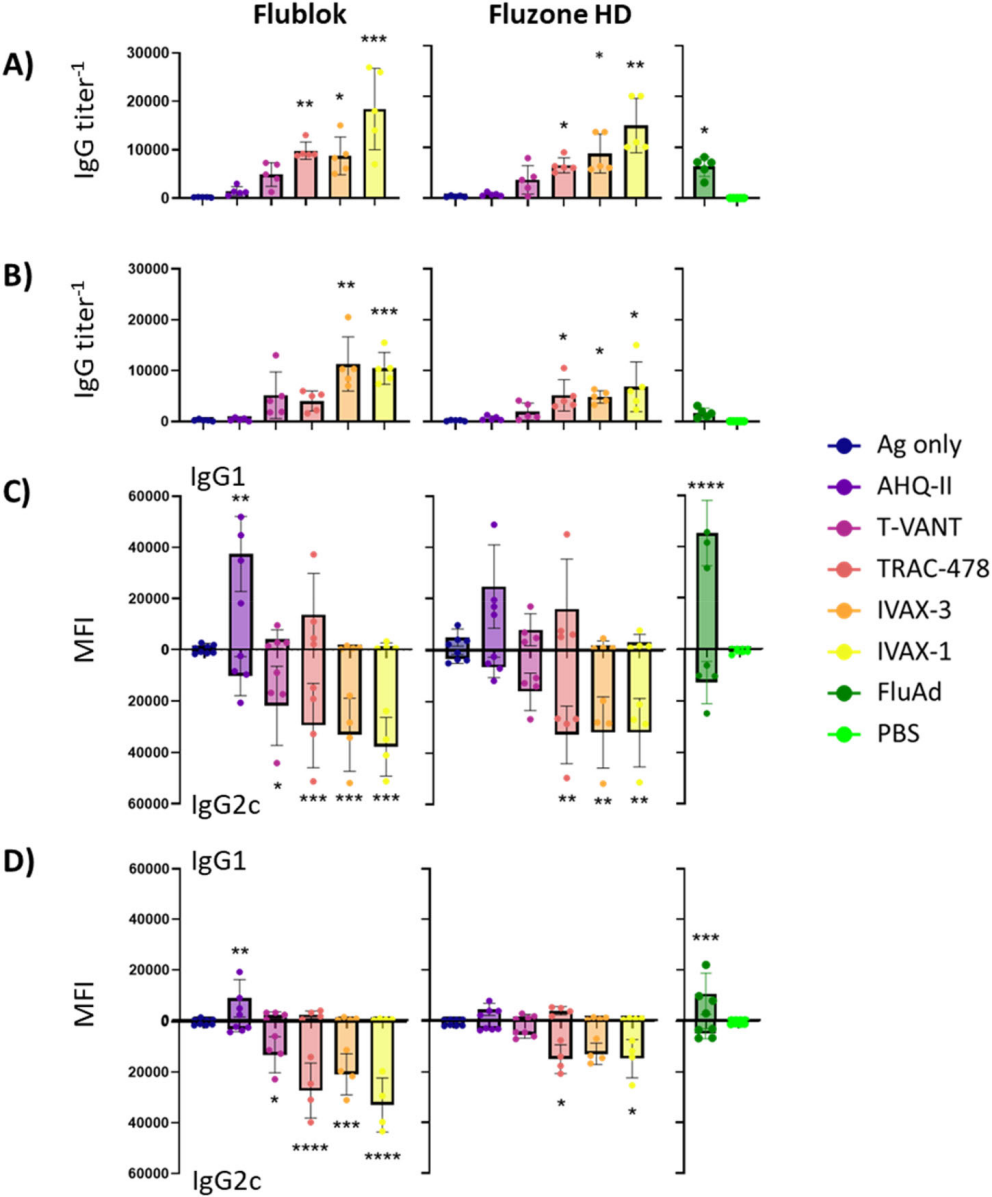
H1-specific IgG responses. Mice were administered low-dose seasonal vaccines in different adjuvants (Table 1) as listed in the legend, and serological assays performed on plasma collected on d28 (2 weeks post-boost). **A**, **B** ELISA midpoint titers of females and males (*N* = 5 per group), respectively. **C**, **D** normalized protein microarray IgG1 and IgG2c signals to the 4 vaccine antigens in females and males (*N* = 8 per group), respectively. Similar plots of cross-reactivity for variants not included in the vaccine are shown in Supplementary Fig. S2. MFI, mean fluorescence intensity. Statistical significance was determined by comparing vaccine groups against the Ag only control group, or PBS in the case of FluAd, using ordinary one-way ANOVA. ****P < 0.0001; ***P < 0.001; **P < 0.01; *P < 0.05; others are non-significant.

Plasma samples collected on d28 (2 weeks post-boost) were assayed by ELISA and protein microarray for magnitude and breadth of response. By ELISA, all of the adjuvants induced IgG titers above those induced by Ag alone, which were typically higher in females than males (Fig. 4A, B, respectively). The IgG1 and IgG2c signals were also quantified on protein microarrays. Overall, signals from females were higher than males (Fig. 4C, D, respectively), consistent with the ELISA data. Quantitatively, the highest signals were achieved using IVAX-1, IVAX-3 and TRAC-478, also consistent with the ELISA data. Qualitatively, IVAX-1 and -3 produced IgG2c (Th1) polarized responses in both females and males, while AHQ-II was polarized toward IgG1 (Th2). T-VANT and TRAC-478 engendered a more balanced response in females but a more IgG2c-polarized response in males. Homosubtypic cross-reactivity for non-vaccine variants of H1 (*N* = 26), H3 (*N* = 29) and B HAs (*N* = 16), and for heterosubtypic HAs (*N* = 81) was lower than expected based on previous array studies of adjuvanted HAs^33,34^ (Supplementary Fig. S2A–E). We attribute this to the low concentration of antigen (0.5 μg/dose) used for immunization, which is 1/10 of the antigen dose we typically used for previous studies^33,34^.

The arrays also revealed robust anti-nucleoprotein (NP) responses (*N* = 5 variants) from Fluzone HD®, consistent with its inactivated/split vaccine manufacturing process, and which followed the same IgG1/IgG2c pattern seen for the other vaccine Ags. Flublok® did not elicit antibodies to NP, which is consistent with a recombinant HA-based vaccine lacking other virus components (Supplementary Fig. S2F).

### Neutralizing antibody (nAb) titers are adjuvant dependent

**Fig. 5:**
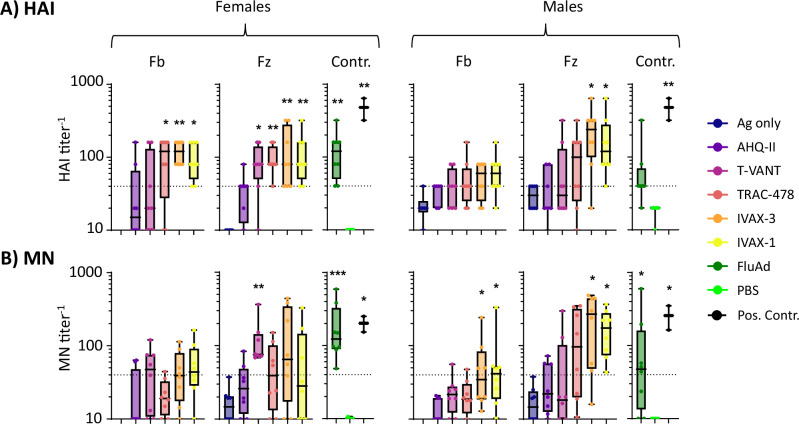
Virus neutralization. Mice were administered low-dose seasonal vaccines in different adjuvants (Table 1) as listed in the legend. Plasma collected on d28 (2 weeks post-boost) were tested for homosubtypic cross-neutralization of H1N1 (from A/California/07/2009) by **A** hemagglutination inhibition (HAI) and **B** microneutralization (MN) assays. Two hyperimmune plasma samples were also included as positive controls (black symbols). Hashed horizontal line, titer of 1:40. Statistical significance determined by comparing vaccine groups against no Adj control group, or PBS in the case of FluAd, using a one-way ANOVA (Kruskal–Wallis test);  ***P < 0.001; **P < 0.01; *P < 0.05; others are non-significant.

Neutralizing antibody (nAb) titer is an established correlate of protection for seasonal influenza vaccines. Thus, plasma collected on d28 from mice immunized as per Fig. 1D were assayed for neutralization of Cal09/PR8 virus in vitro, which is the same variant used for challenge studies, described below (Fig. 5). Overall, the nAb response was heterogeneous, with some or all of the mice in each of the adjuvanted groups reaching a titer of 1:40, which is typically used as the cut-off to indicate protection in human samples^37^. None of the mice receiving non-adjuvanted seasonal vaccines had titers above the 1:40 cutoff. In addition, males generated slightly lower titers to corresponding female groups (consistent with ELISA and array data), although the differences failed to reach significance.

### T cell responses show Th1 vs Th2 skewing that correspond to IgG subtypes

To assess the effect of adjuvants on immunogenicity at the T cell level, antigen-specific recall assays were performed with spleen cells 8-9 days after prime and boost using IFN-γ and IL-4 ELISpots and by multiplex cytokine assay of culture supernatants (LEGENDplex). Assays were performed using Flublok® (recombinant HA-based) to better assess the effects of each adjuvant in the absence of other potential adjuvant effects typically associated with inactivated/split vaccines, such as genomic RNA^38^. In the assays, recombinant H1, H3 and B HA that were matched to the vaccine strains were used for restimulation in vitro; H5 antigen was also included in the assay to test for any heterosubtypic T cell cross-reactivity. Initial studies using the low dose vaccine (0.5 μg/HA) failed to engender detectable T cell responses (data not shown). In contrast, a 10-fold higher antigen dose (5 μg/HA) generated measurable responses in these assays.

**Fig. 6:**
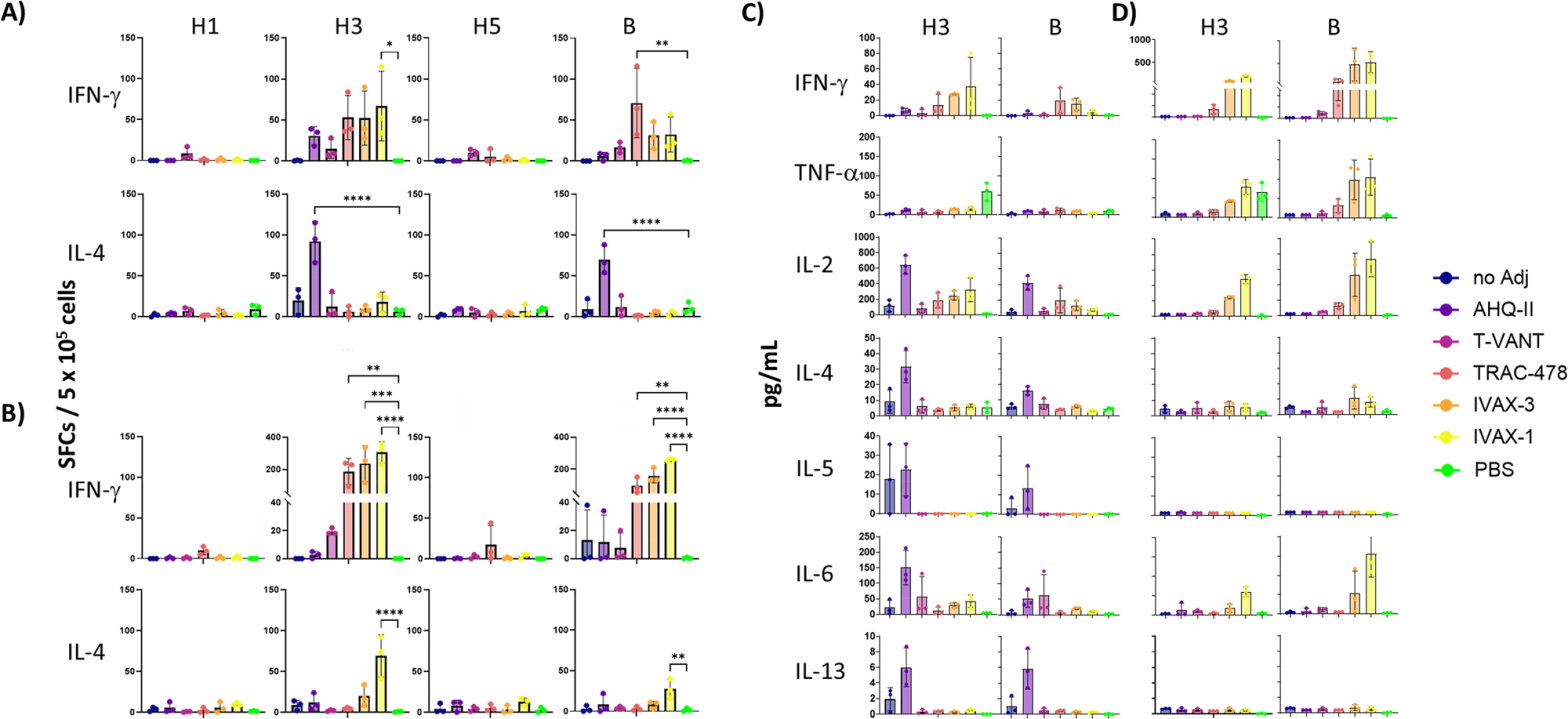
T cell recall assay. Female C57Bl/6 mice (*N* = 3 per group) were administered Flublok® (5 μg per HA per dose) with or without adjuvant as indicated in the legend. Control mice received PBS only. Formulations were administered i.m., and spleens harvested for IFN-γ and IL-4 ELISpots on **A** 8 days post-prime, and **B** d23, i.e., 9 days post-boost on d14. Culture supernatants from the ELISpot in panels A) and B) were assayed for secreted cytokines using a cytokine multiplex assay (LEGENDplex) on **C** post-prime, and **D** post-boost time points. Concanavalin A was used as a stimulation control (not shown). Shown are responses after restimulation with 10 μg/mL HA antigen (H1, H3, H5 from influenza A, or from influenza B) in the recall assay. Statistical significance determined by comparing vaccine groups against Ag only control group using an ordinary one-way ANOVA; ****P < 0.0001; ***P < 0.001; **P < 0.01; *P < 0.05; others are non-significant); SFC, spot-forming cells.

Numbers of IFN-γ and IL-4 spot forming cells (SFCs) in splenocytes after a prime or boost are shown in Fig. 6A, B, respectively. Robust recall responses in vitro were observed against H3 and B HAs, while H1 and H5 failed to restimulate T cells. The weak H1 response is consistent with previous reports of the relatively weak immunogenicity of H1 relative to other HA subtypes in C57Bl/6 mice (see Figs. 2 and 5 in ref. ^33^), which we presume also accounts for the lack of T cell cross-reactivity for H5. Of the positive responses, splenocytes from mice administered Flublok/AHQ-II mounted a robust IL-4 response and a weak IFN-γ response, consistent with the observed IgG1 (Th2) polarization of the antibody response (Fig. 4C, D). The number of IL-4 SFCs was strongest after the prime, although there were undetectable numbers of IL-4 SFCs after the boost. In contrast, mice administered Flublok® in T-VANT, TRAC-478, IVAX-3 or IVAX-1 mounted robust IFN-γ responses and weak IL-4 responses, consistent with the IgG2c (Th1) skewing of the serological response (Fig. 4C, D). While the IFN-γ response was seen after both prime and boost, it was considerably elevated after the boost. Moreover, we observed significant levels of IL-4 after the boost in mice receiving Flublok® in IVAX-1, indicating that boosting in IVAX-1 drives the response to become more Th1/Th2 balanced. Animals receiving Flublok® without adjuvant mounted a weak response that was associated with IL-4 only, suggesting the default response to recombinant HA antigen (expressed in insect cells) in the absence of added TLR agonists is Th2-biased. Importantly, cytokine profiling from the supernatants of restimulated splenocytes using a 13-plex cytokine bead array (LEGENDplex) after prime and boost (Fig. 6C, D, respectively) confirmed the Th1:Th2 polarizations seen by ELISpot. Thus, recall of splenocytes with H3 and B HAs from mice administered Flublok® alone or Flublok®/AHQ-II stimulated release of Th2 cytokines, IL-4, -5, -6 and -13 after the prime, while TRAC-478, IVAX-3 and -1 stimulated release of Th1 cytokines, IFN-γ, TNF-α and IL-2, which were considerably higher after the boost.

### All adjuvants are efficacious against H1N1 challenge with varying morbidities

Mice primed and boosted with low-dose seasonal vaccines as per Fig. 1, were challenged with 10^4^ TCID_50_/mL Cal09/PR8 (H1N1) virus on d40, i.e., 26 days after the boost. Positive control mice had received FluAd, while negative control mice received PBS. The H1 in Cal09/PR8 is mismatched at 29/566 amino acids relative to the 2022/2023 vaccines used to immunize, of which 20 residues are located in the HA1 fragment that contains the head and part of the stalk. Similarly, the N1 from Cal09/PR8 (H1N1) is 30/469 amino acids mismatched with the vaccine strain. A heterologous challenge was used to help discriminate between adjuvants better than fully-matched antigens.

**Fig. 7:**
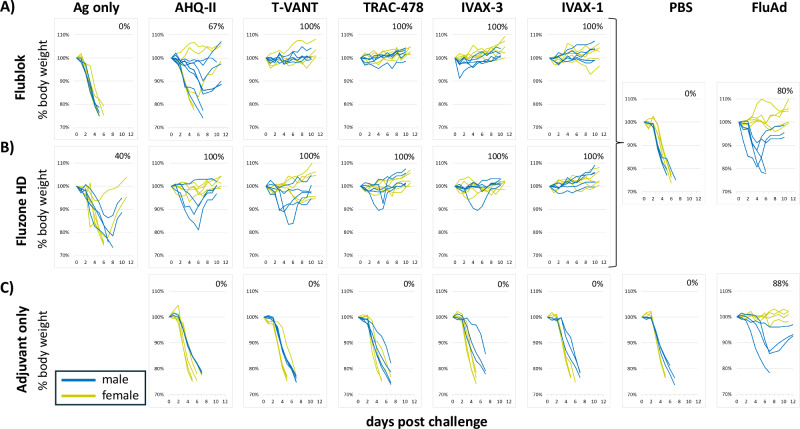
Challenge study. Female and male C57Bl/6 mice (*N* = 8 per group) were immunized and boosted with **A** Flublok, **B** Fluzone HD, or **C** Adjuvant only, and challenged 26 days after the boost with 10^4^ TCID_50_/mL of Cal09/PR8 H1N1 virus, as described in Fig. 1. Three animals from each group were sacrificed on d3 post-challenge for viral lung titer determination by qPCR (see Fig. 8). Shown are body weights of the remaining individual mice expressed as a percentage of their pre-challenge body weight. The percentages in each panel are the percent survival of the combined female and male groups for each vaccine and adjuvant combination.

Figure 7A, B shows changes in body weights of individual mice after challenge. Mice losing <5% of their body weight at any time during the experiment were considered protected without morbidity, whereas mice that lost between 5-20% body weight and regained the weight were considered protected with morbidity. Mortality (i.e., not protected) was defined as animals that lost >20% body weight and were humanely euthanized. Mice receiving non-adjuvanted Flublok® were not protected, while the same dose of non-adjuvanted Fluzone HD® engendered 40% survival, consistent with the dose-ranging study (Fig. 1A, B). It is possible the slightly better performance of non-adjuvanted Fluzone HD® is due to inherent adjuvanticity of inactivated/split vaccines prepared from whole virus^38^ compared to recombinant HA-based vaccines. All of the mice receiving adjuvanted vaccines were protected to varying degrees; mice receiving Flublok® adjuvanted in T-VANT, TRAC-478, IVAX-3 and IVAX-1 showed 100% survival and no morbidity, while those receiving Flublok® adjuvanted in AHQ-II showed <100% survival and some morbidity (weight loss that was regained) in the majority of animals. Mice receiving Fluzone HD® adjuvanted in AHQ-II, T-VANT, TRAC-478, IVAX-3 and IVAX-1 showed 100% survival with morbidity (mostly in males) in at least one animal in all groups other than IVAX-1. Mice receiving Adjuvant only (Fig. 7C) showed 0% survival, confirming the protection from adjuvanted Flublok® or Fluzone HD® is antigen-dependent. In the control groups, mice receiving PBS were not protected, while survival in the FluAd® positive control groups was less than 100%, with greater morbidity in males than females.

**Fig. 8:**
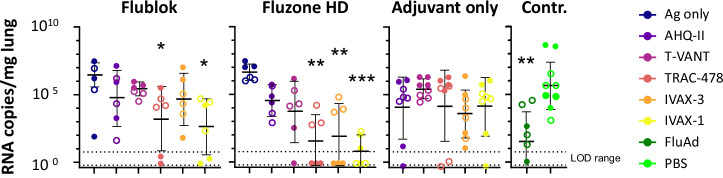
Viral RNA copies in lungs 3 days post-challenge. Female and male C57Bl/6 mice (*N* = 8 per group) were immunized and challenged with H1N1 as described in Fig. 1. Lungs were harvested on d3 post-challenge from 3 mice per group for qPCR, and the remaining mice were monitored for weight changes (Fig. 7). Data from females (solid symbols) and males (open symbols) are pooled to increase N. Statistical significance was determined by comparing vaccine groups against corresponding Ag only control groups, or PBS in the case of Adjuvant only and FluAd groups, using a one-way ANOVA (Kruskal-Wallis test). LOD, limit of detection.

Lung viral load on d3 post challenge determined by qPCR are shown in Fig. 8. In the control groups, there was a ~3–4-log_10_ difference in median load between mice administered PBS and FluAd. Mice receiving seasonal vaccines showed varying reductions of lung loads, although the span in each group was broad with some of the groups reaching significance compared to the PBS control.

### Meta-analyses reveal potential correlates of protection

The immunoprofiling data generated for individual mice in this study allowed for correlations between different immunogenicity metrics and disease outcomes. We showed above (Fig. 3) there is a relationship between inflammatory cytokines IL-6, MCP-1/CCL2, and TNF-α in the blood after prime and transient weight loss. Two additional analyses are shown below.

**Fig. 9:**
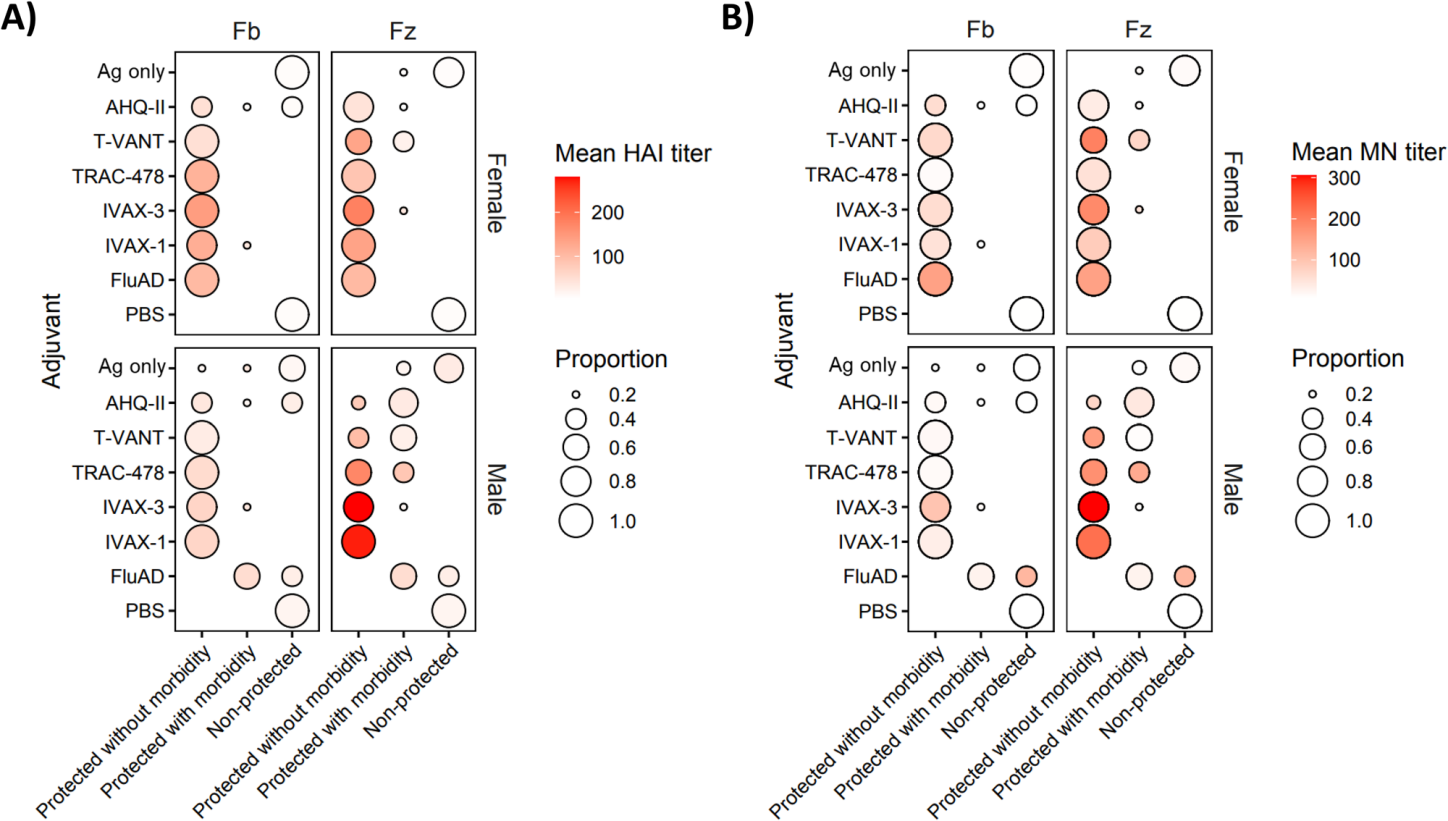
Correlation between neutralizing Ab titers and efficacy. Shown are bubble plots of nAb titers on d28 determined by (**A**) HAI, and (**B**) MN assays (from Fig. 5), and corresponding efficacy. Efficacy is defined by the lowest weight seen at any time after H1N1 challenge (from Fig. 7), where <5% weight loss = protected without morbidity, 5-20% weight loss = protected with morbidity, and >20% weight loss = non-protected (animals were euthanized). Size of bubbles indicate the proportion of animals in each of the 4 conditions for each adjuvant. Fb Flublok, Fz Fluzone HD, HAI hemagglutination inhibition, MN microneutralization.

Titers of nAbs induced by seasonal influenza vaccines are traditionally considered the main correlate of protection (COP) against infection^37^. To evaluate the correlation between nAb titers and efficacy, we first plotted hemagglutination inhibition (HAI) and microneutralization (MN) assay titers against each other to assess concordance of the two types of assay. While HAI and MN titers show a positive association, the R^2^ value is only 0.46 and 0.66 for Flublok® and Fluzone HD® respectively (Supplemental Fig. S3), reflecting partial overlap of antibody functionalities measured by the two assays. We then produced plots of HAI and MN titers on d28 (data from Fig. 5) against corresponding outcome after challenge (data from Fig. 7) (Fig. 9A, B). As expected, reduced morbidity and mortality was associated with higher nAb titers. Scatter plots of maximal weight loss against nAb titers (Supplemental Fig. S4) revealed all the females and most of the males with nAb titer 1:40 and above were protected without morbidity. Interestingly, a significant proportion of animals with titers at or below 1:40 that were also protected, which was seen predominantly with T-VANT and TRAC-478 (Supplemental Fig. S5). While a cutoff for protection is likely to be different for mice than for humans, the large number of animals with low nAb titers that were protected suggests protection can be mediated by non-neutralizing antibodies (possibly via complement fixation or antibody-dependent cell-mediated cytotoxicity), and/or antibody-independent mechanisms, such as killing of infected cells by CD8 T cells.

We also assessed the correlation between post-vaccination nAb titers on d28 in plasma and the corresponding virus lung titers on d3 post challenge for each animal (Supplementary Fig. S6). While there was a similar trend for higher lung titers to be associated with lower nAb titers, as expected, the correlation coefficients were low. Lung or nasal washes were not collected prior to challenge in this study, but nAbs titers in such samples might provide improved correlations with lung titers post-challenge compared to nAb titers in plasma.

**Fig. 10:**
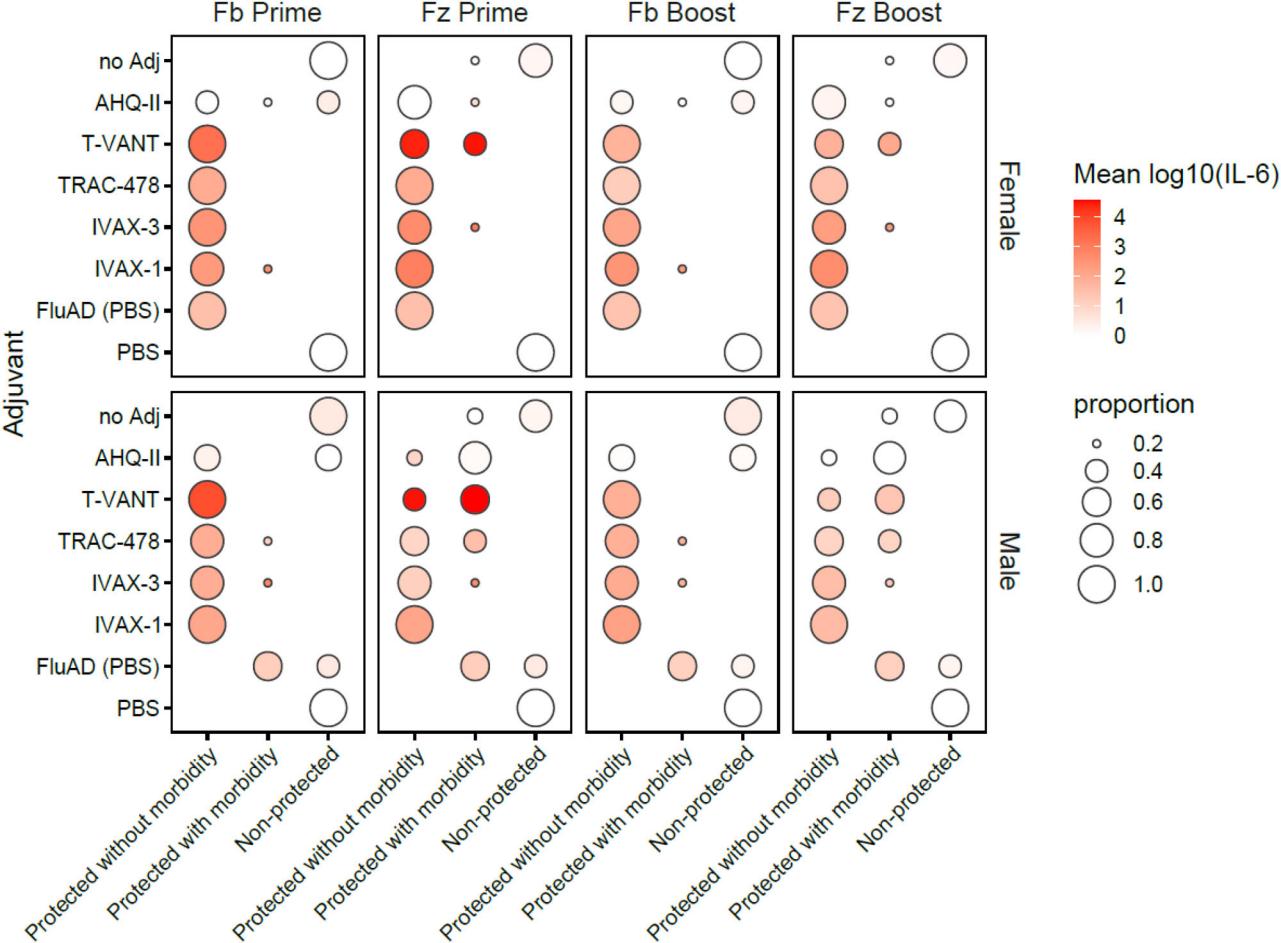
Correlation between IL-6 in the blood 3 h after vaccination and efficacy. Shown are bubble plots of IL-6 in the blood at 3 h after prime and boost, and corresponding efficacy. IL-6 is expressed as log fold-over Ag only controls, except for FluAd which was expressed as log fold-over PBS control (“FluAd (PBS)”). Efficacy is defined by the lowest weight seen at any time after H1N1 challenge (from Fig. 7), where <5% weight loss = protected without morbidity, 5–20% weight loss = protected with morbidity, and >20% weight loss = non-protected (animals were euthanized). Size of bubbles indicate the proportion of animals in each of the 4 conditions for each adjuvant.Fb Flublok, Fz Fluzone HD, FC fold-over control. Similar plots for other cytokines are shown in Supplementary Fig. S7.

To identify other potential COPs, we compared inflammatory cytokine release 3 h after prime or boost with outcomes after challenge. Adjuvants that conferred the best protection when administered with seasonal influenza vaccines induced several inflammatory cytokines (Table 2). Of note, FluAd, which was efficacious, induced only IL-6 in the blood 3 h after prime. To test whether IL-6 was a potential COP, we correlated IL-6 release 3 h post-prime and post-boost with efficacy in individual females and males (Fig. 10). The data indicate a trend in which elevated IL-6 in the blood is associated with reduced morbidity and mortality after challenge. The trend is particularly striking after the prime, where the response to T-VANT is elevated compared to post-boost.

## Discussion

For most individuals, seasonal influenza vaccines are administered without adjuvants, despite the well-known advantages that adjuvants confer on the magnitude, durability, and breadth of the response^7,39^. The aim of this study was to assess the effect of 5 different novel adjuvants on the immunogenicity and efficacy of two seasonal influenza vaccines in a murine influenza model. A low dose of antigen was used to reveal effects of the adjuvants, specifically on innate immunity, including the breadth of inflammatory cytokines produced and reactogenicity, as well as on the adaptive immune response, including magnitude and breadth of the antibody response, T cell response and Th1/Th2 ratio.

Of all the metrics captured, inflammatory cytokine profiling of blood shortly after vaccination revealed the greatest differences between the adjuvants (Table 2). T-VANT produced the broadest profile, with changes in at least 11 of 13 the inflammatory cytokines measured relative to the antigen only control. TRAC-478, IVAX-3 and -1, produced narrower profiles of 2 or 3 cytokines, while non-adjuvanted seasonal vaccines and AHQ-II induced no changes relative to the control. T-VANT comprises outer membrane vesicles (OMVs) from the ΔPurM mutant strain of *Burkholderia pseudomallei*, which has a naturally attenuated LPS. The relatively large number of inflammatory cytokines induced by T-VANT is consistent with its multiple pathogen-associated molecular patterns (PAMPs) that are responsible for driving both humoral and diverse cellular immune responses^29–31^. It is important to note that the lack of a particular cytokine(s) from a particular adjuvant does not preclude the release of other cytokines that are not represented in the 13-plex panel used here, or cytokines that *are* represented in the panel but released at other time points not tested here.

An important consideration in adjuvant selection is the balance between immunogenicity and reactogenicity. Most approved vaccines will induce mild reactogenicity in a subset of individuals, such as muscle or joint pain, or injection site pain or soreness, which is considered an acceptable tradeoff for vaccine efficacy. We observed transient weight loss of up to ~10% of original body weight in mice immunized with T-VANT, TRAC-478, IVAX-3 and IVAX-1 containing formulations, which was maximal on d1 but which was quickly regained 2-4 days later (Fig. 2). Similar levels of weight loss were seen after prime and boost, suggesting none of these adjuvants sensitized the animals for a more severe or anaphylactic reaction upon secondary exposure. In fact, with the exception of IFN-γ, the most abundant cytokines secreted following prime immunization were not significantly elevated following the boost. The rapidity with which the weight was lost and regained suggests this was associated with a temporary reduction of food intake, although additional studies are needed to confirm this. Transient weight loss in mice after vaccination has been used in other studies as a measure of vaccine reactogenicity or toxicity^40–44^ and may be an indicator of concern. However, the significance of the weight loss in mice in the context of human vaccination is not well understood. Weight loss is not typically noted following vaccination in humans, but other local and systemic indicators of an innate immune response, including fever, myalgia, headache etc. are typically experienced after vaccination^45^. Whether the inflammatory reactions that underlie transient weight loss in mice would also mediate vaccine-related local or systemic reactions in humans, needs clarification. The positive control vaccine, FluAd®, induced only IL-6 (of the 13 assayed). Since FluAd® did not induce transient weight loss soon after i.m. administration, this supports the notion that IL-6 alone does not explain the weight loss (Fig. 3).

The association between inflammatory cytokines and the level of weight lost is dose dependent (Fig. 3) suggesting blood concentrations could be used as predictors of the severity of a reactogenic response. Inflammatory cytokines produced in response to adjuvanted vaccines, such as IL-6, TNF-α, and interferons, are an essential component of vaccine immunogenicity^46–48^, and many of the same inflammatory cytokines are also associated with reactogenicity^45,49,50^. For example, in humans systemic reactogenicity following administration of Hepatitis B surface antigen (HBsAg) adjuvanted in AS01_B_ (MPLA in liposomes with saponin QS-21) is associated in particular with IL-6 and C-reactive protein, and IFN-γ post-boost^47^. Overall, the magnitude of inflammatory responses was associated with immunogenicity and the incidence of reactogenicity. More recently, a systems immunology approach to profiling two investigational COVID-19 vaccines found a positive correlation between vaccine-induced systemic cytokines (particularly IFN-α, IFN-γ, IP-10, CXCL11, MCP-1, and IL-10), reactogenicity, and adaptive immunity^50^. This study again highlights the importance of innate immunity in achieving the balance between vaccine efficacy and low reactogenicity. Live attenuated VSV vaccine induces transient weight loss after i.m. injections in mice^40^ and elevated blood IL-1β. Reactogenicity was reduced in mice deficient in IL-1 receptors but were still protected against lethal VSV challenge, demonstrating that IL-1 contributed to reactogenicity but was redundant in the protective response. IL-1β is a signature cytokine produced by inflammasome activation leading to pyroptosis^51^. In our hands, however, none of the adjuvants we tested induced significant IL-1α or IL-1β 3 h after prime or boost (Supplementary Fig. 1), which may be related to differences between live/attenuated vs. inactivated vaccine modalities.

Inflammatory cytokine profiling in the blood also revealed differences after prime and boost. Notably, there were large increases in IFN-γ production by Th1-skewing adjuvants TRAC-478, IVAX-1 and IVAX-3 after the boost compared to the prime, which is consistent with restimulation of antigen-specific memory Th1 cells in vivo by the boost. We also found a considerable reduction of IL-6, CCL2 and TNF-α produced by T-VANT after the boost compared to the prime.

In this study we noted T-VANT, TRAC-478, IVAX-3 and IVAX-1 all engendered a balanced or Th1-polarized response, while AHQ-II induced a Th2 response. We are aware this contrasts with the Th1 response induced by AHQ-II with whole inactivated SARS-CoV-2 virions (BBV152)^52,53^, or recombinant spike (S) protein^54^. AHQ-II was developed from a systematic screen of TLR8-specific and dual TLR7/8-active compounds in cell-based stimulation assays^55^. In clinical trials, adjuvanticity was improved when combined with aluminum gel^52^, and the resultant combination is the adjuvant used in the approved inactivated whole SARS-CoV-2 monovalent virus vaccine, Covaxin® (Bharat Biotech). In our hands, a IgG1/Th2 response from AHQ-II was seen consistently across multiple independent experiments, i.e., with both Flublok® and Fluzone HD®, adjuvant alone, and in both female and male cohorts (Fig. 4C, D, Supplementary Fig. S2). The Th2 bias was also confirmed in T cell recall assays in both ELISpot and supernatant multiplex assay formats (Fig. 6). One possible explanation for this contradiction is that the Th1/Th2 ratio may be influenced by the inherent properties of the antigen without adjuvant. For example, non-adjuvanted Flublok and Fluzone HD both induced a weak but clearly Th2-polarized response (Figs. 4C and 5C), which was amplified by AHQ-II. In contrast, BBV152 antigen is inherently Th1-polarizing (i.e., it induces 3-fold higher titers of IgG2a than IgG1)^53^, which may contribute to the Th1 polarization after administration in AHQ-II. (Recombinant S is insufficiently immunogenic in the absence of adjuvant to determine whether it is inherently Th1 or Th2 biasing^54^). Another possible explanation for the contradiction may come from the differences between TLR-7 and -8 mice. In human cell-based assays, the TLR7 agonist was responsible for mainly IFN-α/β production, while TLR-8 induced the Th1 cytokines, IFN-γ and TNF-α^55^. In mice, TLR-8 was originally thought to be non-functional, which would be consistent with a reduced Th1 response in mice, although more recent data show TLR-8 functionality^56^. Overall, the activities of murine TLR-8 are less well understood than TLR-7, but differences that may emerge in the future may help explain this apparent contradiction.

The other adjuvants tested here, which were Th1-biasing, would appear to be able to overcome the natural tendency of non-adjuvanted Flublok® and Fluzone HD® to polarize towards Th2. Previous studies of IVAX-1 indicate it is a Th-1 skewing adjuvant that has shown efficacy against different pathogens, including SARS-CoV-2^57^, *Coxiella burnetii*^58^ and *Chlamydia muridarum*^59^. IVAX-1 and IVAX-3 are identical except for the usage of CpG ODN 1018 and 55.2, respectively. In this study, their performance in mice was very similar, suggesting both CpGs are active on murine TLR-9, although CpG 55.2 is more potent than 1018 in assays with human TLR9 reporter cell lines (Nikolai Petrovsky, *Pers. Comm*). TRAC-478 is a liposome-based delivery system for synthetic agonists of TLR-4 and TLR-7/8 which has been previously reported to induce Th1 immunity^60,61^. Similarly, T-VANT produces robust Th1/Th17 responses^29–31^.

Ex vivo antigen-restimulation assays by ELISpot and supernatant cytokine assay were performed to assess T cell responses induced by the adjuvants. Cytokine profiling corroborated the Th1/Th2 bias inferred from the IgG subtyping data. Also of interest was the temporal dynamics of these responses, which indicated Th2 cytokines induced by AHQ-II peak earlier than Th1 cytokines induced by the remaining adjuvants (Fig. 6). This observation indicates that the optimal time-point for measurement of T cell responses is likely to be different for the various T cell subsets, and which may inform future studies designed to compare and characterize adjuvants. Also noteworthy was the lack of a detectable H1-specific T cell response ex vivo, which seems remarkable given each adjuvant induced H1-specific Ab. Accordingly, we also saw no cross-reactivity for the closely-related H5 antigen, which is typically seen at the Ab level when using adjuvants^33,34^. The H1 variant used for the assay matched that of the 22/23 seasonal vaccines used to immunize, so the lack of recall response is not caused by a sequence mismatch. We have reported the relatively weak immunogenicity of H1 in C57Bl/6 mice previously^33^. Although we have not tested immunogenicity in other murine strains, it is possible H1 lacks sequences able to bind strongly to I-A^b^, thereby producing a weak CD4 response that was below the detection threshold of our recall assays. This is unlikely to be an issue in humans where multiple class II alleles are available for presentation.

Influenza virus-neutralizing antibodies (nAbs) are traditionally used to assess vaccine efficacy^37^. Consistent with this, we noted a trend for higher nAb titers to be associated with protection from morbidity and mortality (Fig. 9). However, there were many individual animals that were protected that had low nAb titers, indicating a role for other immune mechanisms in protection. We also noted males showed more morbidity than females, despite having neutralizing antibodies. Males also showed slightly lower antibody titers by ELISA and microarray (Fig. 4), although we detected no sex bias in nAb titers (Fig. 5). Other studies in mice and humans have also reported that males are less responsive to influenza vaccines compared to age-matched females^62–64^. Currently, several human vaccine formulations are adjusted for age^65^, but not for sex. The data presented here would support the notion that vaccine formulations should be adjusted for sex as a biological variable^66^.

Protection against influenza is likely to involve multiple immune mechanisms, and other COPs in addition to antibody-mediated neutralization are needed to fully assess vaccine efficacy^67^. To address this we examined early cytokine levels after prime and boost in individual mice and correlated with efficacy. IL-6 was of particular interest because of the ~5-log range of IL-6 production induced by different adjuvants that trends with efficacy (Supplemental Fig. S1I), and because FluAd®, which is a protective vaccine, induced only IL-6 production (Table 2). IL-6 is a pleiotropic cytokine with many functions in inflammation, hematopoiesis, bone metabolism and embryogenesis^68^. In the immune system, IL-6 promotes the early development of Tfh cell differentiation and the germinal center reaction^69^. IL-6 has also been proposed as a key cytokine in the adjuvanticity of cationic lipid nanoparticles^70^.

The data in this study also demonstrate the potential for adjuvants for vaccine dose-sparing. Prior studies have shown adjuvants allow lower doses or single doses of vaccine to be used without compromising immunogenicity or efficacy^36,71–74^. In this study, we first defined low doses of Flublok® and Fluzone HD® to enable the effects of the adjuvants tested to be more clearly seen (Fig. 1A–C). The dose we used (0.5 μg/HA/dose) was poorly immunogenic and did not confer protection against H1N1 challenge, but became immunogenic and efficacious when delivered with adjuvants (Figs. 4–7). Low dose and single-dose vaccination may be important when vaccine supply is limited, in resource constrained settings, or in emergencies where population coverage is needed quickly, such as in pandemics.

The mechanisms by which parenteral routes of vaccination, such as i.m., induce mucosal immunity are not well understood. Parenteral routes generally fail to induce mucosal IgA, and yet i.m delivery provides robust protection against many respiratory pathogens. In this study, the ability of the different adjuvants to induce mucosal immunity was not directly addressed. However, we have observed that parenteral (sub-cutaneous) injection of SARS-CoV-2 spike protein adjuvanted in IVAX-1 induces antigen-specific IgG (but not IgA) in lungs as well as in the blood (Hernandez-Davies et al, manuscript in preparation). Others have reported similar findings. For example, i.m. administration of spike mRNA vaccine induces IgG in serum and saliva of human vaccinees^75^; the authors reported a close correlation between serum and saliva IgG titers, suggesting serum IgG may be transported from the blood to mucosal surfaces, possibly via an Fc receptor-mediated process.

A weakness of the study includes using a single dose of each adjuvant. The concentrations used were recommended by the developers, but it is clear that immunogenicity is influenced by both adjuvant and antigen dose. An adjuvant that appears to have weaker immunogenicity than other adjuvants may perform better at a different dose, although this may also be a trade-off against reactogenicity. In this context, AHQ-II and FluAd conferred less than 100% survival but were non-reactogenic, while the other adjuvants conferred 100% survival but were more reactogenic. Other weaknesses of the study include the single time-point used for inflammatory cytokine measurements; ideally these should be collected at multiple time points to account for different release dynamics and half-lives of the cytokines. Durability of the response was also not a metric captured. Finally, heterologous challenge using a virus of a different subtype from the vaccine, such as H5N1, instead of the mismatched H1N1 as used here, may have revealed more compelling differences between the adjuvants.

In summary, we have performed a side-by-side comparison of several novel adjuvants in the same disease model. We find adjuvant-specific differences in immunogenicity and efficacy, although all the adjuvants tested improve the performance of seasonal influenza administered at low dose. Studies such as this will help inform rational adjuvant selection and guide vaccine development for influenza and other infectious diseases.

## Methods

### Vaccines and adjuvants

Quadrivalent 2022/2023 seasonal influenza vaccines Flublok® (Sanofi), Fluzone HD® (Sanofi) and FluAd® (Seqirus) were obtained from Thermofisher Bioservices (Germantown, MD). Each comprised A/Wisconsin/588/2019 or A/Victoria/2570/2019 (H1N1), A/Darwin/6/2021 or A/Darwin/9/2021 (H3N2), B/Austria/1359417/2021 or B/Michigan/01/2021, and B/Phuket/3073/2013. The adjuvants screened in this study comprised: (1) Alhydroxyquim-II^54,55^ (a gift from Sunil David, ViroVax Inc.) which consists of the toll-like receptor (TLR) 7/8 agonist, imidazoquinoline, chemisorbed on Alhydrogel® aluminum hydroxide hydrogel; (2) T-VANT^29–31^ (a gift from Lisa Morici and James McLachlan, Tulane University), which consists of outer membrane vesicles (OMVs) from the ΔPurM mutant strain of *Burkholderia pseudomallei* which has a naturally attenuated LPS; (3) TRAC-478^32^ (gift from Jay Evans, Inimmune Inc), which consists of synthetic TLR4 and TLR7/8 ligands co-delivered in a stable nanoemulsion; (4) IVAX-1^33–36^, which consists CpG-1018 (Integrated DNA Technologies, Coralville, Iowa), phosphoryl-lipid A (MPLA; Avanti Polar Lipids Inc., Alabaster, AL) and AddaVax™ emulsion (Invivogen Inc., San Diego, CA); (5) IVAX-3, a novel combination that has not been reported previously, but which is identical to IVAX-1 except CpG-1018 is replaced with CpG 55.2^76^ (a gift from Nikolai Petrovsky, Vaxine Pty Ltd, Australia).

### Viruses

Influenza virus A/California/07/2009 (HA, NA) x A/Puerto Rico/8/1934 (H1N1)pdm09 reassortant NYMC X-181^77^ (“Cal09/PR8”) (BEI Resources, Manassas, VA; catalog #NR-44004) was propagated in fertilized chicken eggs^78^ and used for virus neutralization assays and challenge studies. Titers of virus stocks were determined on MDCK cells (ATCC Cat. # CCL-34) to give TCID_50_/mL^79,80^. H1 from Cal09/PR8 and the 2022/2023 vaccines differ by 29 amino acids, of which 20 are in the HA1 fragment.

### Immunizations

All animal work was approved by the UCI Institutional Animal Care and Use Committee (IACUC) Protocols and AUP-21-067 and AUP-21-133. The laboratory animal resources at UCI are Internationally accredited by the Association for Assessment and Accreditation of Laboratory Animal Care (AAALAC #000238). Female C57Bl/6 mice (8 weeks of age) and males (purchased at 3 weeks of age and allowed to mature together to 8 weeks of age) were purchased from Charles River Inc. and housed in standard cages with nesting materials for enrichment. Mice were administered formulations on days 0 and 14 in a volume of 50 μL using an insulin syringe and needle via the intramuscular (i.m.) route (left caudal thigh) after transient anesthesia in an isoflurane/O_2_ mixture delivered using a vaporizer/scavenger apparatus. After the procedure, the animals were returned to their cage and allowed to recover consciousness. All animals were weighed daily and monitored for appearance and behavior. For plasma collection, mice were transiently anesthetized in an isoflurane/O_2_ mixture, and blood was collected into heparinized microcapillary tubes (Microvette® CB 300 Lithium heparin from Sarstedt) using a sterile 25–27 gauge needle to puncture the facial vein. Blood was collected 3 h post-prime and post-boost for quantification of inflammatory cytokines (IL-23, IL-1α, IFN-γ, TNF-α, CCL2 (MCP-1), IL-12p70, IL-1β, IL-10, IL-6, IL-27, IL-17A, IFN-β, and GM-CSF) using LEGENDplex™ kits (BioLegend Inc, San Diego, CA), and on d28 for serological analysis by ELISA, protein microarrays and virus neutralization assays. Plasma sample aliquots were stored at −80 °C until required for assay. At the experimental endpoint, mice were humanely euthanized in their home cage by gradually filling to 100% CO_2_ from a cylinder utilizing a displacement rate from 30–70% of the chamber volume per minute, followed 1–2 min after breathing had ceased by cervical dislocation as a secondary method.

Initially, vaccine dose-ranging studies were performed in female mice to determine a low dose of vaccine that conferred protection only when adjuvanted. FluAd, which is supplied at the lowest HA concentration of the three commercial vaccines tested, defined the highest dose for mouse immunizations (i.e., 15 μg/HA/500 μL human dose = 1.5 μg/HA/50 μL mouse dose). Concentrations of HA were adjusted while maintaining the concentration of all excipients. Thus, Flublok® and Fluzone HD® were diluted to 1.5 μg and 0.5 μg doses in the appropriate suspension buffer according to the manufacturer’s package insert. FluAd was used neat (1.5 μg dose) or diluted 1:3 or 1:9 (0.5 μg and 0.17 μg doses, respectively) in a 1:1 mixture of AddaVax™ (Invivogen) in water. This diluent mimics the composition of FluAd (1:1 MF59:antigen solution) which preserves the concentrations of squalene and stabilizers in the emulsion and helps to maintain its stability.

### ELISAs and protein microarrays

IgG titers were determined by ELISAs as described^81^. Briefly, 96-well microtiter plates (Reacti-Bind™ from Thermo Fisher Scientific, Waltham, MA) coated with 2 μg/mL recombinant H1 from A/Wisconsin/588/2019 expressed in HEK293 cells (Sinobiological Inc., Cat. #40787-V08H) in TBS (20 mM Tris/150 mM NaCl, pH 7.6) at 4 °C overnight were washed 4x in T-TBS (TBS containing 0.05% Tween 20) and blocked for 1–2 h with casein/TBS blocking buffer (Thermo Fisher Scientific, Waltham, MA; Cat #37583). Plasma from mice receiving PBS was used as controls. Five-fold serial dilutions of plasma in blocking buffer, starting at 1:100, were incubated in pre-coated plates, followed by washing in T-TBS with secondary antibody (goat anti-mouse IgG-HRP conjugate from Bethyl Laboratories, Montgomery, TX) diluted to 1:12,500, and stabilizing buffer (Guardian™ from Thermo Fisher Scientific) for 45min. After washing, plates were developed in 3,3′,5,5′-tetramethylbenzidine (TMB) peroxidase substrate (SureBlue Reserve™ from SeraCare KPL, Gaithersburg, MD; Cat. No. 5120-0075) and reactions stopped using 0.2 M H_2_SO_4_. Absorbances were measured at 450 nm using a FilterMax-F5 plate reader (Molecular Devices, San Jose, CA). OD at 450nm vs. the reciprocal serial dilution graph was generated, and the midpoint titer was determined by non-linear sigmoidal, 4PL least squares fit, where the dilution yields 50% of maximum OD450. Breadth and magnitude of IgG were determined using custom protein microarrays^34,82^. Briefly, purified HA and other influenza antigens (Sino Biological Inc.) were printed on nitrocellulose-coated glass slides (Grace Biolabs, Bend, OR) using an OmniGrid 100 microarray printer (Genomic Solutions). The content of the array was as reported previously^83^. For probing, plasma samples were diluted 1:100 in protein array blocking buffer (GVS FAST™ from ThermoFisher) supplemented with a poly-histidine peptide (Biomatik USA, Delaware, USA) to final concentration 0.10 μg/mL to block any antibodies to poly-Histidine tags. After rehydration, arrays were incubated in diluted plasma overnight at 4 °C, then washed in T-TBS. Bound IgG was visualized using biotinylated anti-mouse IgG (Jackson ImmunoResearch; Cat No. 115-068-071), followed by washing and incubation in streptavidin-conjugated Qdot-800 (Life Technologies; Cat. No. Q10173MP) diluted 1:250 in the blocking buffer. For IgG subtyping, anti-mouse IgG1-Alexa Fluor647 or IgG2c-Alexa Fluor555 (Southern Biotech; Cat. Nos. 1073-31 and 1077-32) were used as secondary antibodies. After washing and drying, scanned images were acquired using the ArrayCAM imaging system (Grace Bio-Labs Inc., Bend, OR). Signal intensities (SI) for each antigen on the array were first background-corrected by subtracting sample-specific T-PBS buffer signals from purified protein spot signals. Quantile normalization was conducted to reduce assay to assay variation as previously described^84^. Data were plotted and statistical analyses performed using Prism software version 10.6.0 (GraphPad Inc., San Diego).

### Virus neutralization assays

Hemagglutination Inhibition (HAI) assays were performed as described^85^. Mouse plasma samples were tested in duplicate by first treating 10 μL plasma with 30 μL receptor destroying enzyme (RDE; Denka Seiken, Inc.; typically supplied at 200-300 units/ml) for 18 h at 37 °C, after which 30 μL of 2.5% sodium citrate was added and heated at 56 °C for 30 min, according to the manufacturer’s instructions. The volume was brought up to 100 μL with PBS, pH7.2, to give a starting dilution of 1:10. Duplicate serial dilutions of 25 μL plasma/well were performed across a V-bottomed microtiter plate (Thermo Fisher) in HAI assay buffer (FTA Hemagglutination Buffer, Fisher Scientific) and an equal volume of virus (diluted to 4 hemagglutination units [HAU]/25 μL HAI assay buffer) added for 30 min at 18 °C to allow neutralization to occur; control wells contained virus only or HAI assay buffer only. To each well was then added 50 μL 0.5% freshly-washed turkey red blood cell suspension (Rockland Immunochemicals, Inc., Limerick, PA) in HAI buffer and left for 45 min at 4 °C to allow red cell pellets to form. The neutralization titer was defined as the reciprocal of the last dilution in which a clear pellet was seen and multiplied by 10 to correct for the initial dilution from RDE treatment. In humans, a serum sample is considered seroprotective when it shows an HI titer ≥ 40^37^. Hyperimmune sera for positive controls were produced in C57Bl/6 against Cal09/PR8 by boosting survivors of an intranasal challenge 2 weeks later with the same dose (5 × 10^4^ TCID_50_/ml) in 100 μL via the intraperitoneal (i.p.) route.

Microneutralization (MN) assays were performed as described^86^ with modifications. Briefly, MDCK cells were obtained from ATCC (Manassas, VA; Cat. No. CCL-34) and maintained in Eagle’s minimum essential medium (EMEM; ATCC Cat. No. 30-2003) containing penicillin/streptomycin (Thermofisher) and 10% heat-inactivated fetal calf serum (ATCC-Cat. No. 30-2020), and cultured at 37 °C/5% CO_2_ in a humid environment. Cells were passaged when 80–85% confluent; only early passage cells were used for MN assays. One day prior to assay, cells were subcultured into flat-bottomed 96 well plates at 2 × 10^4^ cells/well in 100 µL. Plasma samples were treated overnight with 3 volumes of RDE for 18 h at 37 °C, and then inactivated at 56 °C for 30 min. Samples were then diluted 1:10 in virus growth medium (which comprised serum-free EMEM containing 0.6% BSA and 1 µg/mL N-p-Tosyl-l-phenylalanine chloromethyl ketone [TPCK]-treated trypsin [Worthington Biochemical]), and two-fold serially diluted in virus growth medium in a separate 96-well plate. Reassortant Cal09/PR8 was diluted to 100 TCID_50_ per 50 µL in virus growth medium, and 50 µL of virus was added to wells containing the serially diluted plasma; control wells contained virus only or growth media only. After 1 h incubation, the media above the cell monolayers was then replaced with the serum-virus mixtures and incubated for 1 h. Serum-virus mixtures were then removed and replaced with 200 µL of virus growth media + 2% fetal bovine serum, and plates were incubated for 48 h at 37 °C/5% CO_2_. Cells were then rinsed in PBS and fixed in 4% paraformaldehyde (Fisher Scientific; Cat. No. AAJ19943K2) in PBS for 30 min, washed in PBS, and permeabilized in 0.1% PBS/Triton X-100 at 18 °C for 15 min. After washing in PBS, cells were blocked in 3% BSA (Sigma-Aldrich, Cat. #A9085) in PBS for 1 h at 18 °C. Influenza virus nucleoprotein (NP) was then detected using anti-NP mAbs (Millipore Cat Nos. MAB 8257 and MAB 8258) combined at 1:1000 dilution each in blocking buffer, followed by horseradish peroxidase (HRP)-conjugated anti-mouse IgG (KPL, Cat. # 074-1802) diluted to 1:3,000 in blocking buffer. Plates were developed in TMB developer (SureBlue Reserve™) and reactions stopped using 0.18 M H_2_SO_4_. Assays were quantified in an enzyme-linked immunosorbent assay (ELISA) plate reader (Emax Plus, Molecular Devices LLC, San Jose, CA) at 450 nm using SoftMax Pro 7.1 software, and midpoint titers calculated in Prism (GraphPad, San Diego, CA).

### T cell recall assays

Recall assays were performed at 9 days post-prime and boost by IFN-γ/IL-4 ELISpots, and by cytokine assay of culture supernatants (LEGENDplex kit from BioLegend Inc., San Diego, CA) as described^35^. Proteins for T cell restimulation (Sino Biological Inc.,) matched the variants used in the 2022/2023 seasonal vaccines used for immunization, i.e., H1 from A/Wisconsin/588/2019/ A/Victoria/2570/2019 (Cat. #40787-VO8H); H3 from A/Darwin/6/2021 (Cat. #40868-V08H); and influenza B HA from B/Austria/1359417/2021 (Cat. #40862-VO8H). H5 from A/Vietnam/1194/2004 (Cat. # 11062-V08H1) was also included to assess cross-reactivity of the T cell response. Assays were performed in Iscove’s Modified Dulbecco’s Medium (IMDM), containing 5 × 10^−5^ M β-mercaptoethanol, 100 IU/mL penicillin, 100 μg/mL streptomycin, and 10% heat-inactivated fetal calf serum in the presence of antigen at final concentrations of 10, 1, 0.1 and 0 μg/mL. T cell mitogen, Concanavalin A (Sigma-Aldrich), was used as a viability control. After 18 h of incubation, the assay supernatants were collected and stored at -80^o^C for multiplex cytokine screening and the plates then processed for ELISpot.

### Virus challenge and lung titer by qPCR

Mice were challenged on d40 with the H1N1 reassortant A/California/07/2009 (H1N1) x A/Puerto Rico/8/1934 (Cal09/PR8). Transiently anesthetized mice (*N* = 8/group) were administered 50 μL of reassortant Cal09/PR8 at 10^4^ TCID_50_/mL via the intranasal (i.n.) route and monitored daily for appearance, behavior, and body weight until the endpoint (d10-12 after challenge or until the mice lost >20% or their original body weight) after which the mice were humanely euthanized as described above. To quantify lung virus titers, lungs were collected from mice (*N* = 3/group) on d3 post-challenge into pre-weighed cryotubes, snap-frozen on dry ice and stored at −80 °C until required. Total RNA was extracted by homogenizing weighed lung tissue in 1 mL of TRIzol (Thermo Fisher Scientific) using a GentleMacs Tissue Homogenizer (Miltenyi Biotec) using the preset RNA-01 program, followed by phase separation in Phasemaker™ tubes and total RNA extraction according to the manufacturer’s guidelines (Thermo Fisher Scientific). Total RNA was then resuspended in 100 µL of ultrapure RNAse/DNase-free distilled water and stored at −80 °C. RT-qPCR amplification was performed according to the WHO guidelines for molecular detection of influenza viruses^87^. The H1 gene was amplified using forward primer NIID-swH1 TMPrimer-F1 (5′-AGAAAAGAATGTAACAGTAACACACTCTGT-3′), reverse primer NIID-swH1 TMPrimer-R1 (5′-TGTTTCCACAATGTAGGACCATG-3′0), and TaqMan probe NIID-swH1 probe2 (5′-56-FAM-TGGGTAAAT-ZEN-GTAACATTGCTGGCTG-3IBkFQ-3′). As a positive amplification control, glyceraldehyde-3-phosphate dehydrogenase (GAPDH) from *Mus musculus* was used using primers GADPH-Fw (5′-CAATGTGTCCGTCGTGGATCT-3′), GADPH-Rv (5′-GTCCTCAGTGTAGCCCAAGAT-3′), and the TaqMan probe GADPH probe 5′-SUN-CGTGCCGCC-ZEN-TGGAGAAACCTGCC-3IABkFQ-3′^88,89^. Quantitative RT-PCR was performed using AgPath-ID™ One-Step RT-PCR Reagents (Thermo Fisher Scientific) following the manufacturer’s instructions. Briefly, 20 µl of master mix containing each primer at 0.5 µM, each probe at 0.2 µM, 1x of Q-RT-PCR Master Mix and 1 x of QuanTec RT-PCR enzyme mix, plus the inhibitory of RNases (RNaseout from Thermo Fisher Scientific) at 0.4 mM was added to 5 µl of the total extracted RNA and amplified at 50 °C for 10 minutes, 95 °C for 10 minutes, and 45 cycles of 95 °C for 10 seconds, 56 °C for 30 seconds (collection data), and 72 °C for 15 seconds. For quantification, an HA standard curve was produced using log_10_ serial dilutions of a synthetic linear DNA that contains one copy of the HA gene (8.3 × 10^7^ to 8.3 copies/reaction). A standard curve was generated in parallel with each amplification to estimate the number of HA copies amplified in each sample. The total copies of HA gene present in the total RNA extraction were normalized against the weight of lungs (expressed as genomic RNA copies/mg lung). The theoretical limit of detection (LOD) was estimated to be 10 copies of H1 gene per reaction, based on standard curve data performed previously. The range of LODs were defined as the minimum and maximum values and expressed as genomic RNA copies/mg lung.

### Statistical analyses

Graphical outputs and statistical analyses were performed in Graphpad Prism 10.6.0 and ggplot2 v3.4.2 using R statistical software (http://www.r-project.org). For the majority of assays, P values were calculated for males and females separately using one-way ANOVAs between vaccine groups and the corresponding antigen-only (no adjuvant) control group to determine adjuvant effect. For blood cytokines post-prime and boost, each vaccine group was compared with the antigen-only control group using a two-tailed Wilcoxon nonparametric test, followed by Benjamini and Hochberg (BH) correction to control for false discovery rate for multiple comparisons using R.

## Supplementary information

Supplemental Informations

## Data Availability

The datasets generated and/or analyzed during the current study are available in the immport repository, https://www.immport.org/shared/study/SDY3299
