# Supplementary material for: Comparative evaluation of 5 combination adjuvants on immunogenicity and efficacy of approved seasonal influenza vaccines
Source: NPJ Vaccines. 2026 Jan 27;11:30. doi: 10.1038/s41541-025-01339-y (PMC12852915; doi:10.1038/s41541-025-01339-y)

Figure S1. Relationship between inflammatory cytokines and transient weight loss. Female and male mice were administered low-dose seasonal vaccines in different adjuvants (Table. 1). Concentration of cytokines in blood (pg/mL) at 3h post-prime and -boost are shown plotted against the corresponding weight for each mouse on d1 post-prime or boost (data from Fig. 2). Abbreviations: Fb, Flublok; Fz, Fluzone HD; Adj., adjuvant; Ag., antigen.

Figure S1A:

IL-23

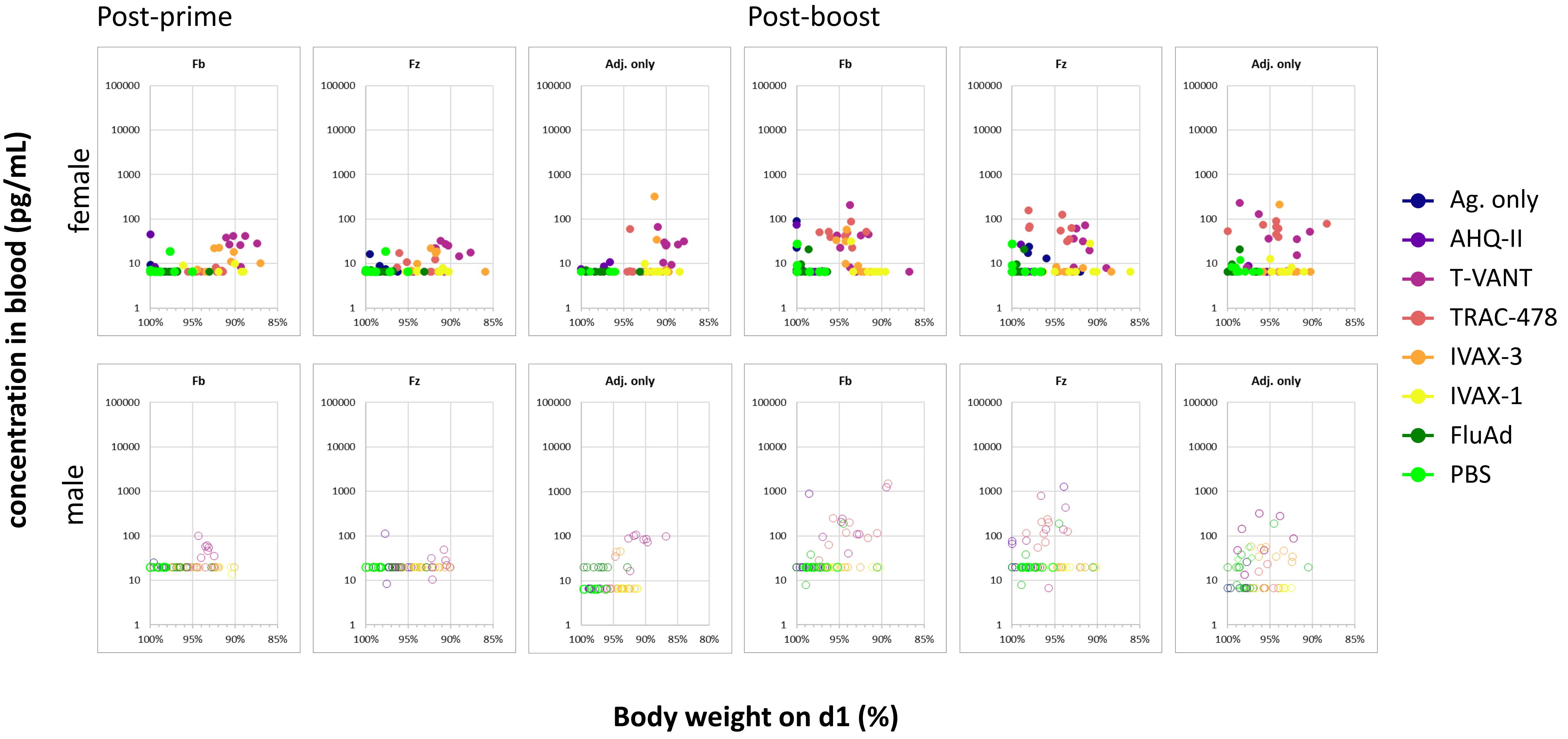

Figure S1B:

IL-1α

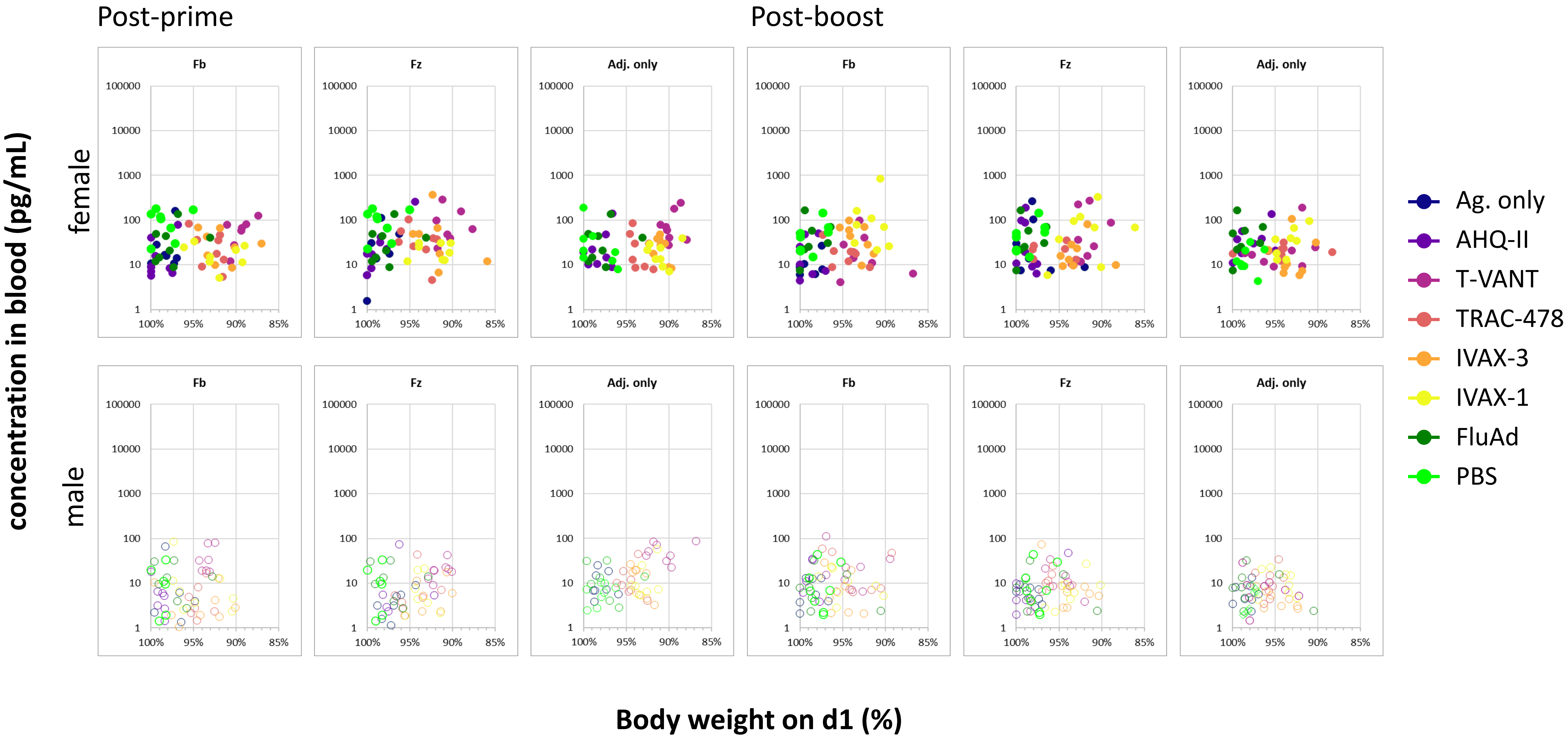

Figure S1C:
IFN-γ

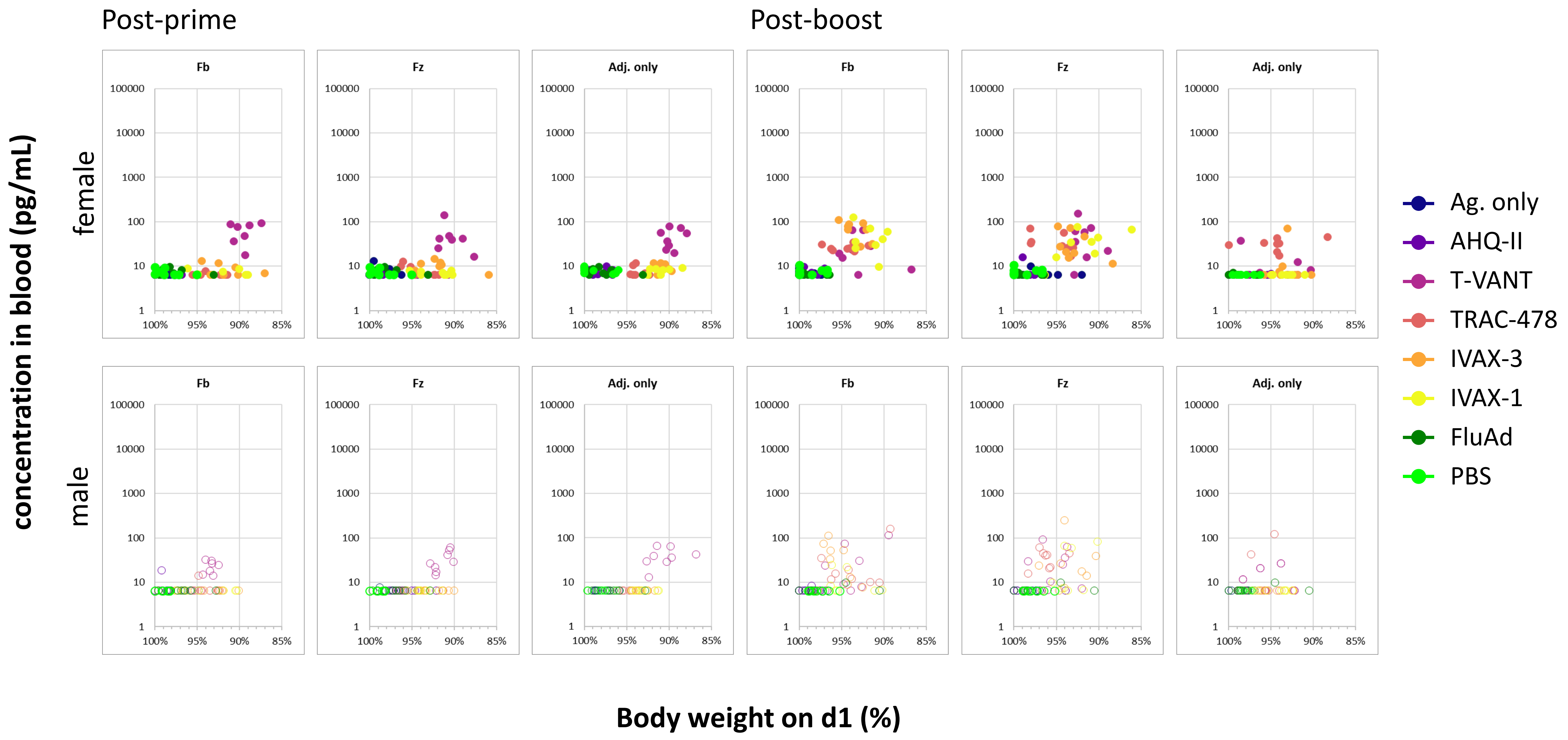

Figure S1D:

TNF-α

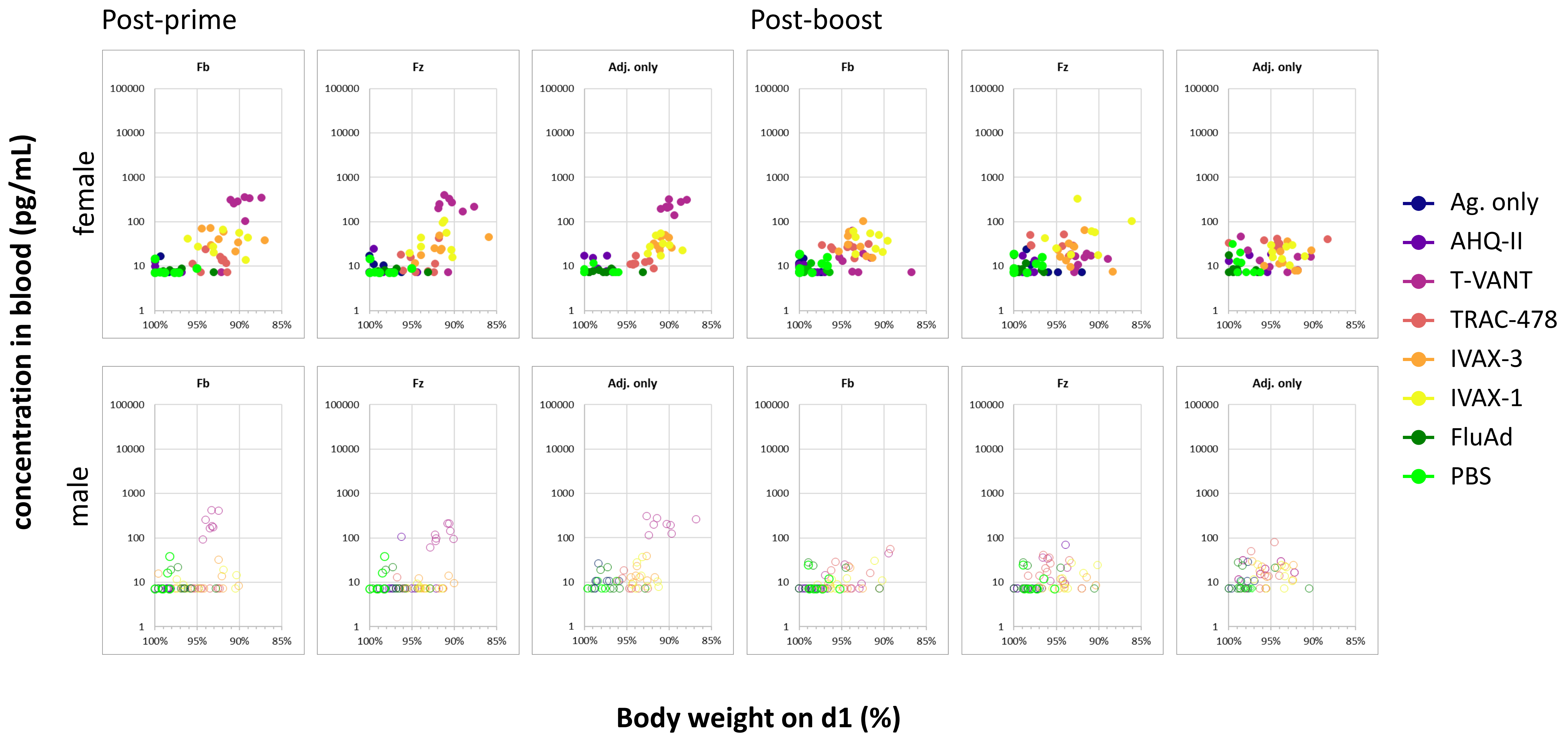

Figure S1E:
MCP-1/CCL2

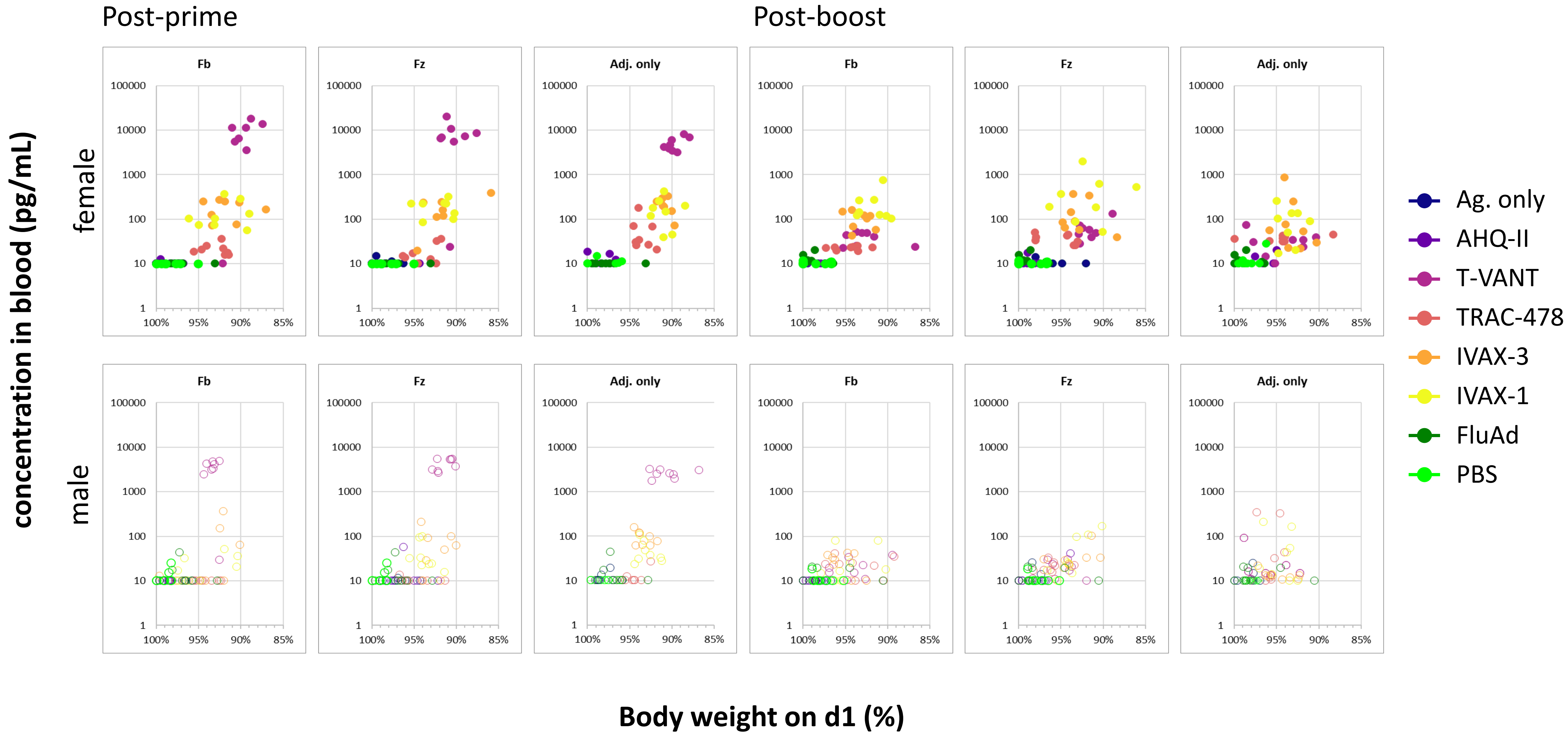

Figure S1F:

IL-12p70

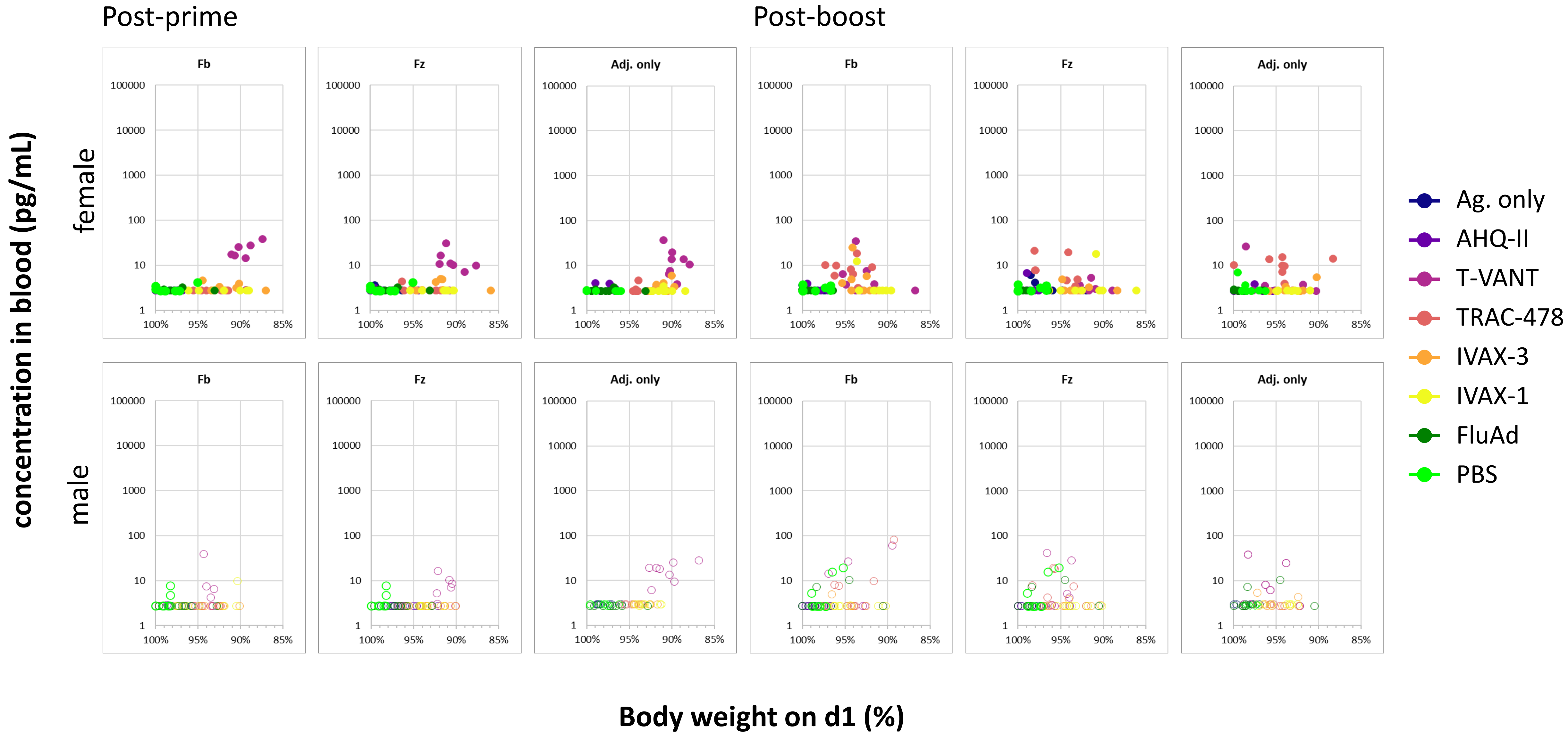

Figure S1G:

IL-1β

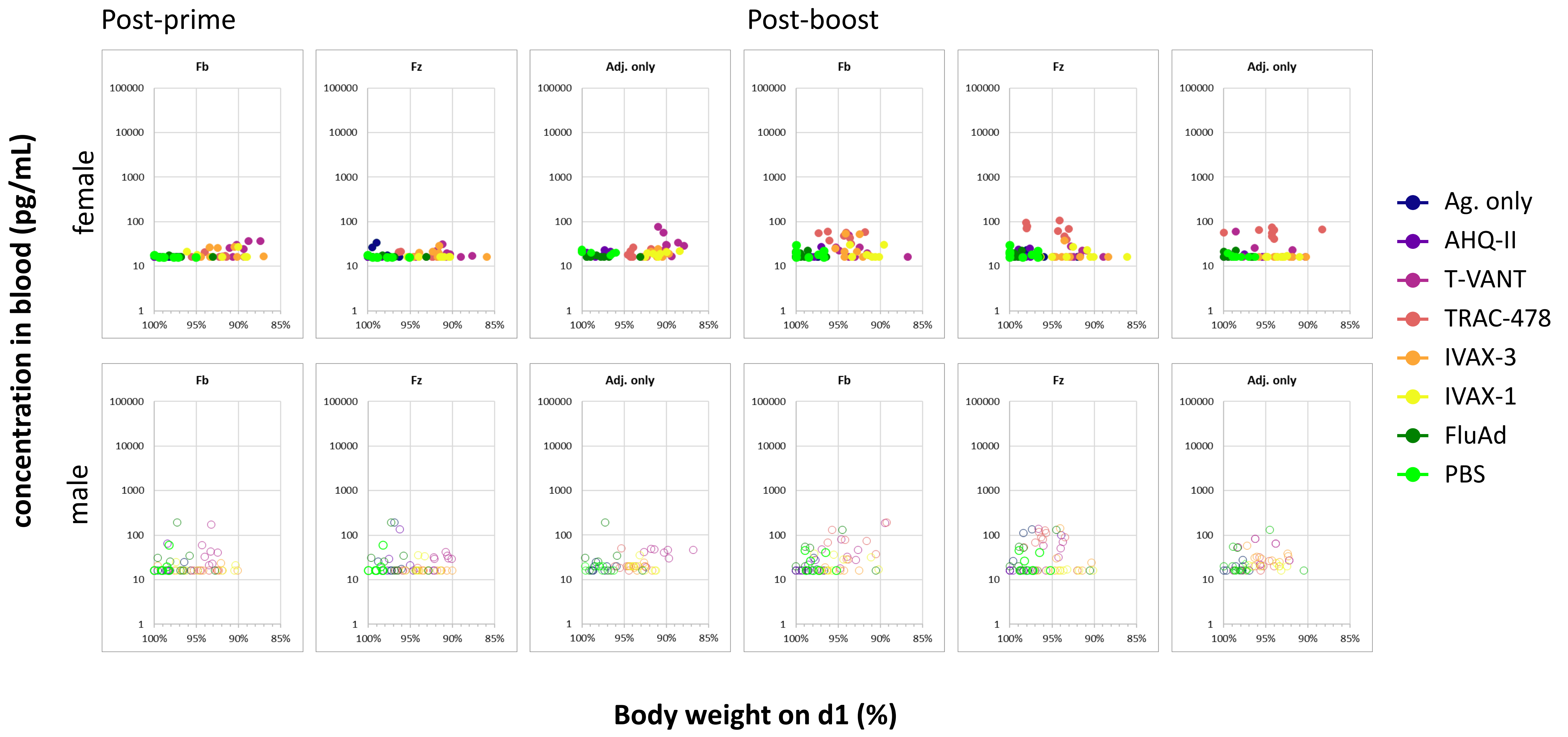

Figure S1H:

IL-10

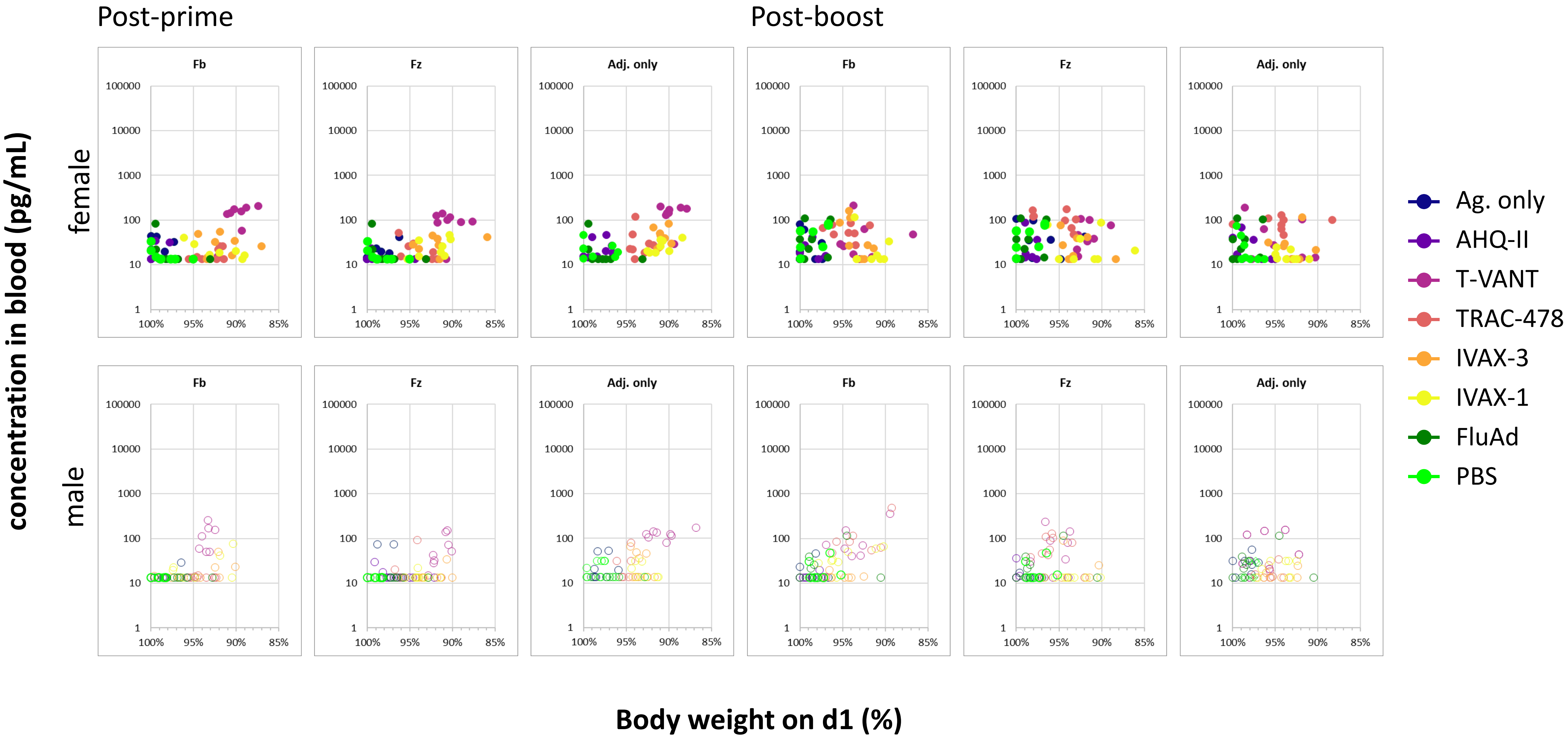

Figure S1I:
IL-6

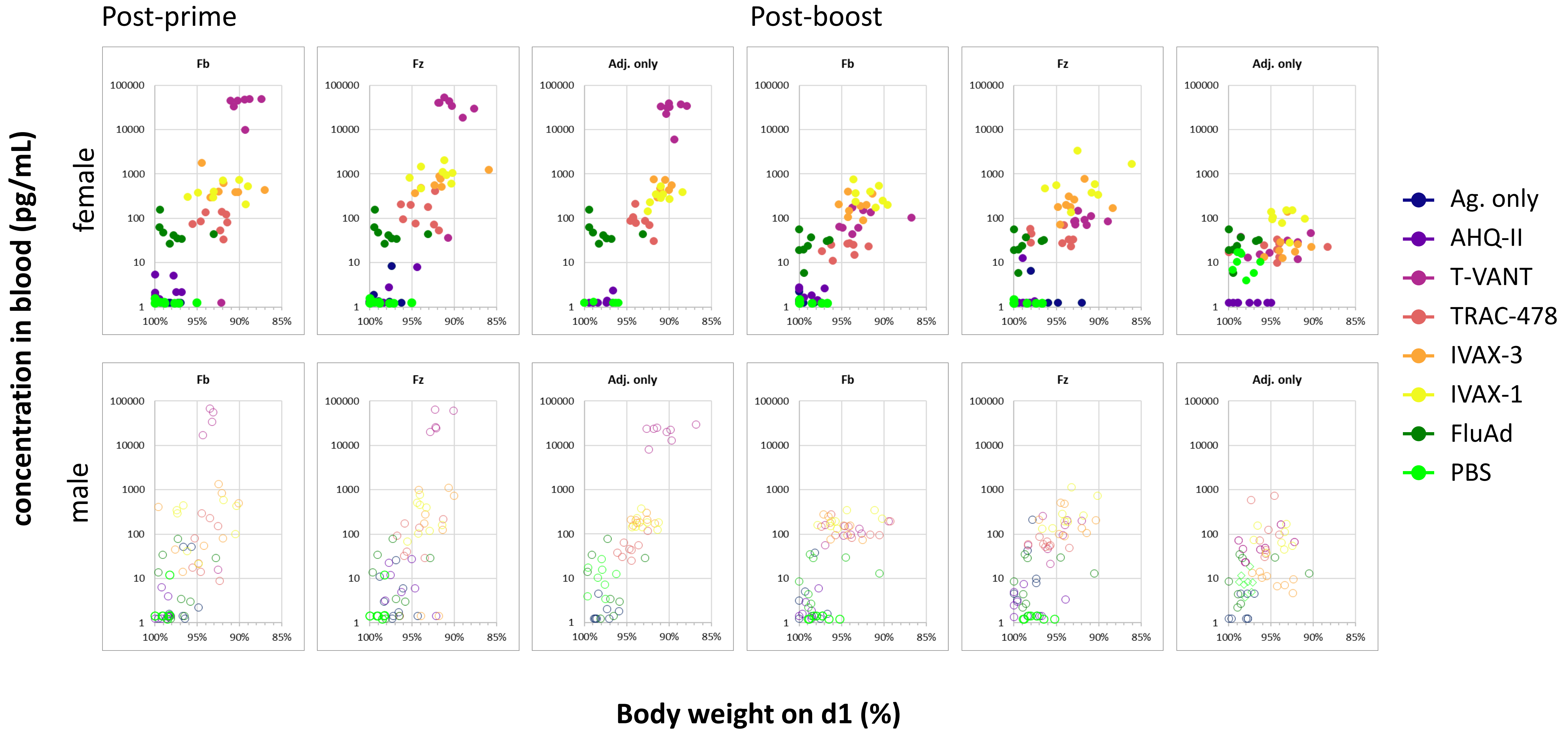

Figure S1J:
IL-27

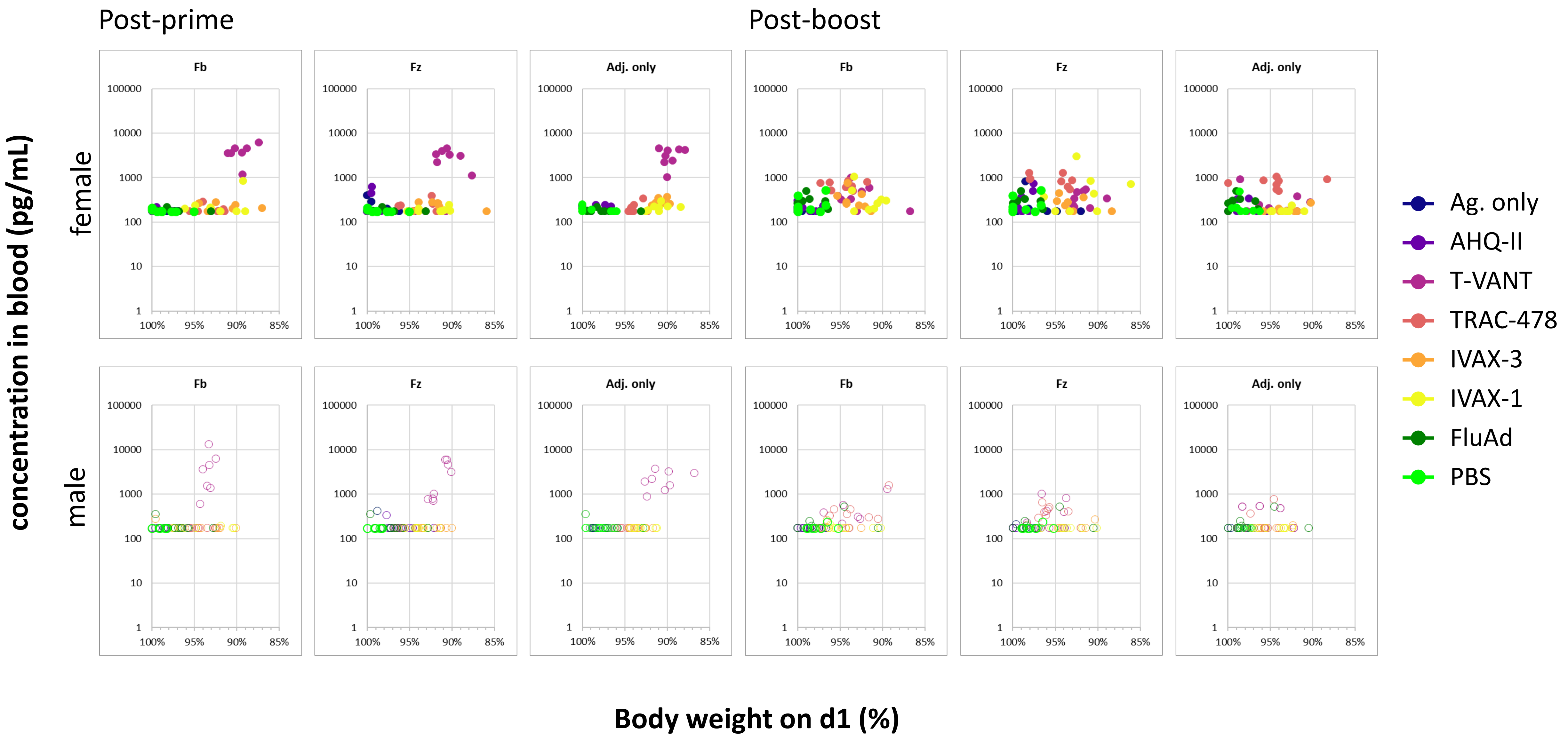

Figure S1K:

IL-17A

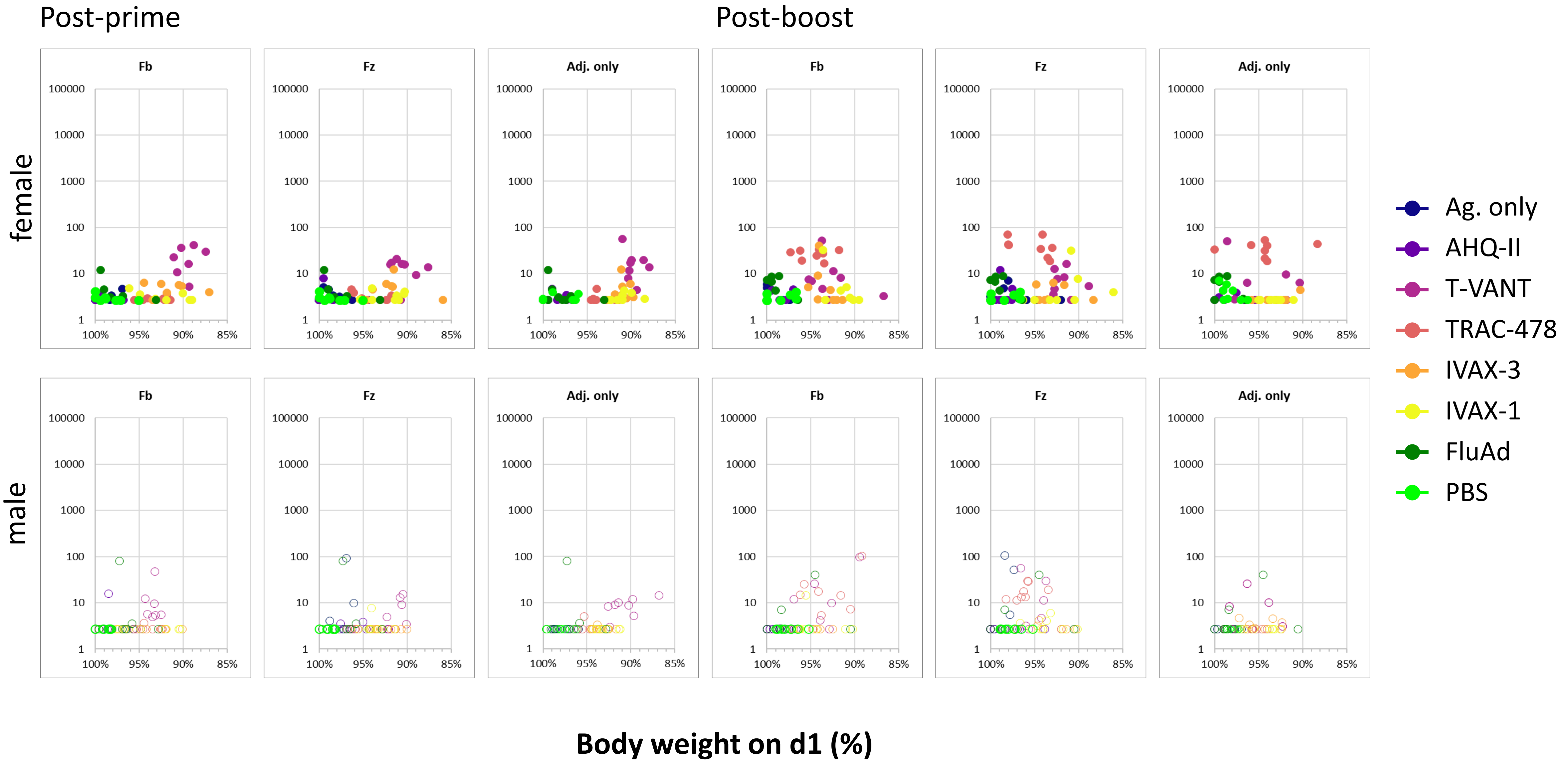

Figure S1L:
IFN-β

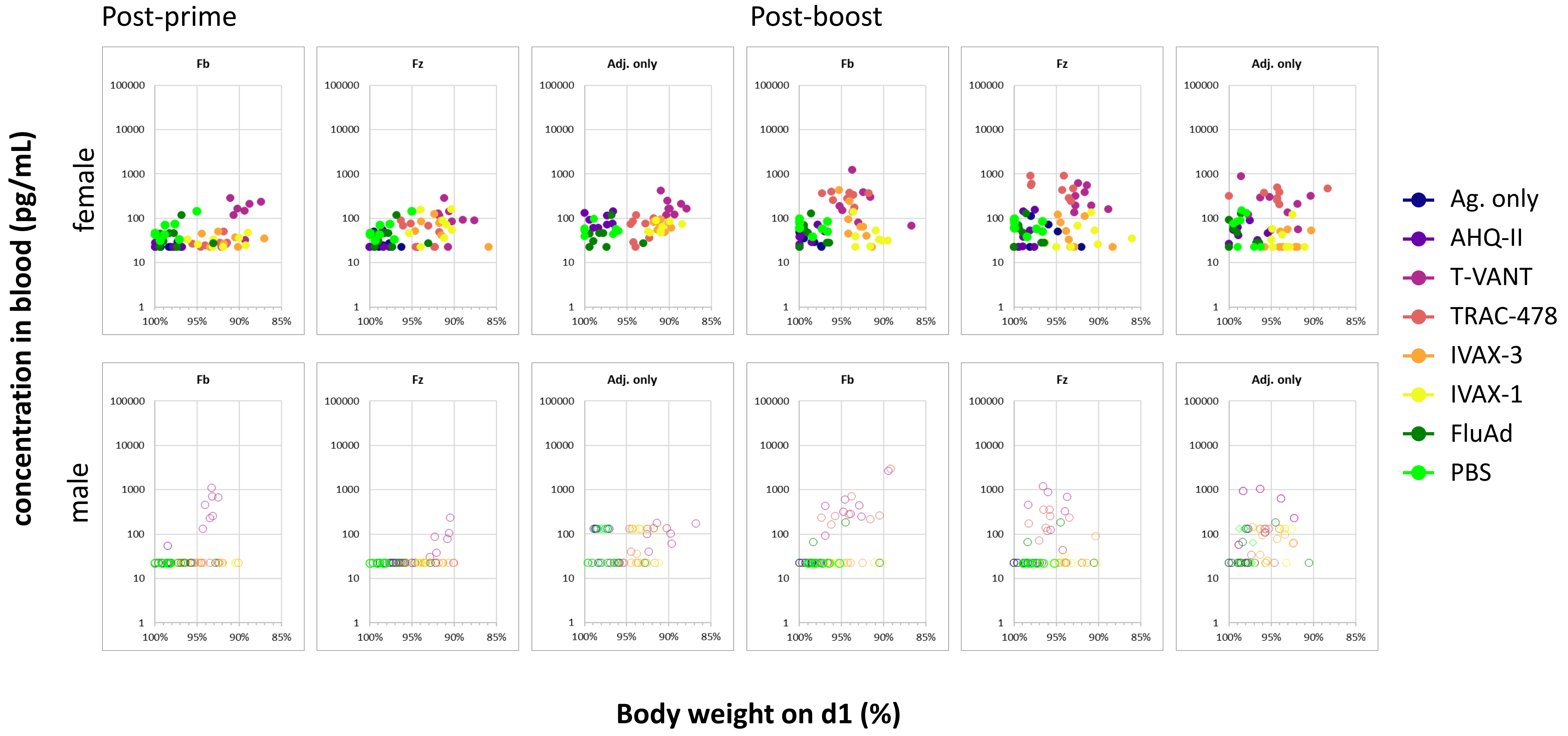

Figure S1M:
GM-CSF

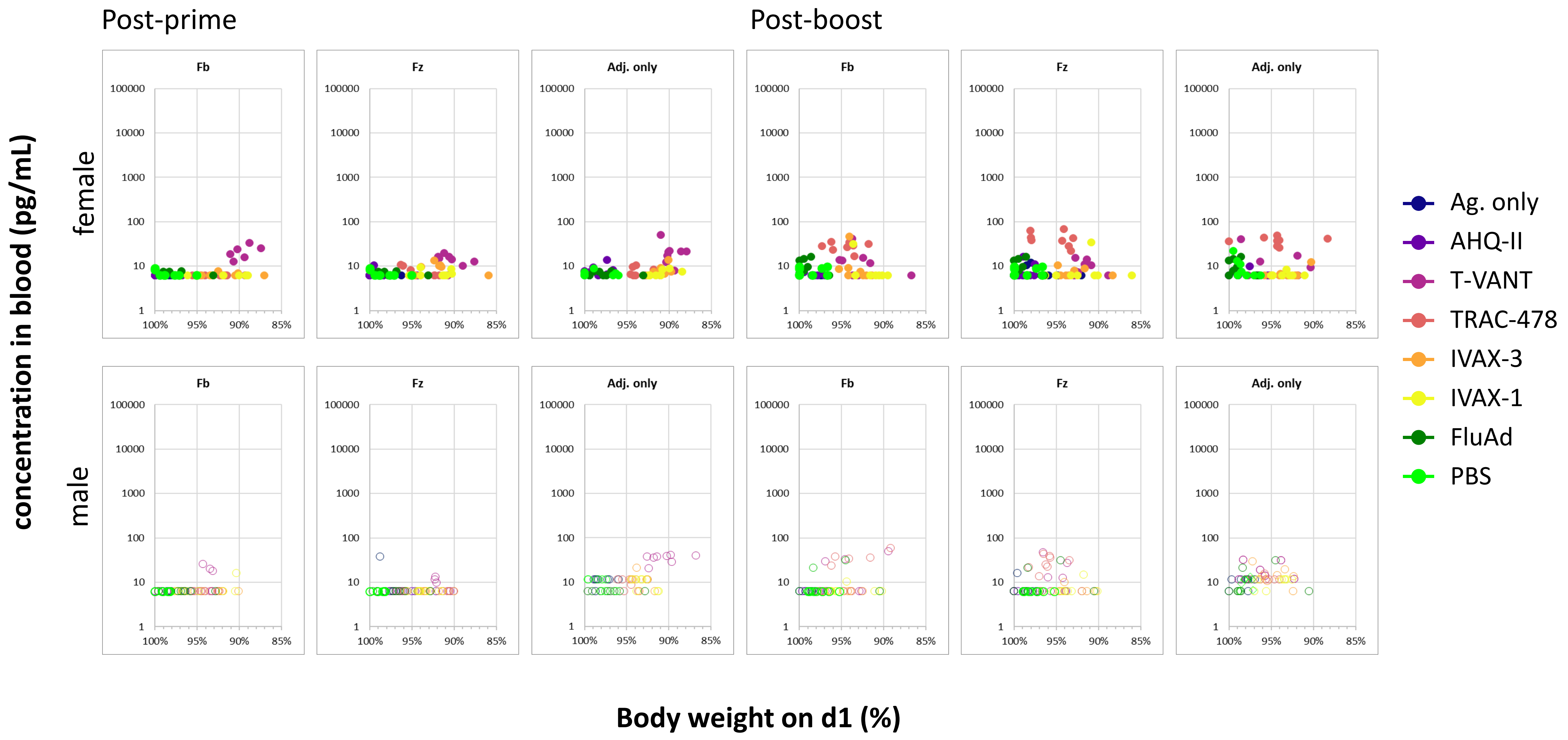

Figure S2 Panels A to F. IgG responses determined by protein microarray. Mice were administered low-dose seasonal vaccines in different adjuvants and serological assays performed on plasma collected on d28 (2 weeks post-boost). Each panel shows normalized protein microarray IgG1 and IgG2c signals to different antigens on the array as follows. **A)** 2022/2023 vaccine antigens; **B)** other H1 variants (homosubtypic cross-reactivity); **C)** other H3 variants (homosubtypic cross-reactivity); **D)** other influenza virus B (IBV) HA variants (homosubtypic cross-reactivity); **E)** variants of HA subtypes other than H1, H3 and IBV HAs (heterosubtypic cross-reactivity); **F)** nucleoproteins from different H1N1, H3N2 and IBV subtypes. MFI, mean fluorescence intensity. Statistical significance determined by comparing vaccine groups against Ag. only control groups, or PBS in the case of FluAd, using ordinary one-way ANOVA. ****, $P<0.0001$; ***, $P<0.001$; **, $P<0.01$; *, $P<0.05$; others are non-significant

Figure S2A:
Vaccine HAs

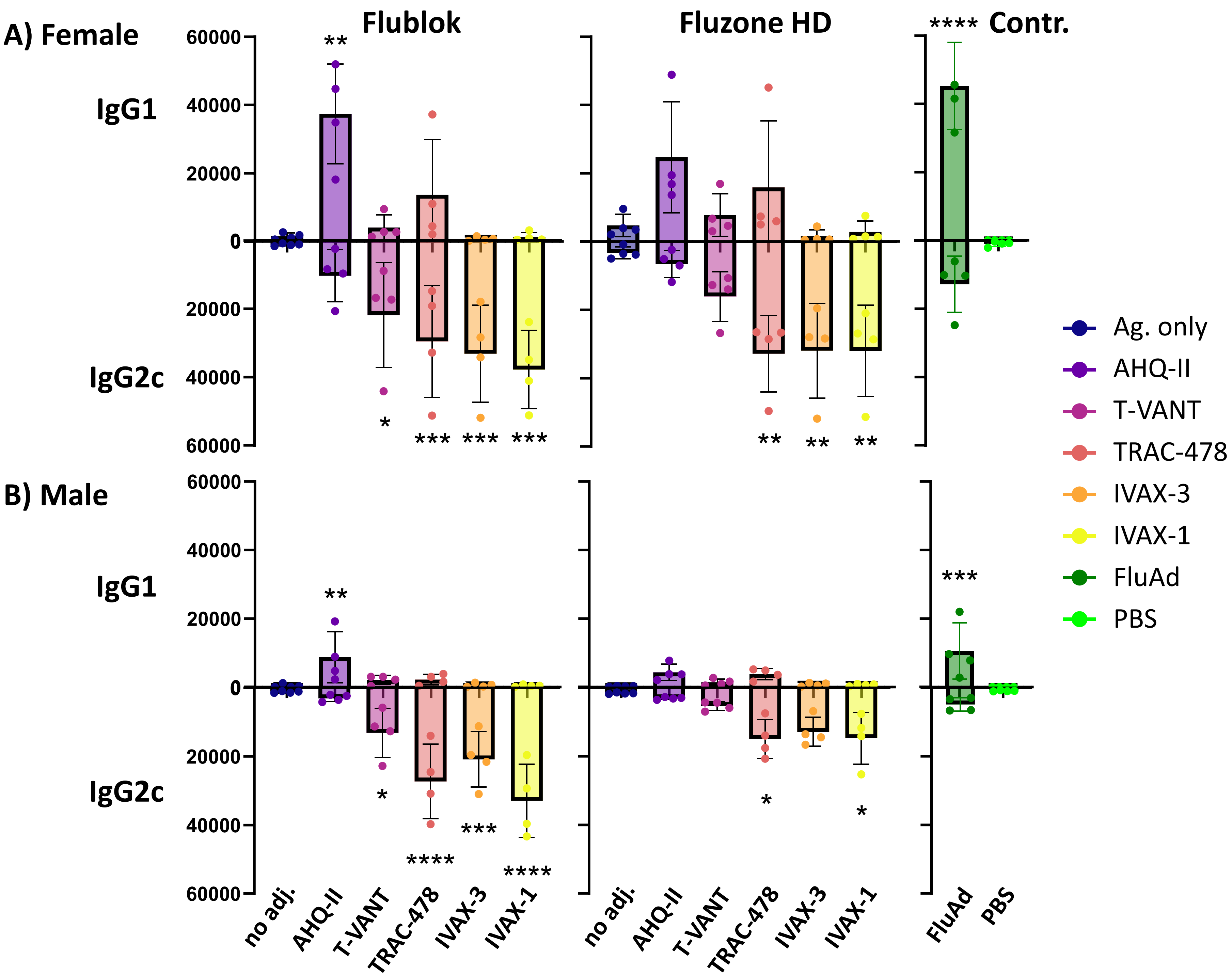

Figure S2B:
Homosubtypic H1 variants

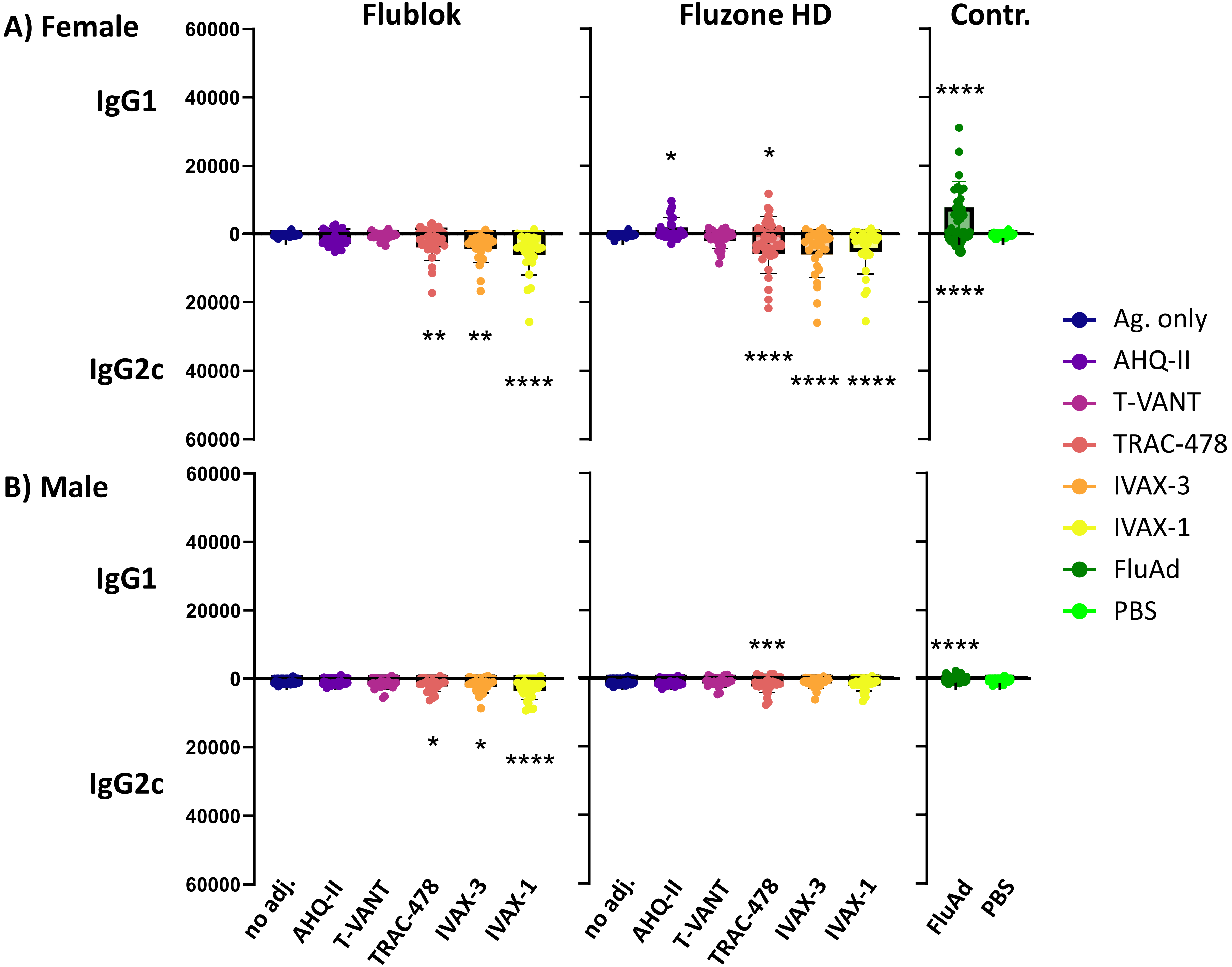

Figure S2C:
Homosubtypic H3 variants

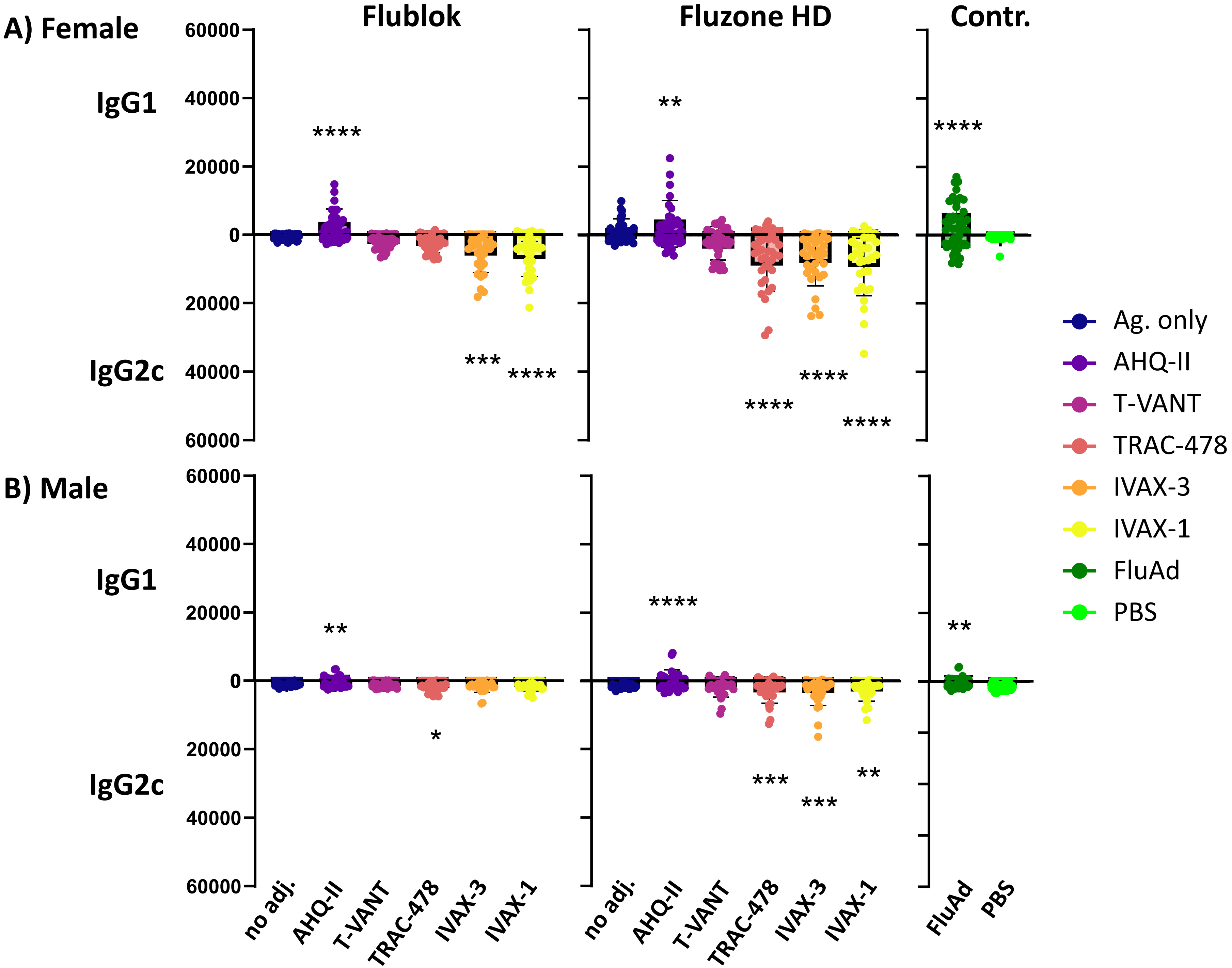

Figure S2D:
Influenza B HA variants

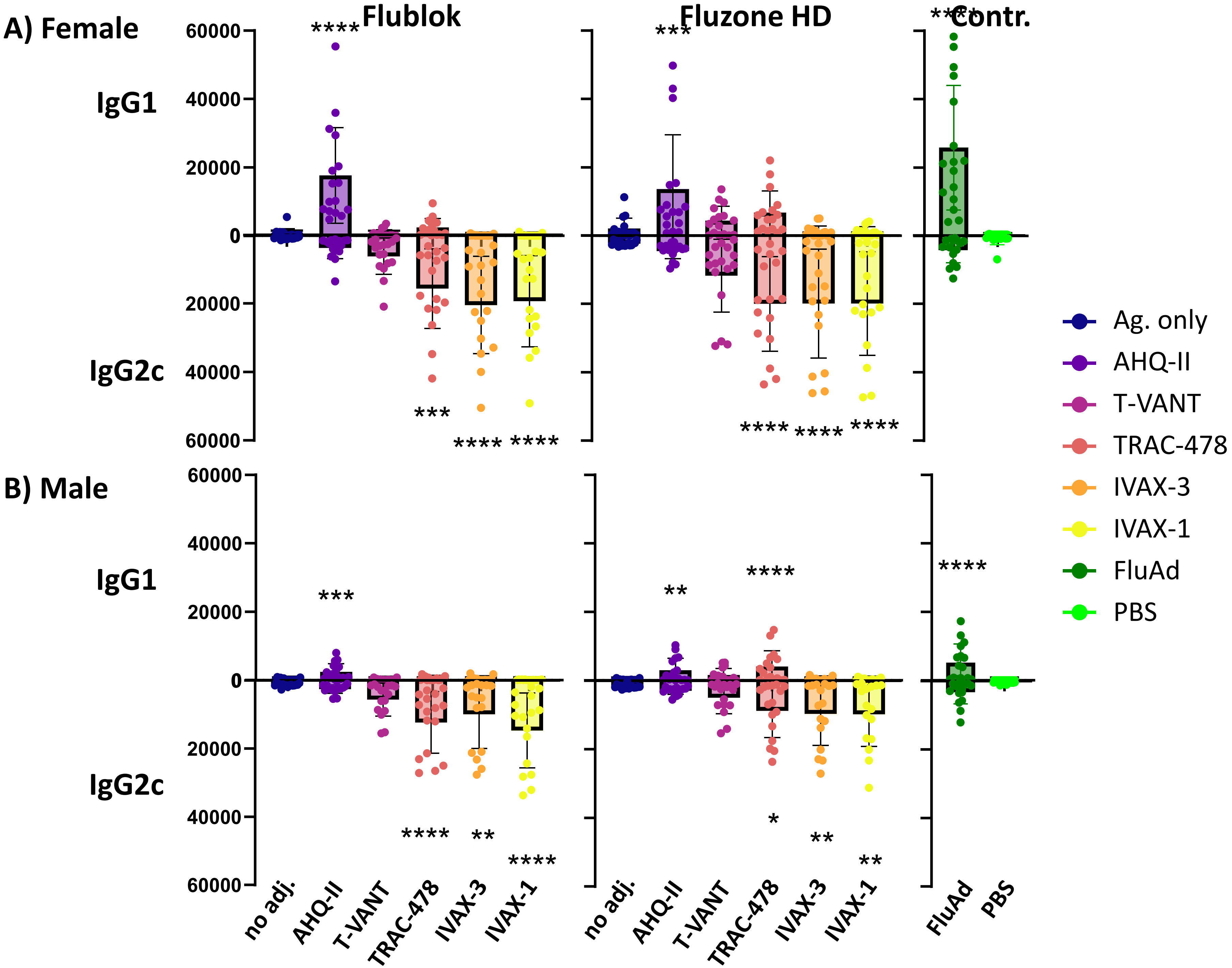

Figure S2E:
Heterosubtypic HA variants

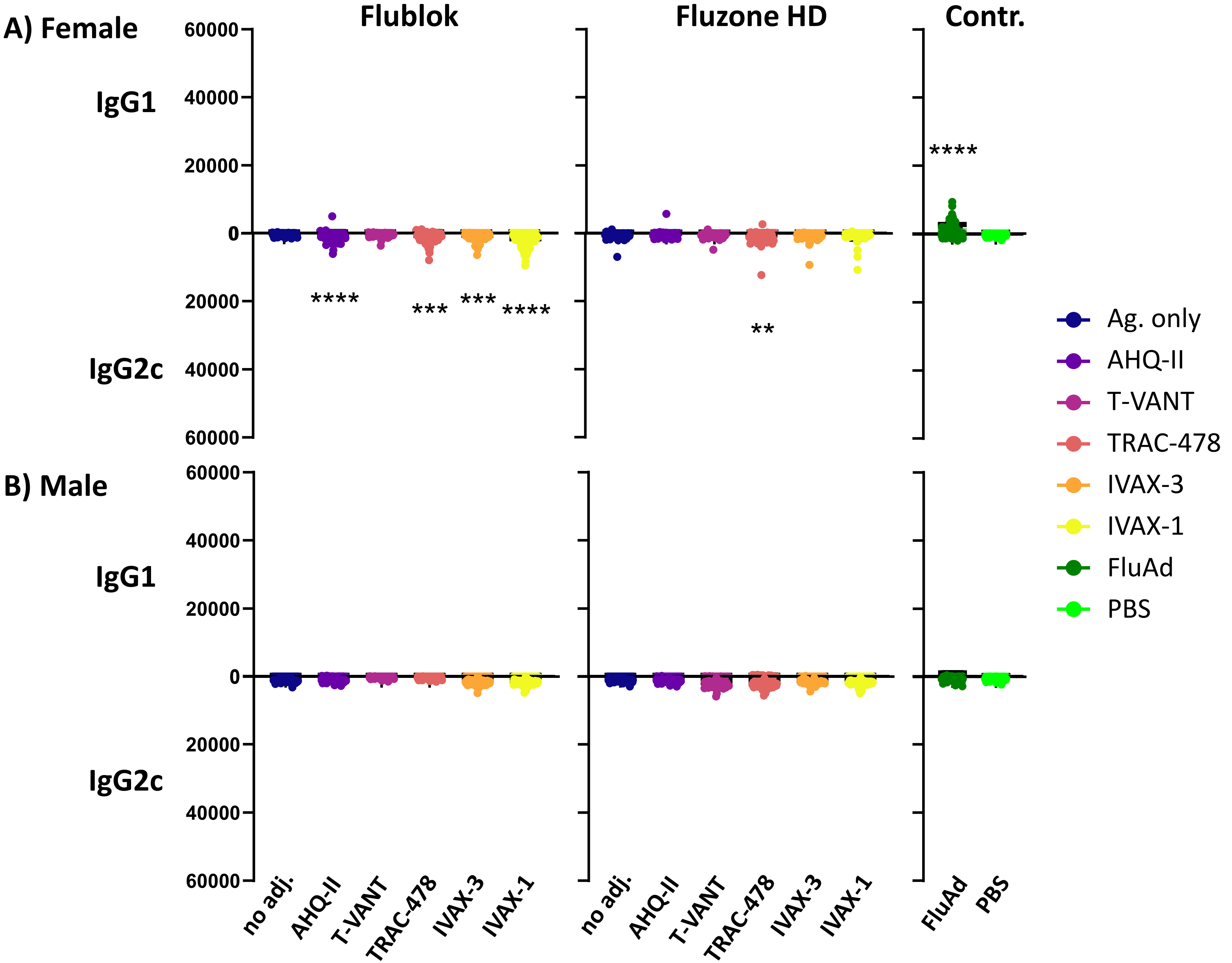

Figure S2F:
NP

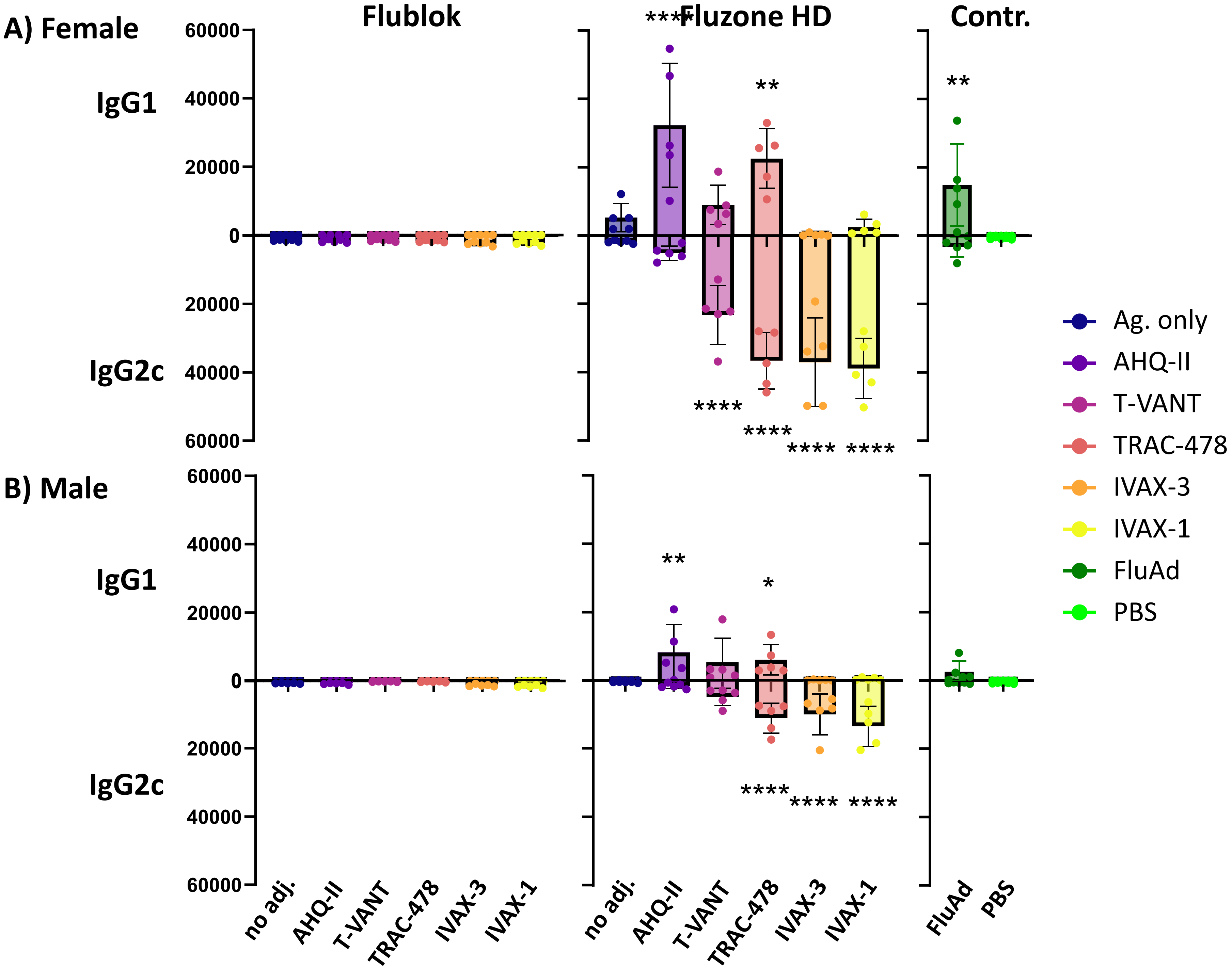

Supplementary Fig. S3. Relationship between d28 nAb titers by microneutralization (MN) and hemagglutination inhibition (HAI) assays. Each symbol represents an individual mouse. Solid circles, females; open circles, males

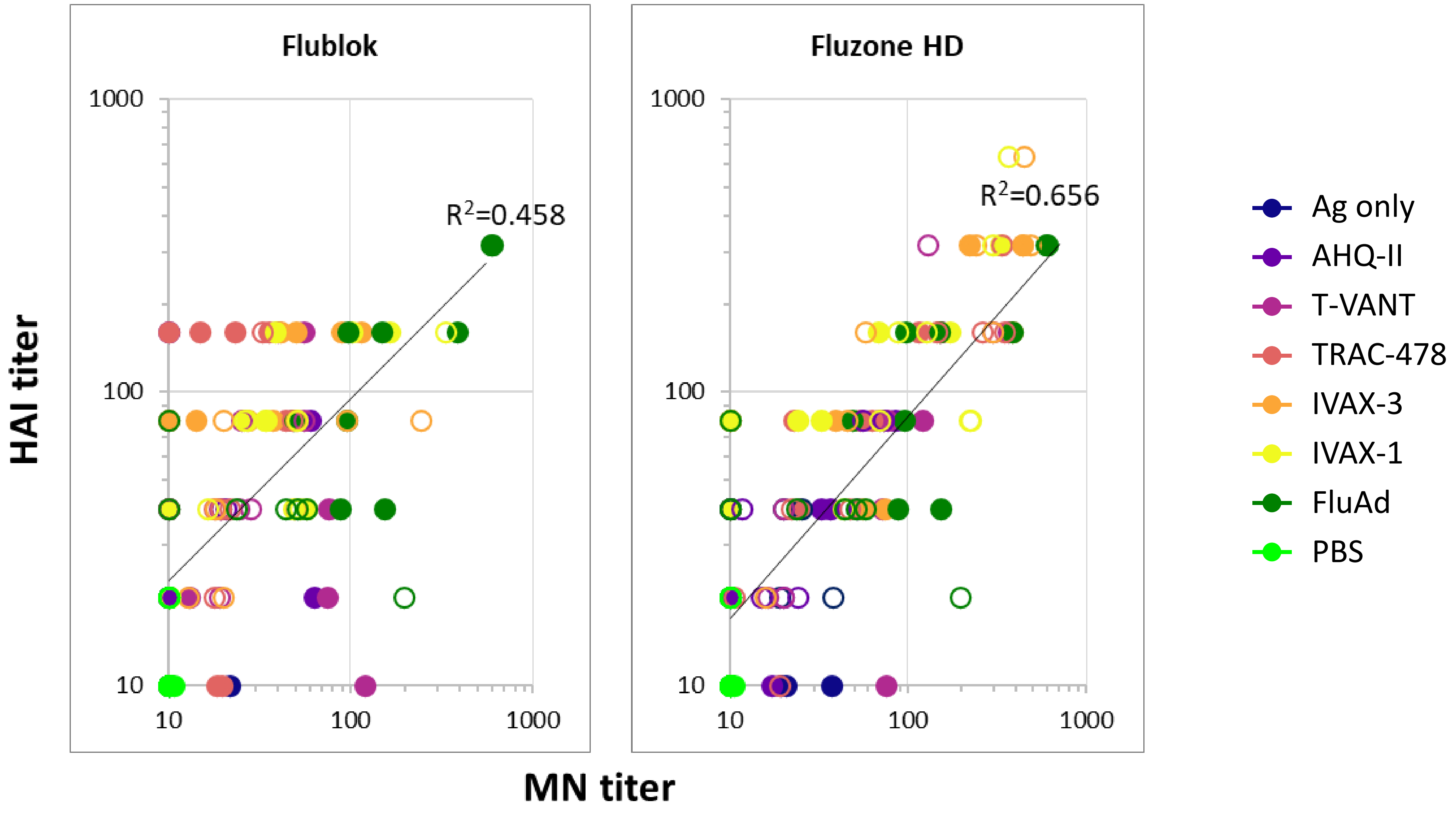

Figure S4. Scatter plots of nAb titers on d28 and maximum weight loss after H1N1 challenge. A) MN titers vs. maximum weight loss; **B)** HAI titers vs. maximum weight loss. Each symbol is an individual mouse. Solid symbols, females; open symbols, males; horizontal hashed line, 1:40 titer cut-off; left of vertical green line, <5% loss of body weigh (protected with no morbidity); between vertical green and red lines, 5-20% loss of body weight (protected with morbidity); right of vertical red line, not protected (mortality). Fb, Flublok; Fz, Fluzone HD.

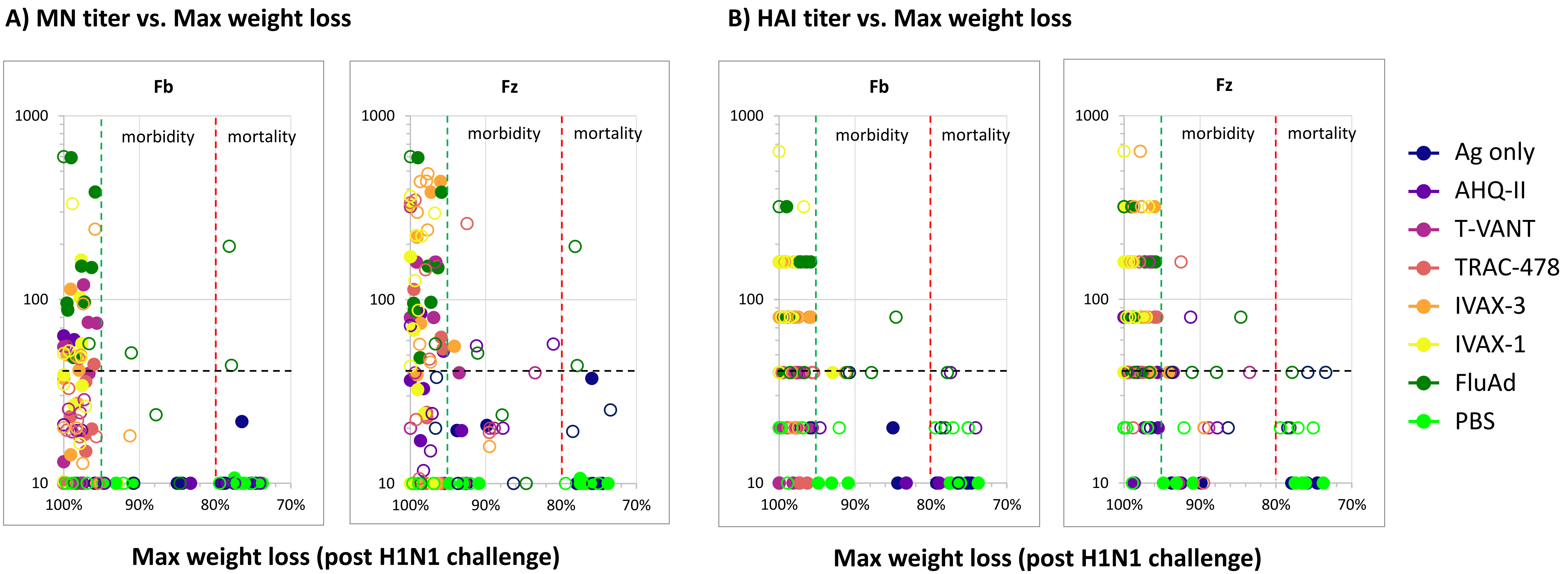

Figure S5 Scatter plots of nAb titers on d28 and maximum weight loss after H1N1 challenge separated by adjuvant.

A) MN titers vs. maximum weight loss; **B)** HAI titers vs. maximum weight loss. Each symbol is an individual mouse; male and female data are pooled. The small square at bottom left denotes animals that were protected without morbidity (<5% weight loss) with a nAb titer of 1:40 or below. Orange symbols = Flublok; Blue symbols = Fluzone

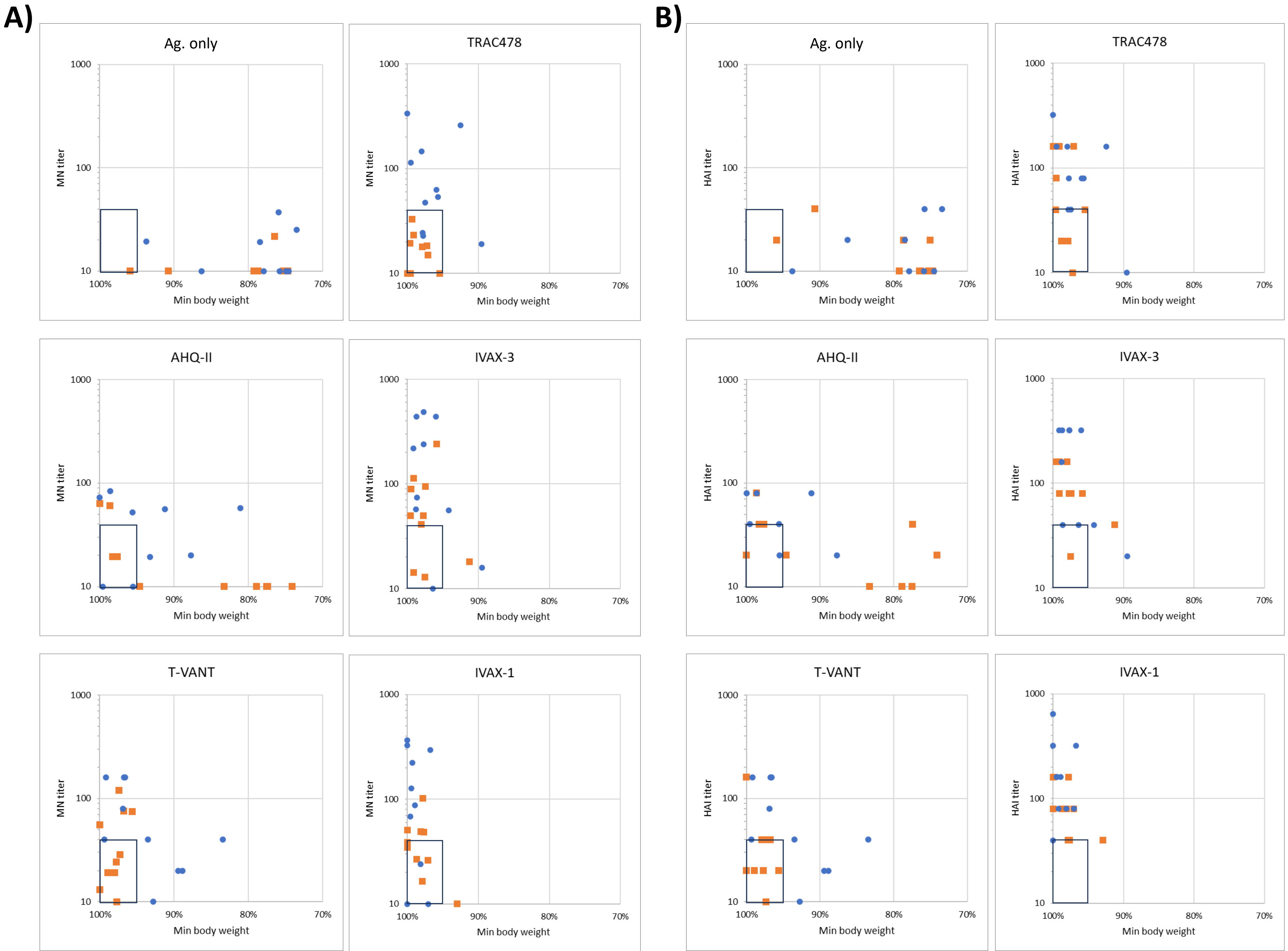

Supplementary Fig. S6

Relationship between d28 nAb titers by microneutralization (MN) and hemagglutination inhibition (HAI) assays and corresponding virus lung titers by qPCR on d3 post challenge for individual animals (females, solid symbols; males, open symbols).

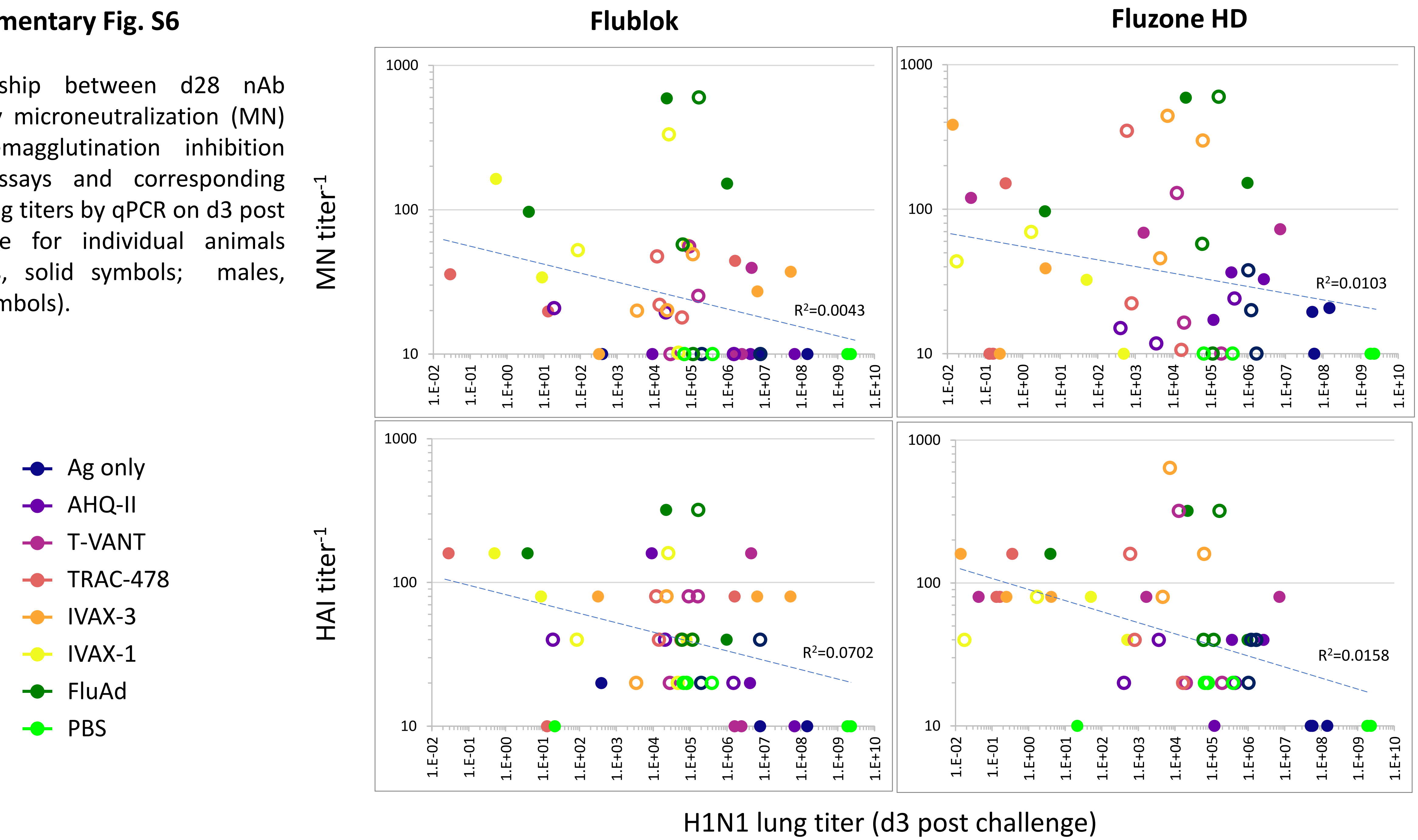

Supplementary Fig. S7. Correlation between cytokines in the blood 3h after vaccination and efficacy. Shown are bubble plots of individual cytokines in the blood at 3h after prime and boost, and corresponding efficacy for each mouse. Cytokine levels are expressed as log fold-over Ag. only controls, except for FluAd which was expressed as log fold-over PBS control ("FluAd (PBS)"). Efficacy is defined by the lowest weight seen at any time after H1N1 challenge (from Fig. 7), where <5% weight loss = protected without morbidity, 5-20% weight loss = protected with morbidity, and >20% weight loss = non-protected (animals were euthanized). Size of bubbles indicate the proportion of animals in each of the 4 conditions for each adjuvant. Abbreviations: Fb, Flublok; Fz, Fluzone HD, FC, fold-over Ag. only control.

Figure S7A

IL-23

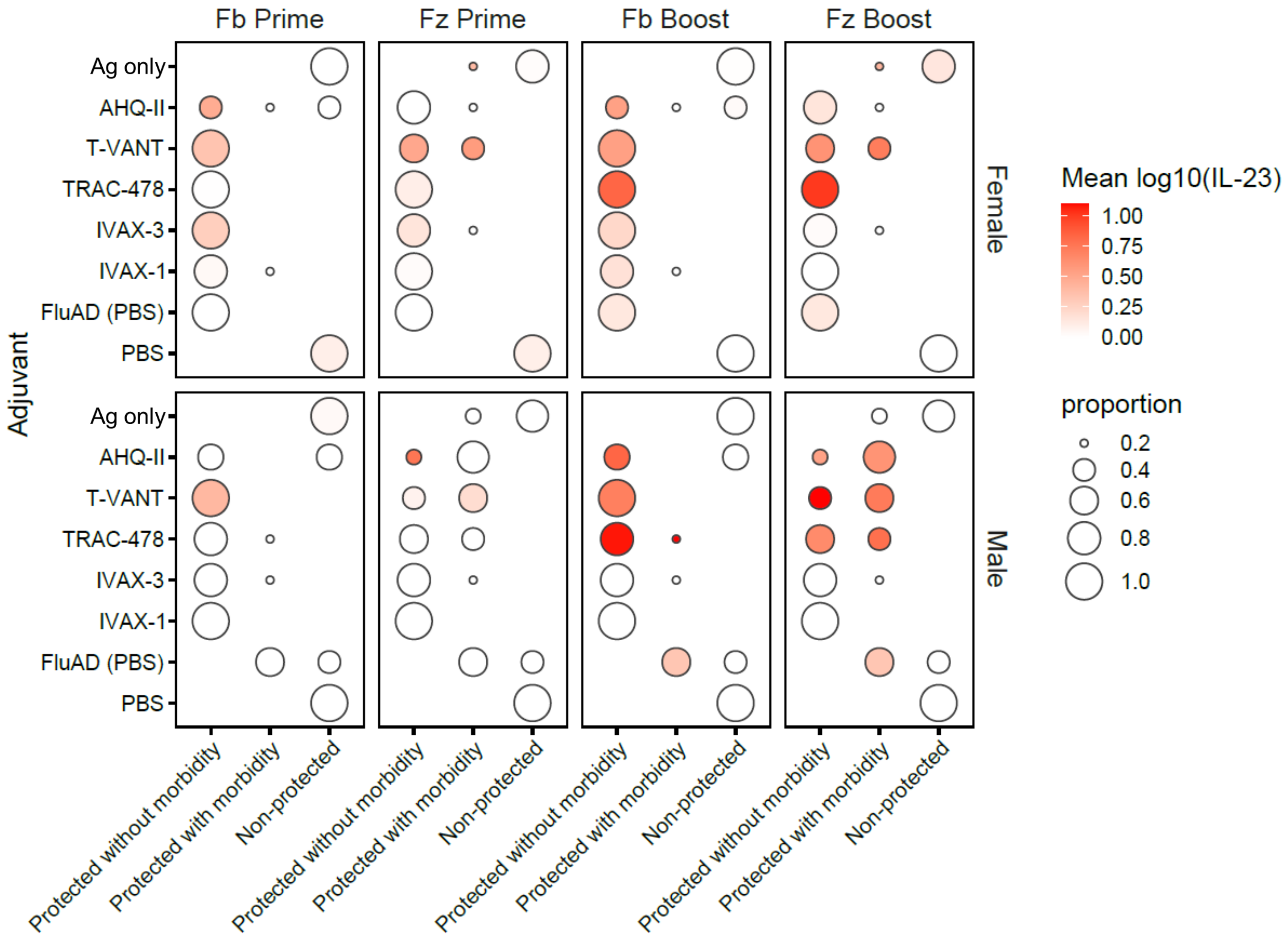

Figure S7B

IL-1α

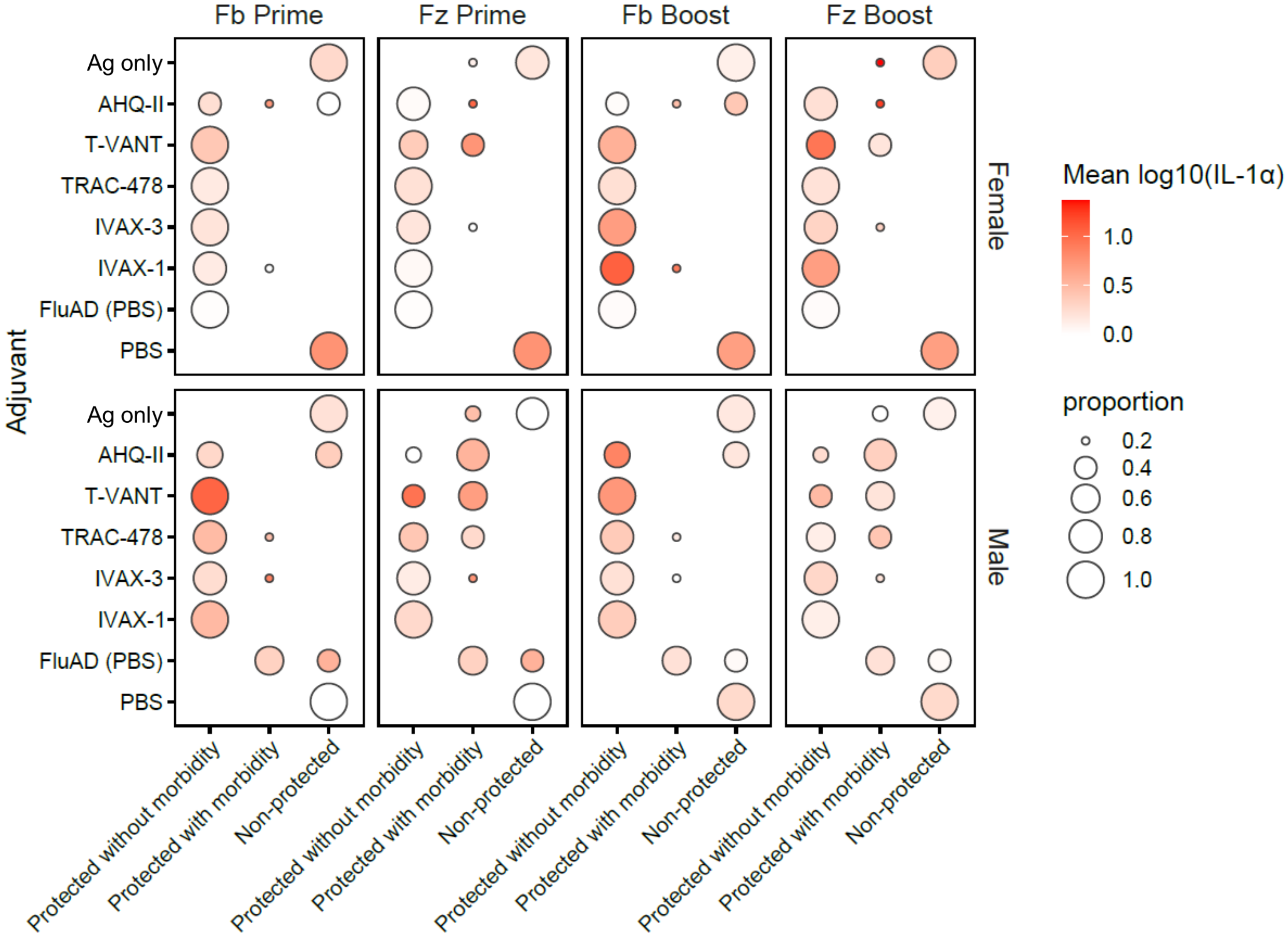

Figure S7C

IFN-γ

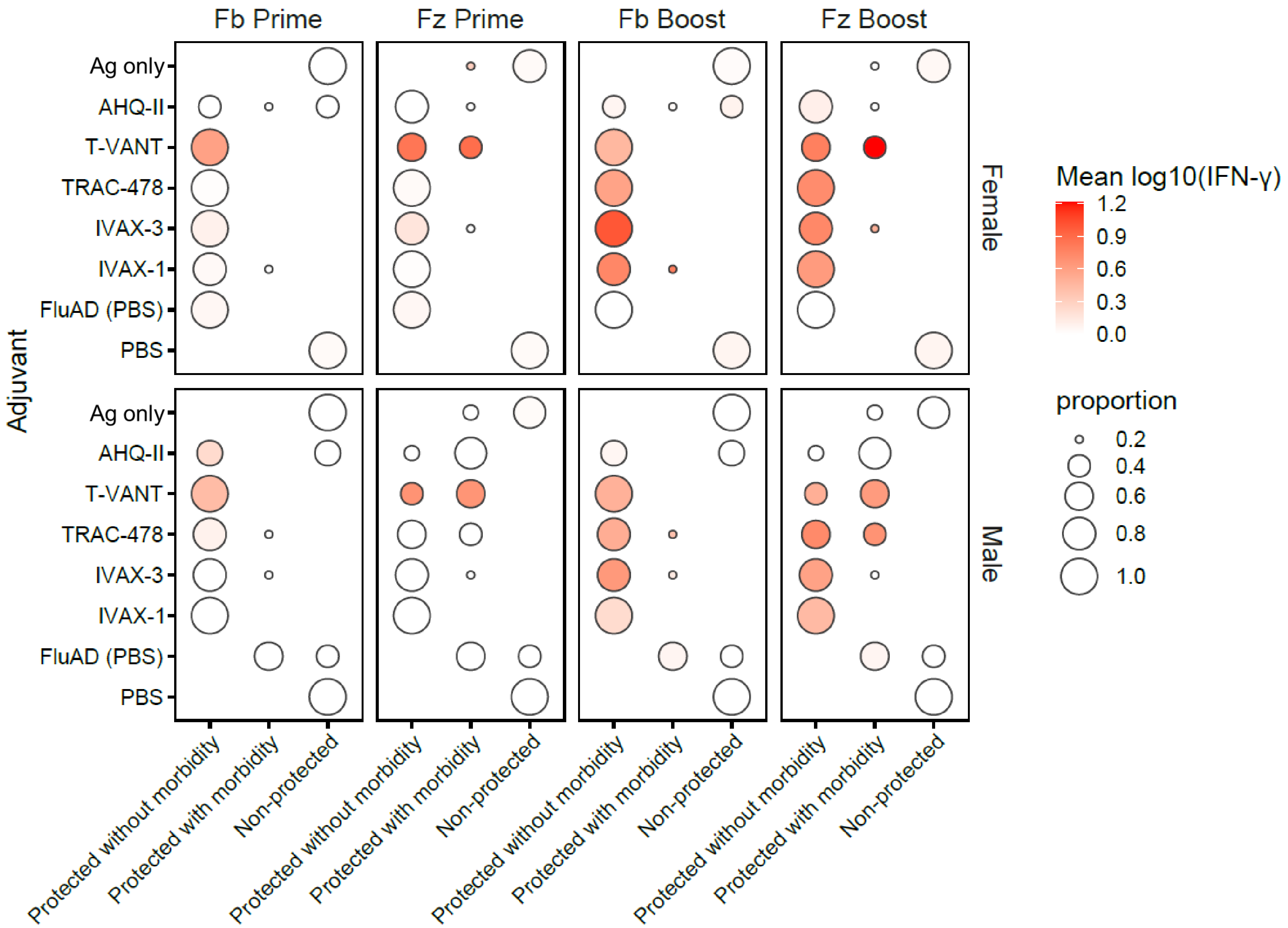

Figure S7D

TNF-α

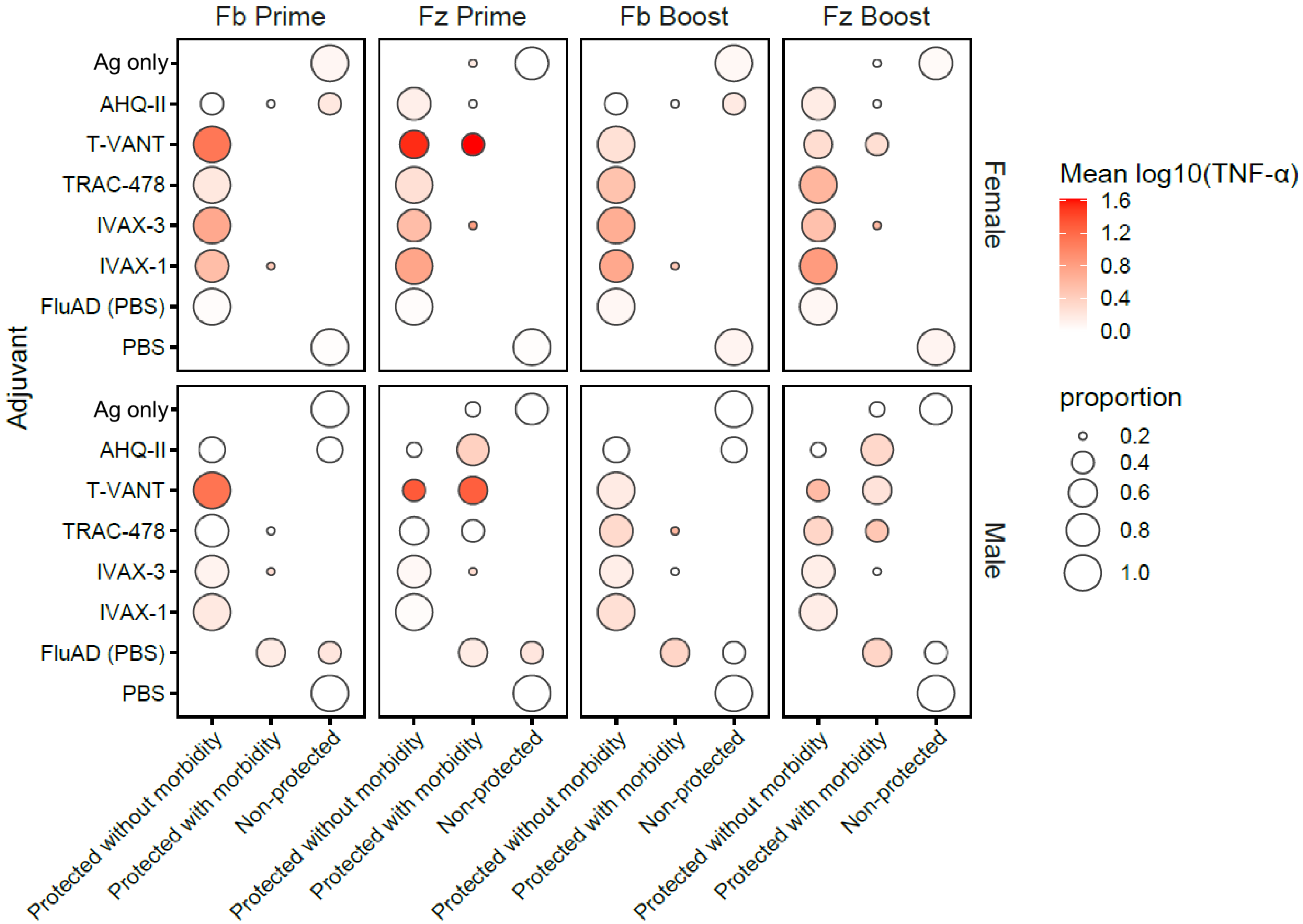

Figure S7E

MCP-1/CCL2

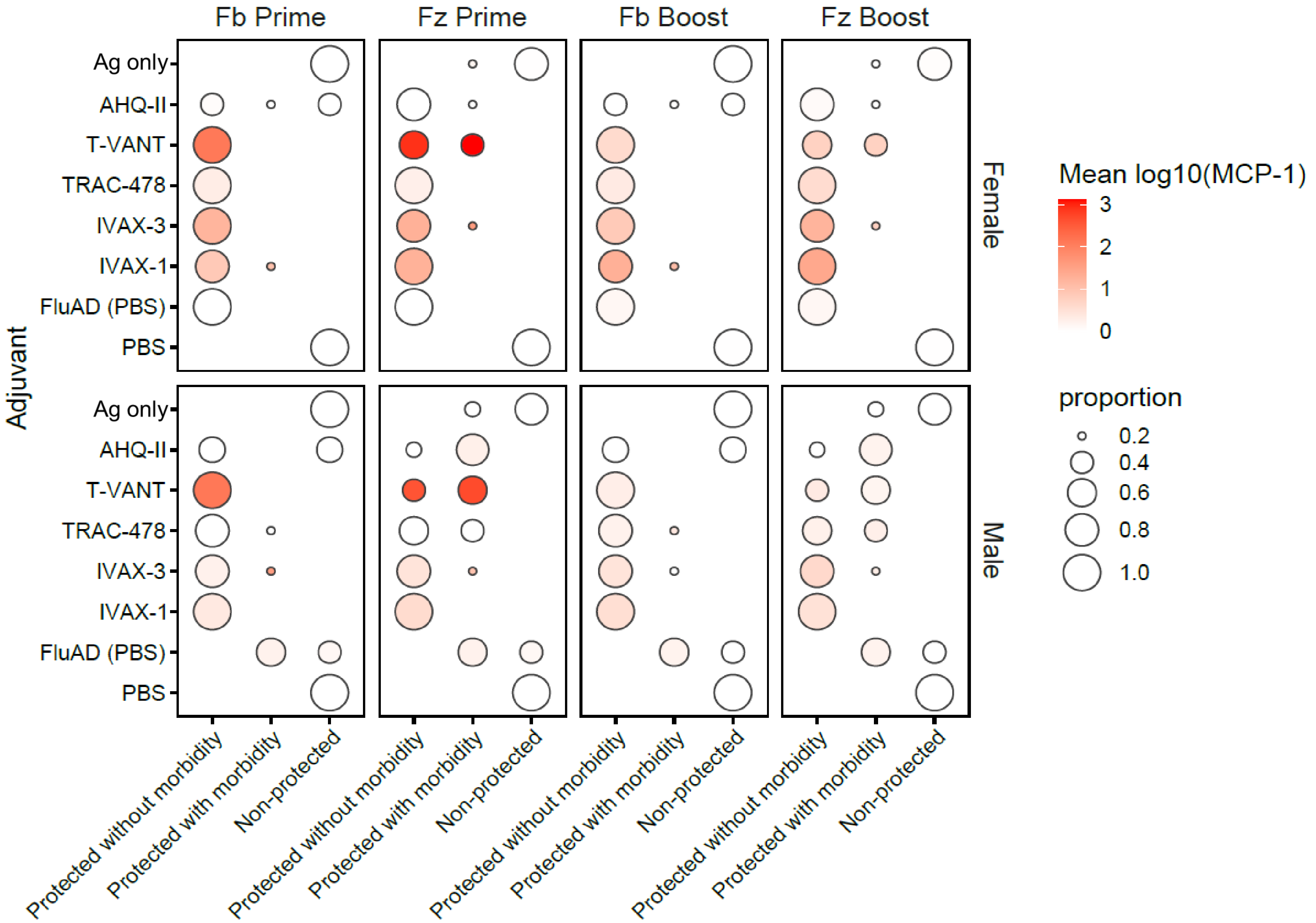

Figure S7F

IL-12p70

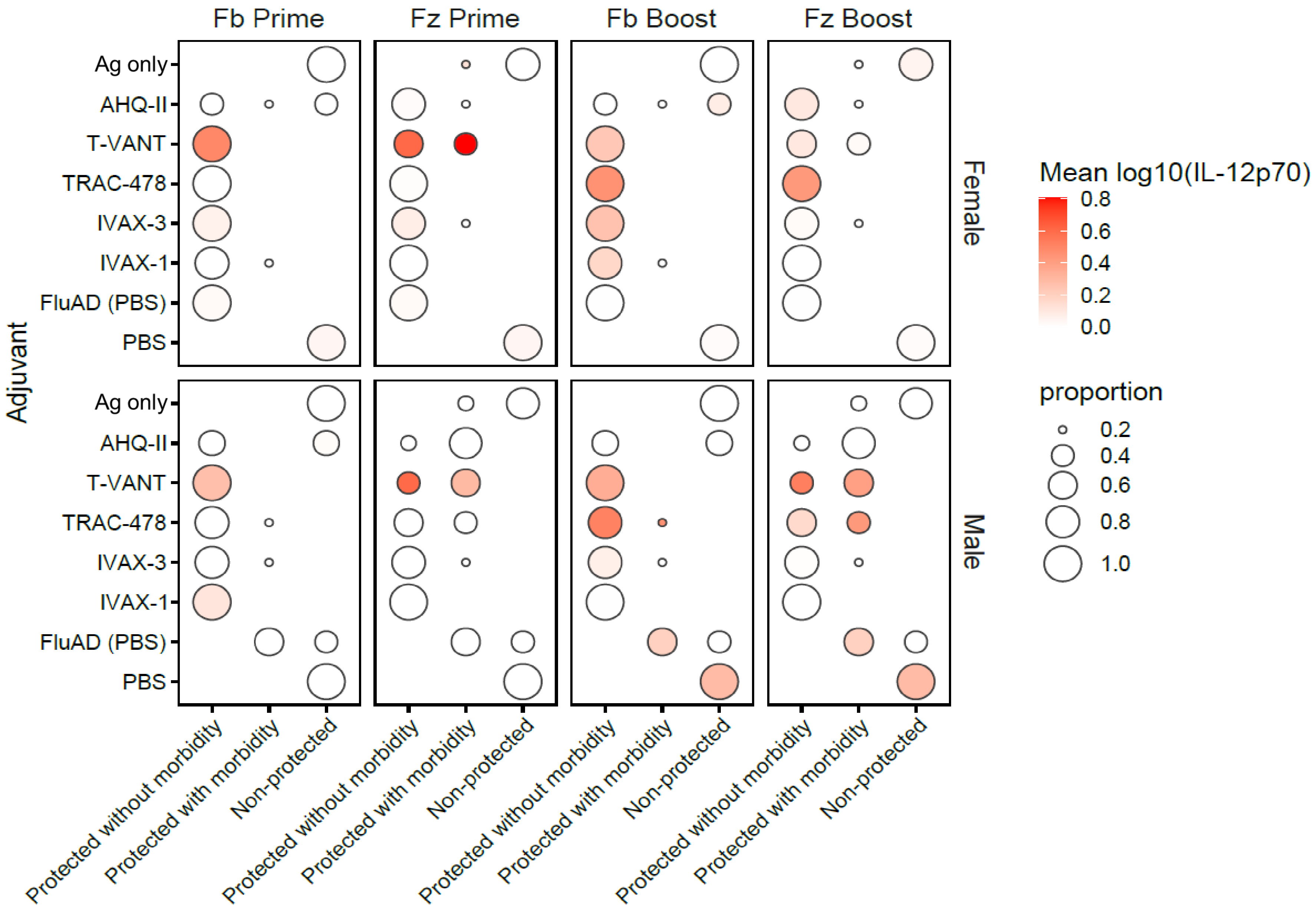

Figure S7G

IL-1β

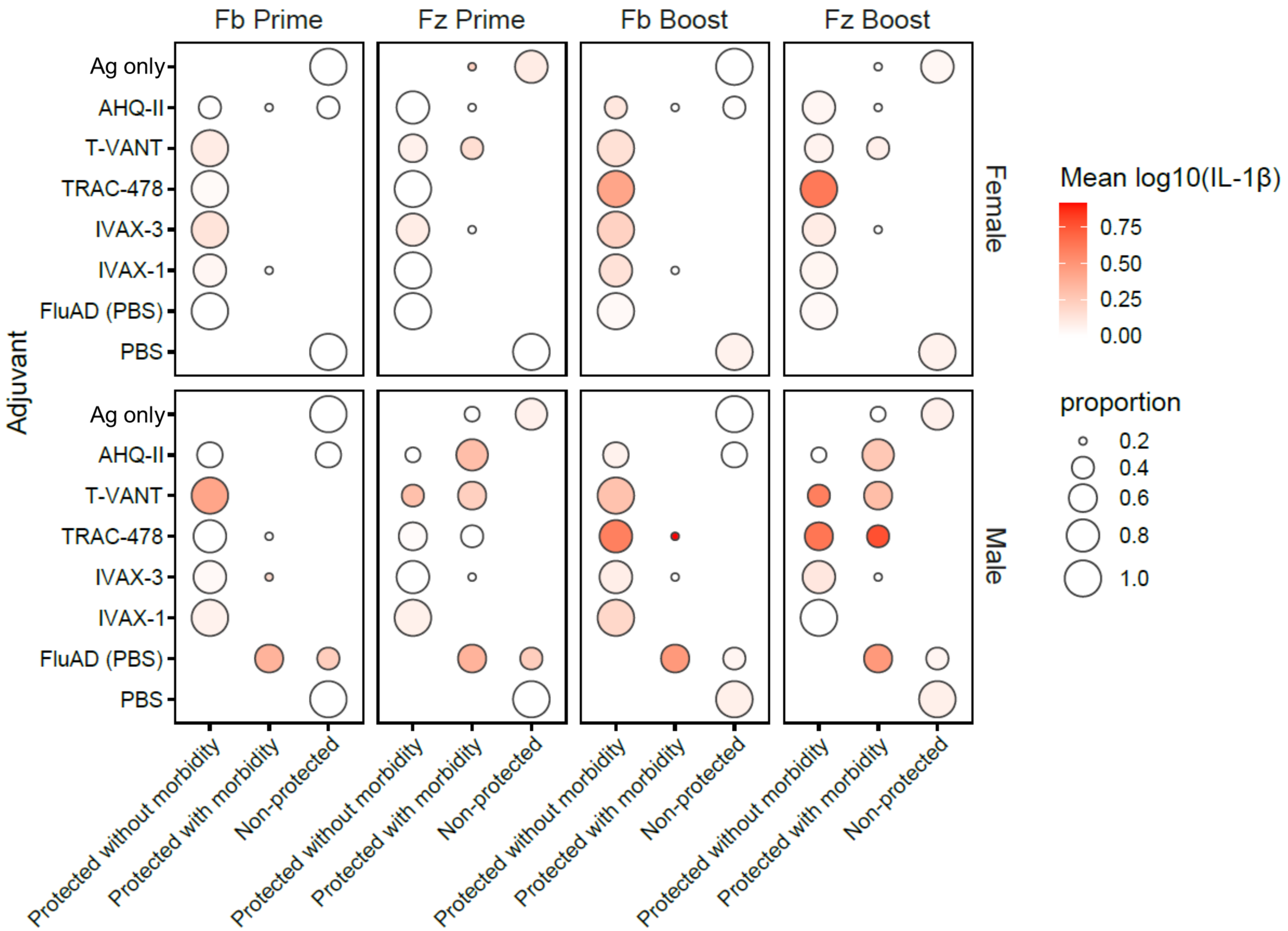

Figure S7H
IL-10

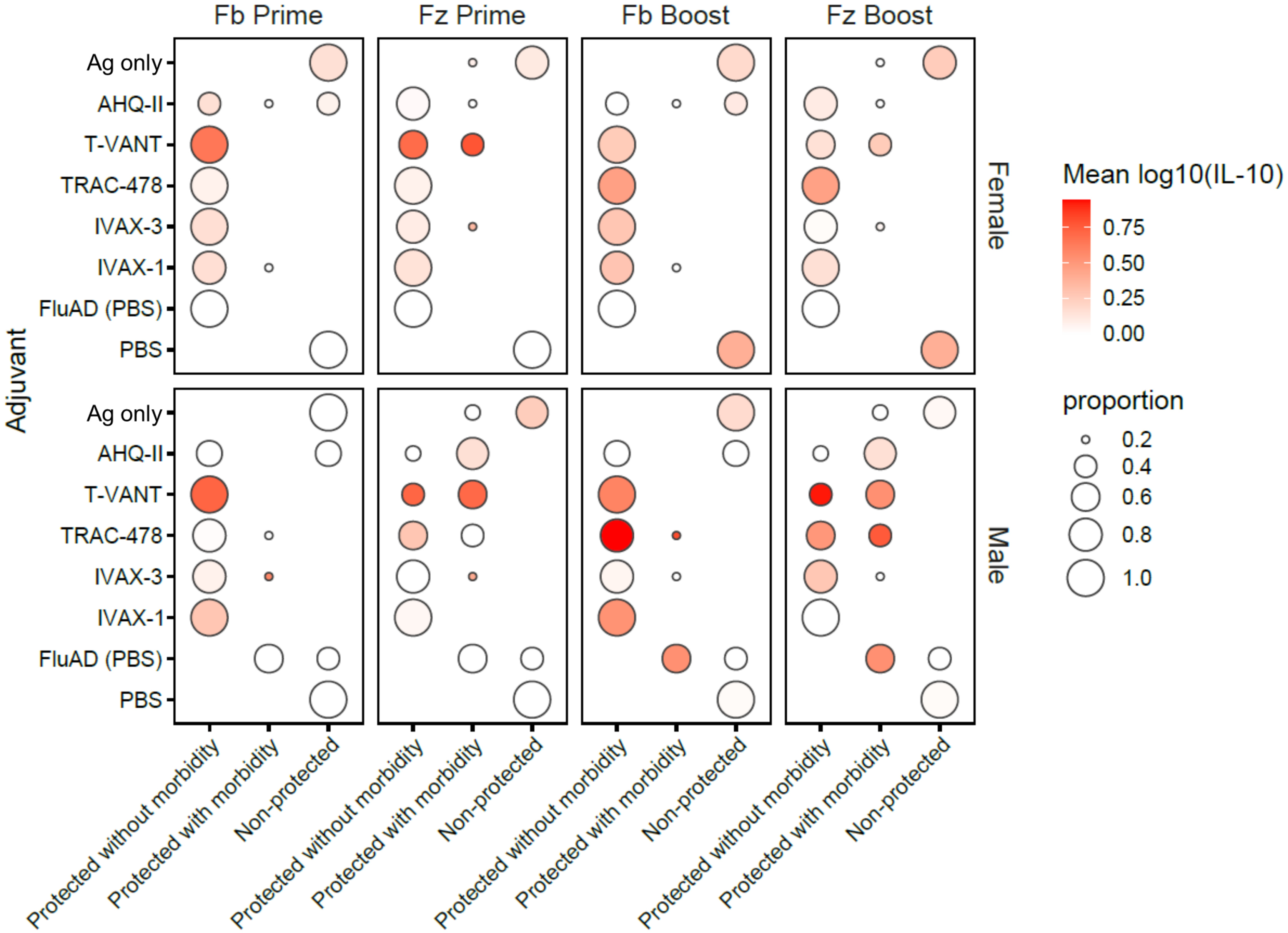

Figure S7I
IL-6

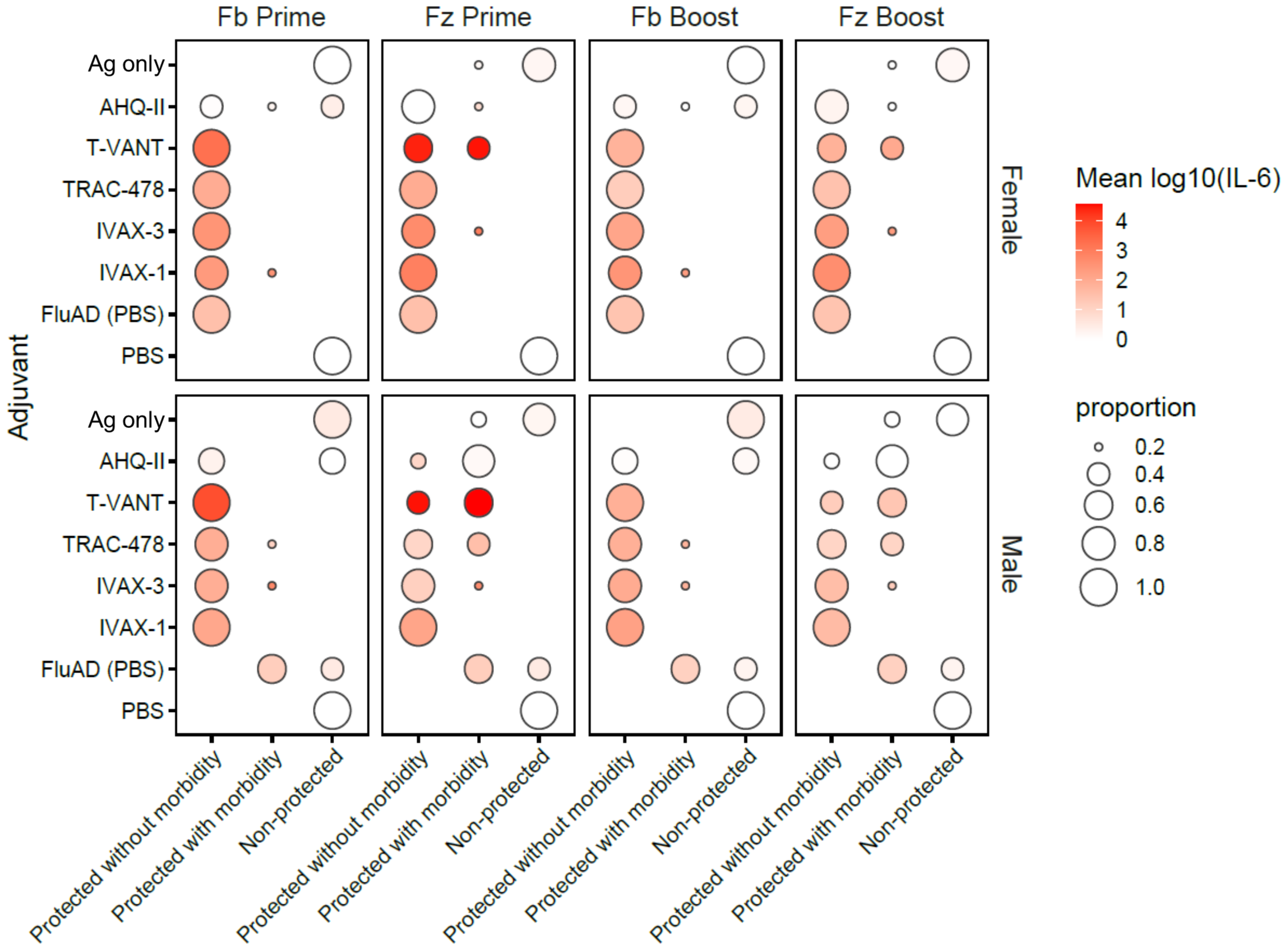

Figure S7J
IL-27

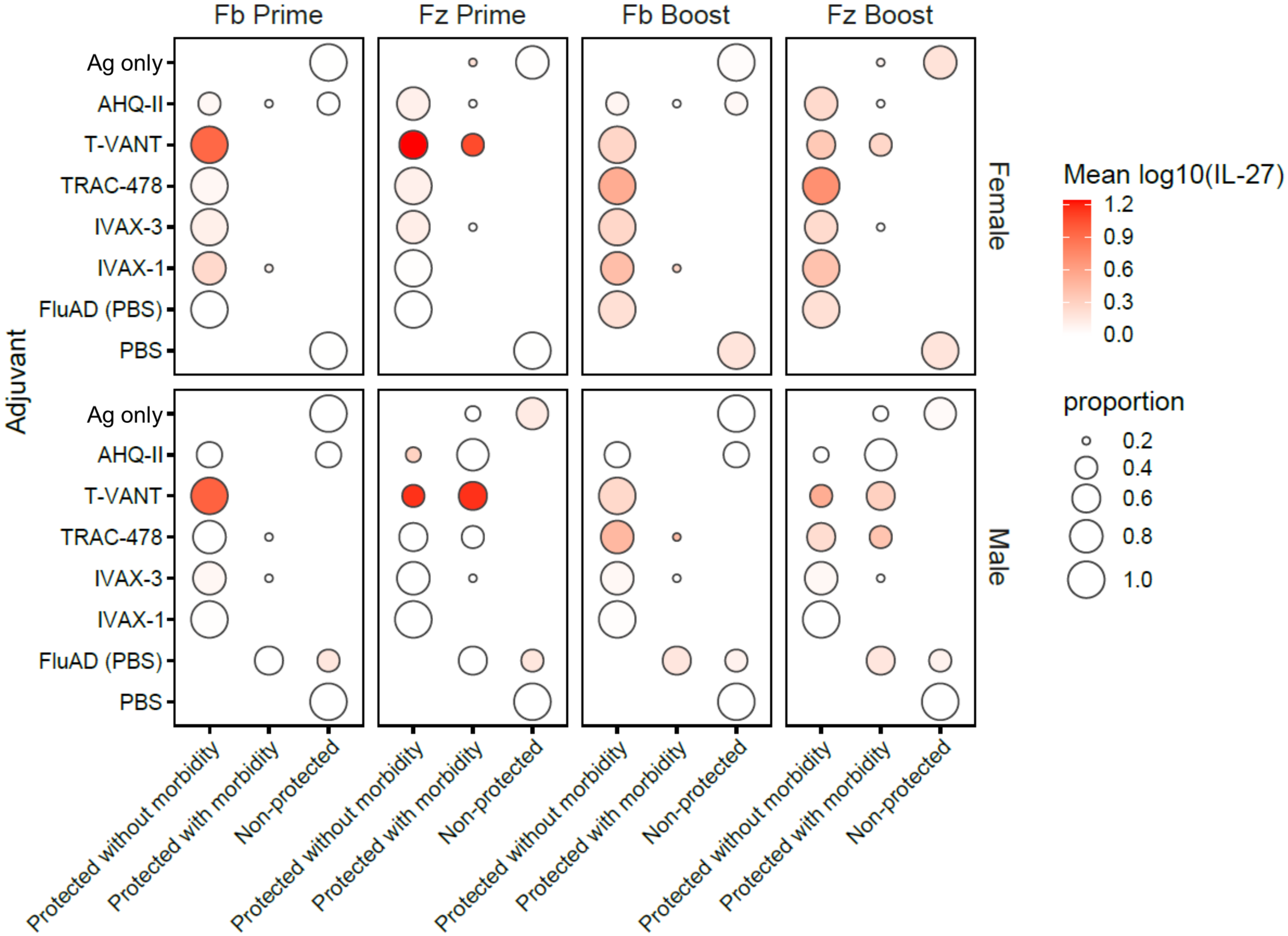

Figure S7K

IL-17A

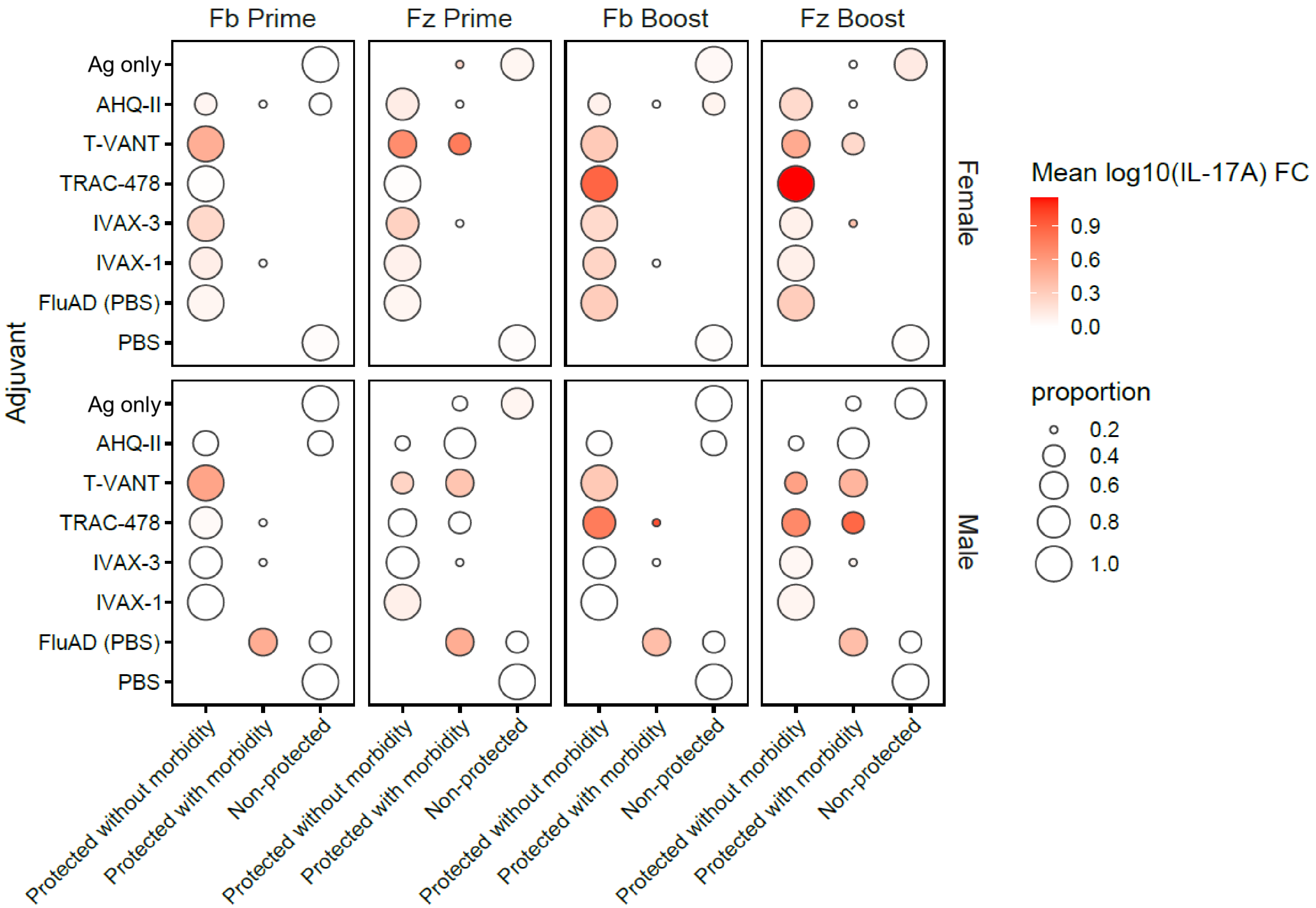

Figure S7L

IFN-β

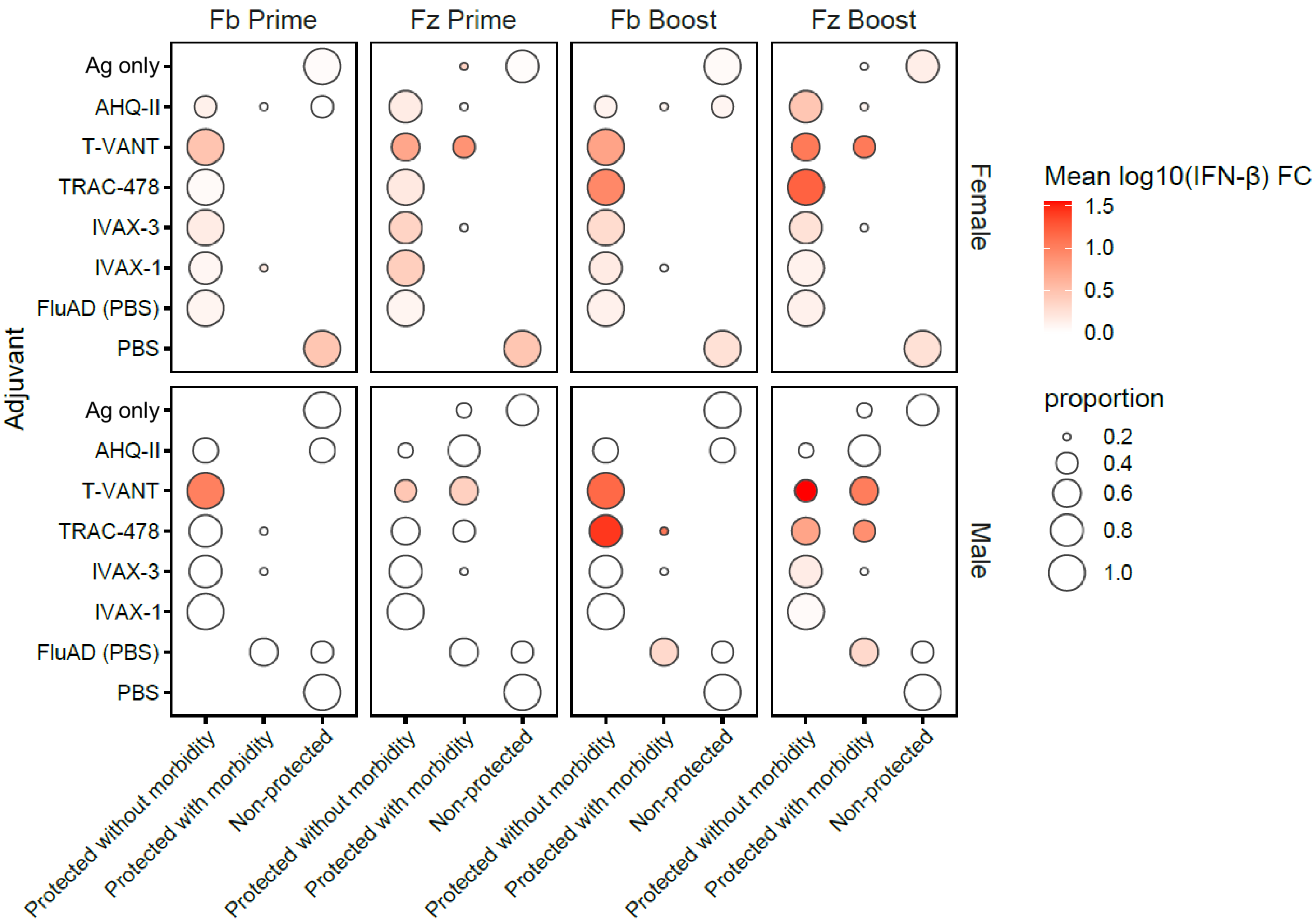

Figure S7M

GM-CSF

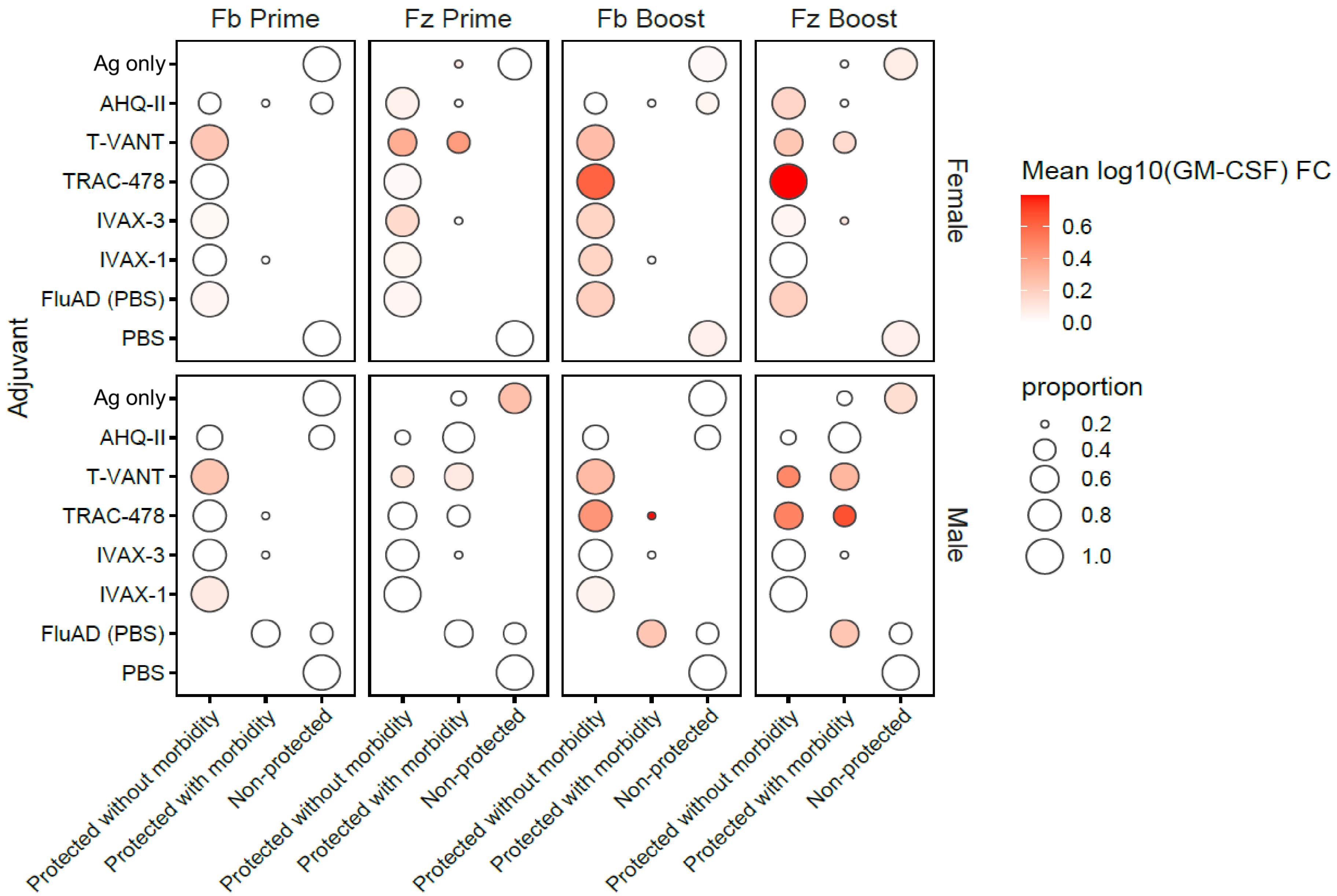

Supplement: Supplementary file 1 — Supplemental Informations [file 41541_2025_1339_MOESM1_ESM.pdf]
